# Cardiac GR Mediates the Diurnal Rhythm in Ventricular Arrhythmia Susceptibility

**DOI:** 10.1161/CIRCRESAHA.123.323464

**Published:** 2024-03-27

**Authors:** Roman Tikhomirov, Robert H. Oakley, Cali Anderson, Yirong Xiang, Sami Al-Othman, Matthew Smith, Sana Yaar, Eleonora Torre, Jianying Li, Leslie R. Wilson, David R. Goulding, Ian Donaldson, Erika Harno, Luca Soattin, Holly A. Shiels, Gwilym M. Morris, Henggui Zhang, Mark R. Boyett, John A. Cidlowski, Pietro Mesirca, Matteo E. Mangoni, Alicia D’Souza

**Affiliations:** Division of Cardiovascular Sciences (R.T., C.A., S.A.O., M.S., S.Y., L.S., H.A.S., G.M.M., A.D.), The University of Manchester, United Kingdom.; Department of Physics and Astronomy (Y.X., H.Z.), The University of Manchester, United Kingdom.; Bioinformatics Core Facility (I.D.), The University of Manchester, United Kingdom.; Division of Diabetes, Endocrinology and Gastroenterology (E.H.), The University of Manchester, United Kingdom.; Signal Transduction Laboratory, National Institute of Environmental Health Sciences, National Institutes of Health (R.H.O., J.L., L.R.W., D.R.G., J.A.C.).; Institut de Génomique Fonctionnelle, Université de Montpellier, Centre National de la Recherche Scientifique (CNRS), Institut National de la Santé et de la Recherche Médicale (INSERM), F-34094 Montpellier France (E.T., P.M., M.E.M.).; Department of Cardiology, John Hunter Hospital, Newcastle, NSW, Australia (G.M.M.).; Faculty of Life Sciences, University of Bradford, United Kingdom (M.R.B.).; Myocardial Function Section, National Heart and Lung Institute, Imperial College London, United Kingdom (R.T., M.S., A.D.).

**Keywords:** arrhythmias, cardiac, circadian rhythm, glucocorticoids, ion channels, transcription factors, receptors, glucocorticoid

## Abstract

**BACKGROUND:**

Ventricular arrhythmias (VAs) demonstrate a prominent day-night rhythm, commonly presenting in the morning. Transcriptional rhythms in cardiac ion channels accompany this phenomenon, but their role in the morning vulnerability to VAs and the underlying mechanisms are not understood. We investigated the recruitment of transcription factors that underpins transcriptional rhythms in ion channels and assessed whether this mechanism was pertinent to the heart’s intrinsic diurnal susceptibility to VA.

**METHODS AND RESULTS:**

Assay for transposase-accessible chromatin with sequencing performed in mouse ventricular myocyte nuclei at the beginning of the animals’ inactive (ZT0) and active (ZT12) periods revealed differentially accessible chromatin sites annotating to rhythmically transcribed ion channels and distinct transcription factor binding motifs in these regions. Notably, motif enrichment for the glucocorticoid receptor (GR; transcriptional effector of corticosteroid signaling) in open chromatin profiles at ZT12 was observed, in line with the well-recognized ZT12 peak in circulating corticosteroids. Molecular, electrophysiological, and in silico biophysically-detailed modeling approaches demonstrated GR-mediated transcriptional control of ion channels (including *Scn5a* underlying the cardiac Na^+^ current, *Kcnh2* underlying the rapid delayed rectifier K^+^ current, and *Gja1* responsible for electrical coupling) and their contribution to the day-night rhythm in the vulnerability to VA. Strikingly, both pharmacological block of GR and cardiomyocyte-specific genetic knockout of GR blunted or abolished ion channel expression rhythms and abolished the ZT12 susceptibility to pacing-induced VA in isolated hearts.

**CONCLUSIONS:**

Our study registers a day-night rhythm in chromatin accessibility that accompanies diurnal cycles in ventricular myocytes. Our approaches directly implicate the cardiac GR in the myocyte excitability rhythm and mechanistically link the ZT12 surge in glucocorticoids to intrinsic VA propensity at this time.

**Novelty and Significance**

**What Is Known?**

- The incidence of ventricular arrhythmia (VA) and sudden cardiac death exhibits a diurnal rhythm (morning peak) for which the underlying cause is uncertain.
- Rhythmic expression of selected ion channels driven by circadian clock transcription factors has been linked to diurnal changes in cardiac electrical excitability.

**What New Information Does This Article Contribute?**

- Mouse plasma corticosterone peaks in the biological morning resulting in increased binding to the glucocorticoid receptor (GR) in ventricular cardiomyocytes, increased GR translocation to the nucleus, increased GR binding to open chromatin profiles of key ion channels such as *Scn5a* and *Kcnh2*, and consequently increased expression of the ion channels and corresponding ionic currents, I_Na_ and I_Kr_.
- Mice exhibit an analogous morning peak in VA susceptibility, and biophysically detailed computer modeling shows that this is attributable to day-night rhythms in SCN5A and KCNH2, their corresponding ionic currents, I_Na_ and I_Kr_, and rhythmic expression of the gap junction protein Connexin 43.
- Systemic GR antagonism or cardiac-specific knockout of the GR abolished the day-night transcriptional rhythms in SCN5A, KCNH2, and Connexin 43 and the morning propensity to VA.

Epidemiological studies have demonstrated that the risk of VA is not constant throughout the day but instead occurs in a circadian pattern with a prominent peak in the morning hours and a sleep time nadir. In this study, we introduce the cardiac GR as a new and critical player in the timing mechanism responsible for the intrinsic VA propensity of the heart. We demonstrate that GR nuclear translocation and recruitment to precise and evolutionarily conserved open chromatin profiles in the biological morning determine a proarrhythmic electrophysiological substrate due, in part, to transcriptional control of 2 ion channels (SCN5A and KCNH2) and a gap junction protein (Connexin 43) essential to cardiac conduction and repolarization in human hearts. Pharmacological block of the GR or cardiomyocyte-specific knockdown of GR signaling abrogated ion channel rhythms and subsequently morning VA propensity, indicating GR targeting as a potential new chronotherapeutic strategy to suppress VA occurrence.

**Meet the First Author, see p 1233**

**Editorial, see p 1327**

Sudden cardiac death due to ventricular arrhythmia (VA) is the most common cause of death worldwide, given to account for 15% to 20% of all deaths.^1^ Intriguingly, epidemiological studies have shown that sudden death and VA presentation demonstrate a prominent time-of-day dependence, being more common in the morning on waking.^2^ A diurnal rhythm in VA is apparent in both ischemic and nonischemic heart diseases and in heritable and acquired arrhythmia syndromes,^3–9^ implying the salient participation of certain circadian factors in VA onset. Two primary mechanisms may reasonably be considered to underpin diurnal rhythms in VA susceptibility: (1) acute (ie, short-term) modulation of cardiac electrophysiology through neurohumoral factors such as cortisol and sympathetic signaling,^10,11^ which show a robust day-night rhythm, peaking at or immediately before the onset of activity, and (2) day-night changes in the cardiac electrophysiological substrate due to a day-night rhythm in the expression of key ion channels that determine the ventricular action potential. The latter mechanism has received attention in recent years, and studies in rodent models have demonstrated that day-night rhythms in the electrophysiological properties of the heart (eg, heart rate, QT interval, and action potential parameters) are accompanied by rhythmic expression of at least 10 different cardiac ion channels or regulatory subunits, including the Na^+^ channel (*Scna5*^12^ underlying I_Na_), a Ca^2+^ channel (*Cacna1c*;^13^ I_Ca,L_), K^+^ channels (*Kcnd2* and *KChIP2*,^14^ *Kcna5*,^15^ and *Kcnh2*^16^ underlying I_to_, I_K,ur_, and I_Kr_), a hyperpolarization-activated cyclic nucleotide–gated channel (*Hcn4*;^17^ I_f_), and gap junction channels (*Gja1* and *Gja5*^18^ underlying electrical coupling). However, in terms of the corresponding ionic currents, day-night rhythms have only been reported in I_Kur_^15^ and I_Ca,L_^13^ thus far, and how the summation of day-night rhythms in multiple ion channel subunits impacts the ventricular action potential and the electrophysiological substrate that underpins the VA propensity at the start of the awake period is currently unexplored.

Endogenous transcriptional rhythms in ion channels and other proteins essential for normal cardiomyocyte function currently are understood to be orchestrated by a molecular oscillator mechanism (circadian clock) comprising transcription-translation feedback loops that cycle with a periodicity of 24 hours. In relation to the day-night control of cardiac electrical activity, the role of *Bmal1* (basic helix-loop-helix ARNT-like protein 1), a transcription factor (TF) that heterodimerizes with the TF circadian locomotor output cycles kaput (CLOCK) to activate gene expression, has been emphasized. Through mechanisms that are not fully understood, cardiomyocyte-specific knockout of *Bmal1* has been reported to dampen day-night rhythms in the transcription of ion channels and Ca^2+^-handling molecules in the ventricles^12,16,19^ and also reduce the density of I_Na_^12^ and I_Kr_.^16^ Although such loss-of-function studies in cardiomyocyte-specific circadian clock TF knockout mice have become a gold standard for demonstrating circadian control of cardiac electrical activity, studies in extra-cardiac tissues have revealed a complex regulatory mechanism in which clock TF-DNA binding is required but is not sufficient for transcriptional activation and that other factors, including favorable reorganization of chromatin structure, enhancer-enhancer interactions, and differential recruitment of nonclock TFs to tissue-specific accessible chromatin, play important roles.^20–24^

In this study, we set out to explore, for the first time, chromatin accessibility and unbiased TF involvement in day-night rhythms in ventricular myocytes and to assess the functional relevance of this regulation for the intrinsic excitability rhythms in the ventricles. Assay for transposase-accessible chromatin with sequencing (ATAC-seq) combined with molecular, electrophysiological, in silico, pharmacological, and transgenic tools led us to identify a new role for the cardiac glucocorticoid receptor (GR; transcriptional effector of glucocorticoid steroid hormone cortisol^25^ in humans/corticosterone in mice) as an important endocrine mediator of day-night rhythmicity in the heart. We demonstrate day-night patterns in genome-wide chromatin accessibility in ventricular myocytes and that GR recruitment to distinct evolutionarily conserved sites in open chromatin profiles likely mediates rhythmic transcription of ion channel subunits underpinning I_Na_ and I_Kr_ that are critical to conduction and repolarization in the ventricles. Importantly, we mechanistically link these changes to the intrinsic day-night rhythmicity in arrhythmia propensity, highlighting a new and fundamental role for a noncanonical circadian clock TF in the temporal control of ventricular excitability.

## METHODS

### Data Availability

Full methodological details are given in the Supplemental Material. Data supporting the findings of this study are available from the corresponding authors upon reasonable request. Please see the Major Resources Table in the Supplemental Materials.

## RESULTS

### Differential Chromatin Accessibility Across the Day-Night Cycle in Ventricular Myocytes

**Figure 1.**
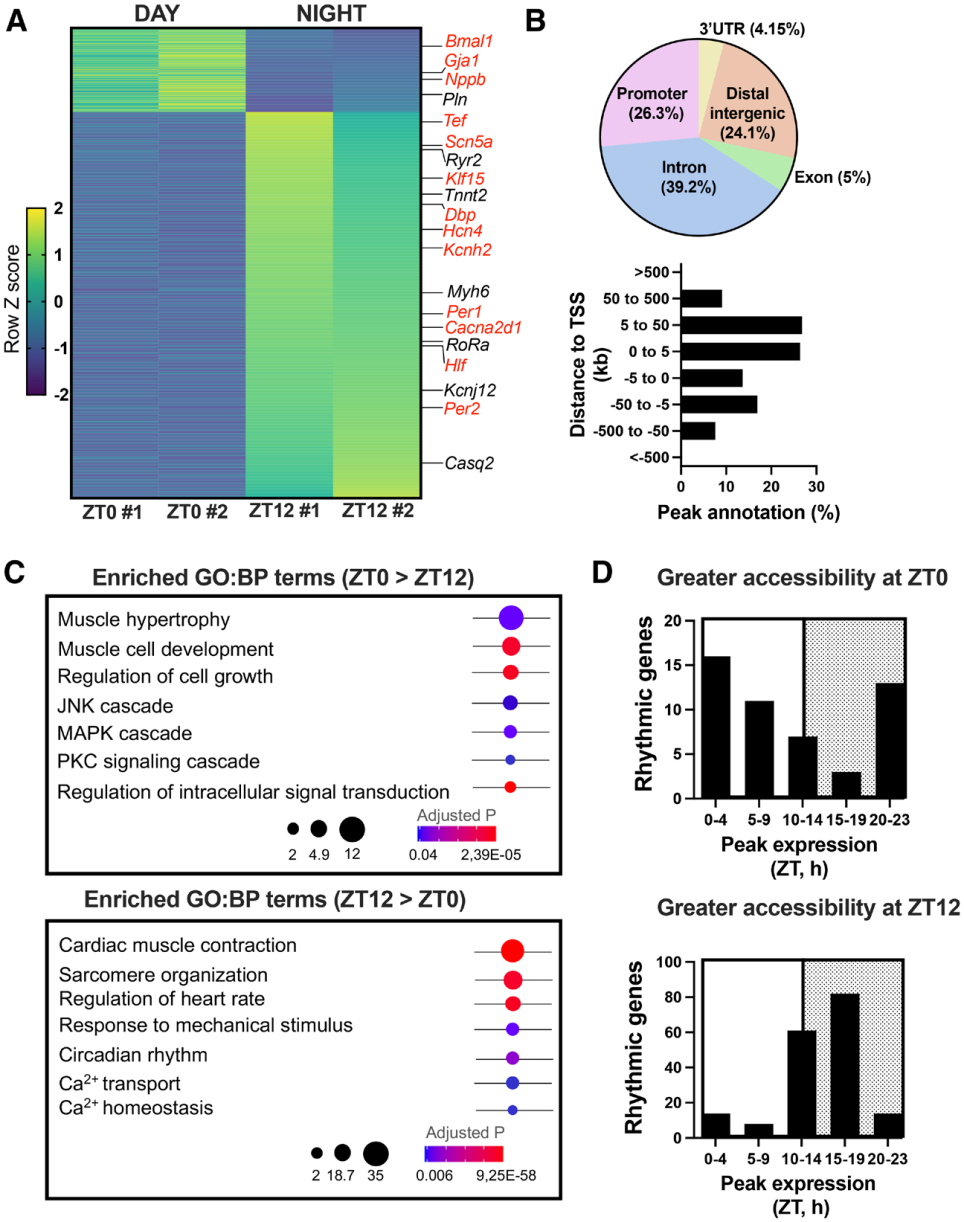
**Day-night variation in chromatin accessibility in ventricular cardiomyocyte nuclei. A**, Heatmap depicting 1111 day-night differentially accessible (DA) chromatin regions from assay for transposase-accessible chromatin with sequencing (ATAC-seq) performed in PCM1^+^ mouse left ventricular myocyte nuclei. Two independent biological replicates per time point are shown, and each replicate was composed of left ventricle samples pooled from 3 mice. Regions annotating to core circadian clock transcription factors (TFs) and genes essential for ventricular excitability or contractility are highlighted; in some cases (those highlighted in red), qPCR confirmed that transcript expression demonstrated a 24-hour rhythm (JTK_CYCLE–adjusted *P*<0.05) and peak expression coincided with the time point (ZT0 or ZT12) where chromatin was more accessible. **B** (**Top**), Pie chart of genomic region annotation for DA peaks (promoter=−1/+0.1 kb of transcription start site [TSS]). **B** (**Bottom**), Histogram denoting distance of ATAC-seq peaks to the associated TSS. **C**, Enrichment dot plots for gene ontology (GO):biological process pathways annotated from DA peaks more accessible at ZT0 and ZT12. Selected terms relevant to cardiomyocyte function are given. Gene count enriched in the pathway is denoted by dot diameter, and dot color shows the pathway enrichment significance. All presented pathways were significantly enriched (Binomial test, false discovery rate [FDR]–adjusted *P*<0.05). **D**, Histogram of gene expression phase for rhythmic genes (JTK_CYCLE–adjusted *P*<0.05; from Zhang et al^28^) in which DA chromatin regions were identified at (**top**) ZT0 and (**bottom**) ZT12. BP indicates biological process; MAPK, mitogen-activated protein kinase; PKC, protein kinase C; qPCR, quantitative polymerase chain reaction; ZT, zeitgeber time; ZT0, time of lights on; and ZT12, time of lights off.

As ≈95% of TFs bind in open chromatin regions,^26,27^ we performed ATAC-seq that enables the identification of genome-wide open chromatin profiles and an unbiased view of TF binding motifs enriched in these regions (Figure 1A). ATAC-seq libraries were generated from myocyte nuclei isolated from adult male C57BL/6J mouse left ventricles harvested at the time of lights off, start of awake period (ZT12, active phase in nocturnal mice housed under standard light-dark conditions) and compared with nuclei isolated from the left ventricle collected at the time of lights on, start of sleep period (ZT0, the inactive phase in mice). The selection of these time points was based on previous work that identified increased intrinsic VA vulnerability at ZT12 versus ZT0^19^ in mice, mirroring the early morning peak in the occurrence of VA and sudden cardiac death in humans. Pericentriolar Material 1 labeling^29^ followed by fluorescent-activated cell sorting was applied to purify the myocyte nuclei fraction (Supplemental Material; Figure S1) from 2 biological duplicates per time point. Each duplicate comprised 3 pooled left ventricle biopsies. All ATAC-seq libraries showed a high degree of concordance overall (Figure S1I). An average of ≈100 000 regions of accessible chromatin (ie, ATAC-seq peaks) per replicate per time point were identified, which overall annotated to similar genomic regions. Differential analysis using DiffBind (*P*≤2.09×10^−3^; false discovery rate–corrected *P*<0.1) revealed 1111 peaks with significant quantitative differences at the 2 time points, and, intriguingly, 83% were more accessible at ZT12 (Figure 1A). Figure 1B (top) shows that consistent with previous studies examining chromatin dynamics in noncardiac tissues,^26,30,31^ the majority of differentially accessible (DA) regions were intronic (39%) followed by promoter (26%) and intergenic (24%) regions. All DA regions were located within 500 kb from the nearest transcriptional start site, in line with potential regulatory roles (Figure 1B, lower). Comparison with a publicly available chromatin immunoprecipitation sequencing data set^32^ for H3K27ac or acetylation of the lysine residue at the N-terminal position 27 of the histone H3 protein in adult mouse hearts, demonstrated that 74% of DA regions overlapped with H3K27ac marks commonly borne by active enhancers, further indicating their potential importance as regulatory elements (data not shown). Annotation of DA regions to candidate genes followed by gene ontology analysis conducted using Genomic Regions Enrichment of Annotations Tool (GREAT)^33^ revealed significant (false discovery rate <0.05, Binomial test) enrichment of key processes regulating contractility, Ca^2+^-handling, and electrical activity at ZT12 (Figure 1C, bottom), in line with greater physiological demand at this time point and aligning with the view that biological tasks are preferentially confined to specific phases over a 24-hour period.^34^ On the other hand, DA chromatin regions enriched at ZT0 were annotated to biological processes associated with signaling cascades and cell development (Figure 1C, top).

To assess whether transcripts annotating to DA regions demonstrated day-night rhythms, we intersected our ATACseq data with high-resolution time-series DNA microarray data for ≈24 214 transcripts in mouse heart collected under constant dark conditions generated by Zhang et al^28^ available via the open-source platform CircaDB. Of the genes with DA chromatin profiles identified in this study, 37% had a significant day-night rhythm in the corresponding transcript (JTK_CYCLE^35^ adjusted *P*<0.05). Considering the genes with greater chromatin accessibility at ZT0, 56% demonstrated broad phase alignment between accessibility and transcript expression, that is, maximal expression during the early light period (≈ZT0-4) or the late dark period (≈ZT20-23; Figure 1D, top). Greater coherence between chromatin accessibility and transcript expression was determined at ZT12 when 80% of genes with greater accessibility were associated with maximal transcript expression in the early mid-dark period between ≈ZT12 and ZT18 (Figure 1D, lower). Time-of-day–dependent changes in selected transcripts (highlighted in red in Figure 1A) likely to be or known to be involved in the cardiac electrical excitability rhythm or the cardiomyocyte clock were investigated in further detail by carrying out qPCR (quantitative polymerase chain reaction) at 6 time points over a 24-hour period; once again, there was alignment between chromatin accessibility and peak transcript expression. These include cardiac ion channels (*Scn5a* and *Kcnh2*), circadian clock TFs (*Per2*, *Dbp*, and *Hlf*), and the zinc-finger DNA binding TF *Klf15* (key player in cardiac day-night rhythms), which peak at ≈ZT12, and the core circadian clock gene, *Bmal1*, and the gap junction channel *Gja1* (Cx43), which peak at ZT0 (Figure S2). It is concluded that chromatin accessibility at predominantly promoter distal sites accompanies expected day-night transcript expression profiles of core circadian clock genes, key ion channels, and a TF previously known to be involved in day-night rhythms in the ventricles.

### GR Motif Enrichment in DA Regions

**Figure 2.**
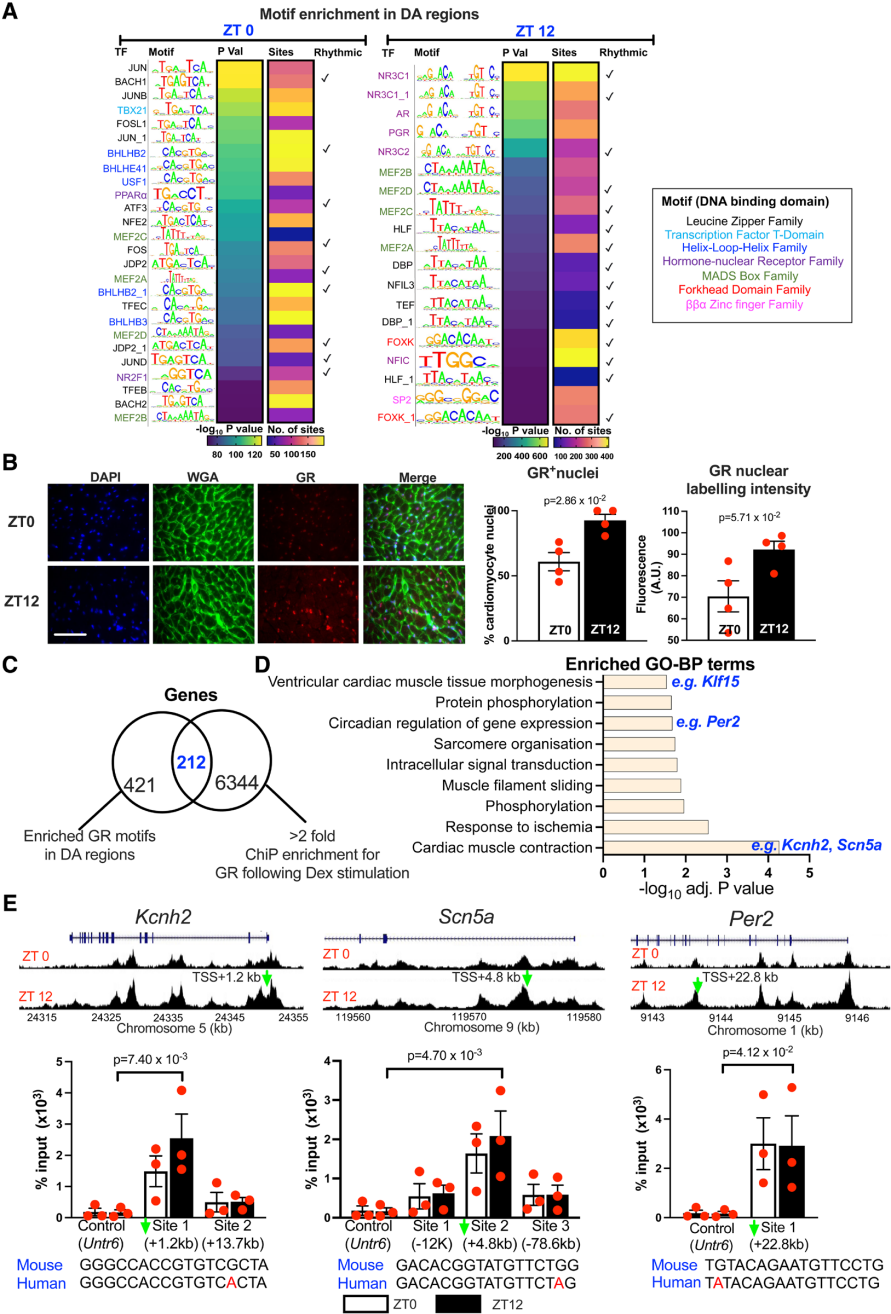
**GR enrichment at ZT12. A**, Columns show significantly enriched transcription factor (TF) motifs (color-coded to denote TF family) colored from yellow to purple with yellow being the most significant (SeqPos *Z* score, Binomial test). The number of binding sites was similarly colored with yellow denoting a greater number of sites. TFs associated with enriched motifs in which day-night rhythmicity has been previously established^28^ are highlighted with a checkmark. _1 denotes instances where >1 consensus motif for a particular TF was identified. **B** (**Left**), Representative GR immunofluorescent labeling of 10-µm sections of mouse left ventricular biopsies isolated at ZT0 and at ZT12. Cardiomyocytes were identified by wheat germ agglutinin staining and analyzed for GR nuclear localization (red signal) relative to the DAPI label (blue signal; 4 hearts per time point, ≈65 ventricular myocytes per animal analyzed. Scale bar, 20 µm. **B** (**Right**), Summary data derived from images in (**B**, **left**) show (1) the percentage of GR^+^ nuclei relative to the total number of DAPI^+^ cardiomyocyte nuclei analyzed and (2) the signal intensity of GR labeling. *P* value shown (Mann-Whitney *U* test). **C**, Venn diagram denoting 212 high-confidence GR targets obtained by intersecting assay for transposase-accessible chromatin with sequencing (ATAC-seq) with GR chromatin immunoprecipitation sequencing (ChIP-seq)^37^ data. **D**, Gene ontology (GO):biological process enrichment for 212 putative GR target genes derived from (**C**). (Binomial test, false discovery rate [FDR]–adjusted *P*<0.05.) **E** (**Top**), UCSC genome browser tracks of open chromatin regions at *Kcnh2*, *Scn5a*, and *Per2* loci. *Y* axis scale=0 to 5 counts per million reads for *Kcnh2* and *Scn5a* and 0 to 3 counts per million reads for *Per2*. Arrows denote predicted and subsequently ChIP-verified evolutionarily conserved GR binding motifs within differentially accessible (DA) regions. **E** (**Middle**), GR ChIP-qPCR assay testing GR occupancy of predicted genomic sites at *Kcnh2*, *Scn5a*, and *Per2* using chromatin from mouse left ventricle biopsies harvested at ZT0 and ZT12 (n=3 per time point). ChIP enrichment for sites of interest and negative control shown. Each point is an independent biological replicate. Data were normalized for primer efficiency by carrying out qPCR for each primer pair with input DNA isolated and pooled from all samples. The arrow denotes sites with significant ChIP enrichment relative to negative control. *P* value is shown (Kruskal-Wallis test with the Dunn multiple comparisons test). **E** (**Bottom**), Sequence conservation of GR-occupied sites at *Kcnh2*, *Scn5a*, and *Per2*. Aligned human GR sites were obtained from the UCSC Genome Browser. Nonconserved bases are highlighted in red. BP indicates biological process; DAPI, 4′,6-diamidino-2-phenylindole; dex, dexamethasone; GR, glucocorticoid receptor; qPCR, quantitative polymerase chain reaction; UCSC, University of California Santa Cruz; WGA, wheat germ agglutinin; ZT0, time of lights on; and ZT12, time of lights off.

To identify candidate TFs involved in transcriptional rhythms, we performed motif enrichment analysis on all DA regions using the Cistrome platform based on the Galaxy open-source framework.^36^ Consensus motifs for several TF families were identified in DA regions, and a comparison with CircaDB showed that the transcript expression of many of the associated TFs demonstrated day-night rhythms in the mouse heart (Figure 2A). At both time points, enrichment for MEF2 (myocyte-specific enhancer factor-2) motifs within the MADS-box family was observed, consistent with its enrichment in cardiomyocyte-specific open chromatin profiles.^38^ Interestingly, immediate early gene TF (eg, FOS [protein c-Fos] and JUN) motifs were enriched at ZT0, whereas TF motifs belonging to the Hormone Nuclear Receptor family (NR3C1, the GR, and NR3C2, the mineralocorticoid receptor) and Leucine zipper PAR-bZip family of D-box binding circadian clock output TFs (DBP [albumin D-binding protein], HLF [hepatic leukemia factor], TEF [thryotroph embryonic factor]) were overrepresented at ZT12. The enrichment of motifs for D-box binding TFs at ZT12 was in line with previous studies of D-box enhancer activity^39^ and with the known day-night expression of genes encoding these TFs (eg, *Dbp* and *Hlf*; Figure S2).

We hypothesized that TFs corresponding to these differentially enriched motifs may have implications for day-night gene expression patterns and rhythmic changes in ventricular electrical excitability as a consequence. We focused efforts on the GR—the most abundant and statistically significant (binomial test; *P*=1.00 x10^−69^) candidate TF consensus sequence enriched in ZT12 DA regions. The GR is a ligand-gated TF that, on glucocorticoid (ie, cortisol in humans and corticosterone in rodents) binding, enters the nucleus to modulate transcription by binding to glucocorticoid response elements in promoters or enhancers of target genes^40^ including ion channels.^41,42^ Because glucocorticoid secretion follows a pronounced circadian profile, peaking at the onset of the awake period in both diurnal (eg, humans) and nocturnal (eg, mice)^43^ animals and GR activation by exogenous administration of the synthetic glucocorticoid dexamethasone phase resets clock genes in the heart^44–46^; we reasoned that the GR was ideally placed to mediate excitability timing cues both by direct regulation of ion channel transcription and indirectly through the local clock. To assess this, we first examined whether the GR exhibited a day-night variation in nuclear translocation. GR immunofluorescence analysis was performed on left ventricular free wall sections derived from hearts harvested at ZT0 and ZT12 (4 hearts per group; Figure 2B). Consistent with our motif analysis, the number of GR^+^ nuclei (GR^+^DAPI^+^ labeling) was significantly increased in hearts isolated at ZT12 versus ZT0, and there was a trend (*P*=5.71×10^−2^) toward an increase in fluorescent intensity of nuclear GR labeling at ZT12 compared with ZT0 (Figure 2B, right). Experiments were performed to confirm a 24-hour rhythm in GR nuclear translocation by analyzing GR nuclear labeling in hearts collected at ZT0, four hours after lights on (ZT4), eight hours after lights on (ZT8), ZT12, four hours after lights off (ZT16) and eight hours after lights off (ZT20). A pronounced diurnal rhythm in the number of GR^+^ nuclei (JTK_CYCLE–adjusted *P*=5.78×10^−4^) and GR labeling intensity (JTK_CYCLE–adjusted *P*=1.32×10^−2^) was determined (Figure S3) with a peak at ZT12, in line with the peak of the 24-hour rhythm in the availability of the endogenous GR ligand corticosterone (c.f. Figure 5B). Next, to associate the GR with mediators of cardiac electrical activity, we correlated GR binding sites identified in motif analysis with annotated genes. A GR binding region within 10 kb upstream or 10 kb downstream of the gene structure was considered associated with that gene and possibly has a regulatory influence on that gene’s transcription. We intersected that data set (421 genes) with a publicly available GR chromatin immunoprecipitation sequencing data set^37^ in which GR binding sites in 6344 genes were identified on dexamethasone treatment in neonatal rat ventricular cardiomyocytes (>2-fold enrichment versus control). A schematic diagram of this comparison is shown in Figure 2C from which a set of 212 genes with ChIP-identified GR binding sites demonstrated greater chromatin accessibility and GR motif enrichment at ZT12. Gene ontology analysis of this gene set revealed significant enrichment of processes highly relevant to rhythms in ventricular activity, including the circadian clock and cardiac muscle contraction (Figure 2D). Notable components of these enriched pathways included (1) ion channel transcripts, *Scn5a* and *Kcnh2*, which are transcribed rhythmically, known to underpin ventricular myocyte excitability and repolarization, and have been investigated extensively in the context of life-threatening drug–induced and inherited arrhythmia syndromes,^47–49^ and (2) established GR targets, *Per2* (a core clock TF)^50^ and *Klf15*,^51^ a TF that is linked to ventricular day-night rhythms. To directly address GR occupancy within predicted motifs for these targets of interest, GR ChIP followed by qPCR was performed in mouse ventricular biopsies harvested at ZT0 and ZT12 (3 biological replicates per time point) using primers spanning our computationally predicted 15-bp GR consensus sequence motifs centered on the transcription start site of each candidate target gene (primer sequences given in the Supplemental Material). Genomic segments bearing GR consensus sequence motifs were also aligned with the corresponding segment of the human genome. Figure 2E shows ATACseq open chromatin profiles showing the location of evolutionarily conserved GR motifs for which significant ChIP enrichment versus control (negative control primer pair that amplifies regions in a gene desert on chromosome 6, *Untr6*) was determined. Consistent with previous studies in the mouse hepatic system, Figure 2E (right) shows GR occupancy at the *Per2* locus within a predicted intronic region 22.8 kb downstream from the transcription start site. This region has been previously verified to be the functional site by which the GR exerts control over *Per2* transcriptional rhythmicity with continuous GR occupancy over the day-night cycle.^50^ At the *Kcnh2* locus, motif analysis identified 2 GR binding sites, and we observed significant ChIP enrichment for the conserved site located +1.2 kb from the transcription start site (Figure 2E, left). Similarly, we identified 3 putative GR binding sites on the *Scn5a* locus and verified significant GR occupancy versus control at the (conserved) site located ≈5 kb upstream from the transcription start site (Figure 2E, middle). For these genes and also *Klf15* (Figure S4), GR binding tended to mirror greater ligand availability at ZT12. These data indicate resonance between the oscillating systemic glucocorticoid signal and expression patterns of genes pertinent to cardiomyocyte excitability, linked by GR binding to evolutionarily conserved sites enriched in chromatin in which there is greater accessibility at ZT12 versus ZT0.

**Figure 3.**
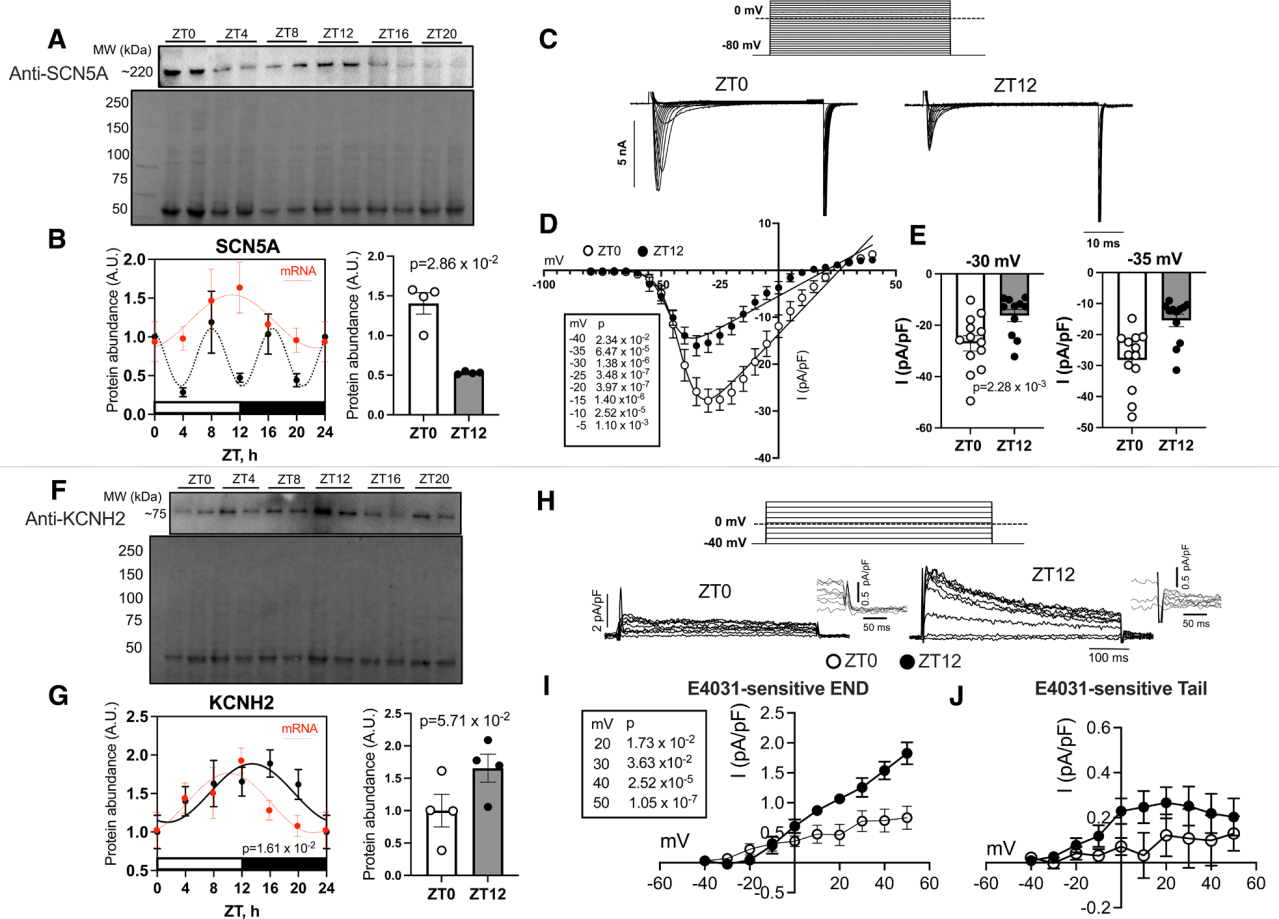
**Functional consequences of the day-night rhythm in GR target genes. A**, Representative SCN5A western blot from left ventricular free wall biopsies isolated at ZT0, ZT4, ZT8, ZT12, ZT16 and ZT20. Corresponding stain-free total protein gel was used for quantification and is shown in (**bottom**). **B** (**Left**), SCN5A protein expression from western blots. Data are from n=4 mice per time point and pooled from 2 sets of independent experiments. In this and all similar graphs, light and dark phases are indicated by the horizontal bars. Protein expression is normalized to that at ZT0. SCN5A mRNA abundance (red symbols) from Figure S2 is overlaid for comparison of the mRNA and protein time courses. **B** (**Right**), western blot data showing SCN5A expression from n=4 mice per time point at ZT0 and ZT12. *P* value derived from the Mann-Whitney *U* test is given. **C**, Representative traces of I_Na_ in isolated ventricular myocytes at (**left**) ZT0 and (**right**) ZT12 evoked by depolarizing pulses shown in the inset (**top**). **D**, Current-voltage relationships for I_Na_ at ZT0 (open circles) and ZT12 (filled circles) fitted by employing the Boltzmann relation: I_Na_=G_max_(V−V_rev_)/{1+exp[(V_0.5,act_−V)/s]}, where V_rev_ is the extrapolated reversal potential, V is the membrane test potential, I_Na_ is the current at given voltage, G_max_ is the cell maximum conductance, V_0.5,act_ is the voltage for half current activation, and s is the slope factor of the Boltzmann relation. Each point represents the mean±SEM of 13 cells from 3 mice at each time point. *P* values from linear effects mixed model followed by the Sidak multiple comparisons test given in the inset. **E**, Mean±SEM I_Na_ peak density at holding potentials of −30 and −35 mV. *P* value was computed from a nested *t* test. **F**, Representative KCNH2 western blot from left ventricular free wall biopsies isolated at ZT0, ZT4, ZT8, ZT12, ZT16, and ZT20. Corresponding stain-free total protein gel was used for quantification and is shown in (**bottom**). **G** (**Left**), KCNH2 protein expression from western blots. Data are from n=4 mice per time point. Protein expression is normalized to that at ZT0. KCNH2 mRNA abundance (red symbols) from Figure S2 is overlaid for comparison of the mRNA and protein time courses. **G** (**Right**), western blot data showing KCNH2 expression from n=4 mice per time point at ZT0 and ZT12. *P* value derived from the Mann-Whitney *U* test is given. **H**, Representative traces of I_Kr_ defined as E-4031 sensitive current at ZT0 and ZT12. Insets show I_Kr_ tails on current reactivation. Corresponding current-voltage relationships for I_Kr_ measured at the end of the depolarizing pulse (END) are shown in (**I**), and I_Kr_ measured at the peak of the tail (tail) on reactivation is shown in (**J**). Data are from n=3 mice at ZT0 (open circles) and n=3 mice at ZT12 (filled circles). Each point represents the mean±SEM of 8 cells at ZT0 and 9 cells at ZT12. *P* values from the linear effects mixed model followed by the Sidak multiple comparisons test are given. GR indicates glucocorticoid receptor; ZT0, time of lights on; ZT4, four hours after lights on; ZT8, eight hours after lights on; ZT12, time of lights off; ZT16, four hours after lights off; and ZT20, eight hours after lights off.

**Figure 4.**
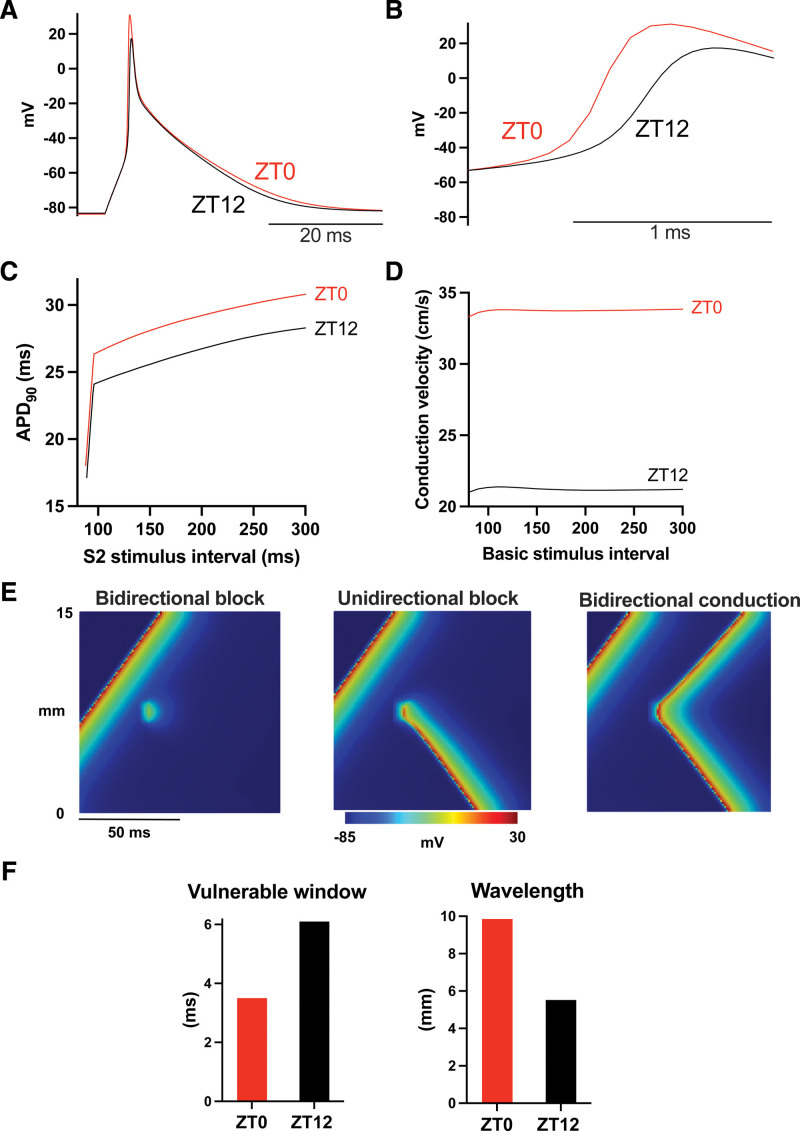
**Impact of the day-night rhythm in ion channels on the ventricular action potential and arrhythmia susceptibility at ZT12. A**, Action potentials at a basic stimulus interval of 150 ms at ZT0 and ZT12. **B**, Action potentials in (**A**) shown at an expanded time scale enabling comparison of the upstroke velocity of the action potential at ZT0 and ZT12. **C**, Action potential restitution curves at ZT0 and ZT12. Action potential duration at 90% repolarization (APD_90_) is plotted against the S2 stimulus interval (basic stimulus interval, 300 ms). **D**, Conduction velocity as a function of the basic stimulus interval at ZT0 and ZT12. **E**, 2-dimensional plots of distance against time with the membrane potential color-coded following an S1 stimulus (at the basic stimulus interval of 200 ms) and an S2 stimulus at different stimulus intervals of 50, 55, and 60 ms at ZT0. **F** (**Left**), Width of the vulnerability window at a basic stimulus interval of 200 ms at ZT0 and ZT12. **F** (**Right**), Wavelength of excitation waves at a basic stimulus interval of 200 ms at ZT0 and ZT12. ZT0 indicates time of lights on; and ZT12, time of lights off.

### Predicted Proarrhythmic Consequences of the Day-Night Variation in Ion Channels With Functional GR Binding Sites

Direct transcriptional targeting of *Scn5a* and *Kcnh2* is a potential mechanism by which the GR could mediate rhythmic ventricular myocyte excitability. Although rhythmic transcription of *Scn5a* and *Kcnh2* (Figure S2) has been reported previously,^12,16,28^ whether protein levels of these channels demonstrate daily rhythms is currently unknown, and functional data demonstrating a day-night rhythm in the density of the corresponding ionic currents are lacking. The circadian phases of protein abundance are frequently distinct from the phases of corresponding transcripts^52,53^; for example, protein abundance may peak hours before mRNA abundance,^52^ explained by rhythms in phase, amplitude, and average rate coefficients of processes that underpin both protein production and degradation (protein half-life).^54^ Protein expression of SCN5A/Na_v_1.5 and KCNH2/K_v_11.1 was assessed by western blotting in left ventricular biopsies harvested at 6 time points at 4-hour intervals over a 24-hour cycle. Representative blots are shown in Figure 3A and 3F. Protein expression (normalized to total protein and to the ZT0 value to enable comparison between blots) is plotted against time in Figure 3B and 3G. Although there was no appreciable 24-hour rhythm in SCN5A accumulation, protein expression at ZT0 was significantly higher than that at ZT12 (Figure 3B, right). Immunolabeling experiments using antibodies against SCN5A were performed to further investigate the apparent increase in SCN5A at ZT0 versus ZT12. Analysis of SCN5A^+^ labeling in 1227 ventricular cardiomyocytes in 20 sections from 4 mice (5 sections per mouse) at ZT0 compared with 985 ventricular cardiomyocytes analyzed in 20 sections from 4 mice (5 sections per mouse) indicated a trend toward increased SCN5A^+^ cell membrane labeling at ZT0 compared with ZT12 (Figure S5A and S5B) although the difference did not achieve statistical significance. In the case of KCNH2, a distinct 24-hour rhythm was discernible (JTK_CYCLE–adjusted *P*=1.61×10^−2^) with a ≈4-hour delay appreciable in the mRNA peak at ZT12 and the protein peak at ZT16 (Figure 3G, left). To assess the functional relevance of the observed day-night variation in SCN5A and KCNH2 protein expressions, we performed patch-clamp recordings from myocytes isolated at ZT0 and ZT12. At ZT12, the peak density of I_Na_ at a test potential of −30 mV was reduced by ≈42% in comparison to that observed at ZT0 (Figure 3C–3E), qualitatively consistent with the changes in protein described above. In contrast, the E-4031-sensitive I_Kr_ density measured at the end of the depolarizing pulse was ≈150% higher at ZT12 than at ZT0 (Figure 3H–3J), once again in line with the rhythm in protein expression of KCNH2. There was no statistically significant difference in the density of 4-aminopyridine-sensitive I_to_ or TEACl-sensitive I_K_^55^ at ZT0 and ZT12 in this study although there was a trend toward an increase in the density of I_to_ at ZT12 at positive voltages (Figure S6). It is concluded that there are functionally relevant day-night differences in the expression levels of SCN5A and KCNH2.

**Figure 5.**
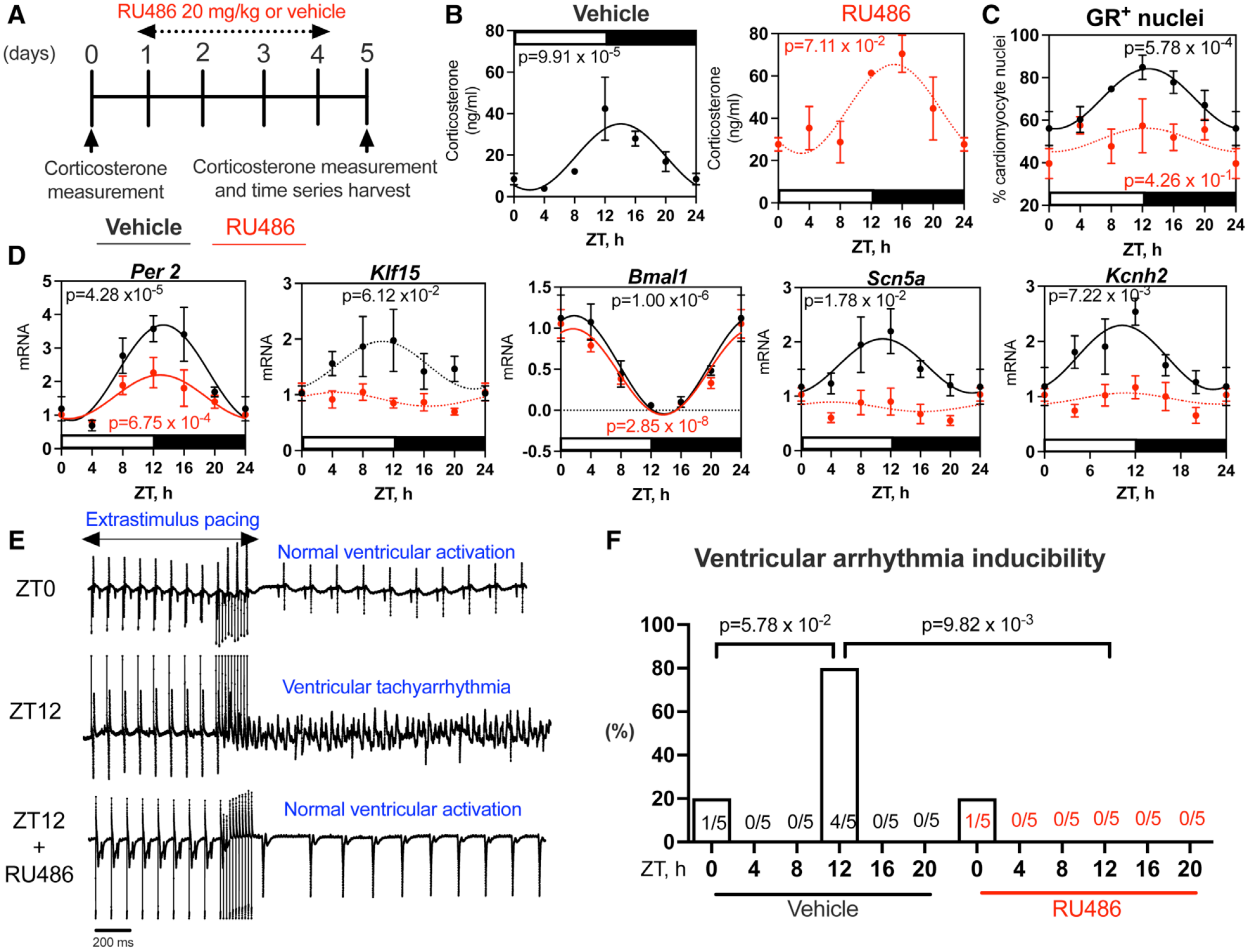
**Effect of chronic pharmacological GR block on ion channel rhythms and ventricular arrhythmia (VA) susceptibility. A**, RU486 dosing strategy and experimental design (n=5 mice per group for all parameters). **B**, Plasma corticosterone measured using ELISA from blood samples collected at ZT0, ZT4, ZT8, ZT12, ZT16 and ZT20 following 4 days of vehicle (**left**) or RU486 (**right**) intraperitoneal injection. In this and all similar figures, a significant day-night rhythm (as determined by JTK_CYCLE; *P* value shown) is denoted by the fitted sine wave (solid line). **C**, Summary data derived from immunofluorescent labeling studies showing the percentage of GR^+^ nuclei relative to the total number of DAPI^+^ cardiomyocyte nuclei analyzed at ZT0, ZT4, ZT8, ZT12, ZT16, and ZT20 in the vehicle (black) and RU486-treated (red) hearts. Data in the RU486 group were not rhythmic (JTK_CYCLE *P* value shown), but data are fitted with a sine wave for visual aid (dotted line). **D**, Expression of GR target genes measured by qPCR in mouse left ventricle biopsies at ZT0, ZT4, ZT8, ZT12, ZT16, and ZT20 following 4 days of vehicle (black) or RU486 (red) administration. Expression normalized to *Ipo8* and *Tbp*. Data are normalized to control ZT0 mean, and ZT0 is replotted as ZT24 as a visual aid. **E**, Representative ECG recordings of VA in vehicle-treated Langendorff-perfused mouse hearts triggered by programmed electrical stimulation at ZT12 but not at ZT0 and protection from pacing-induced VA in mice treated with 20-mg/kg RU486 daily for 4 days before termination. S1 cycle length=98 ms and S2 to S10 cycle length=18 ms in representative records shown. **F**, Summarized VA susceptibility given as a percentage of inducible VA in vehicle- and RU486-treated mice. The *P* value shown was determined by a χ^2^ test. DAPI indicates 4′,6-diamidino-2-phenylindole; GR, glucocorticoid receptor; qPCR, quantitative polymerase chain reaction; ZT0, time of lights on; ZT4, four hours after lights on; ZT8, eight hours after lights on; ZT12, time of lights off; ZT16, four hours after lights off; and ZT20, eight hours after lights off.

**Figure 6.**
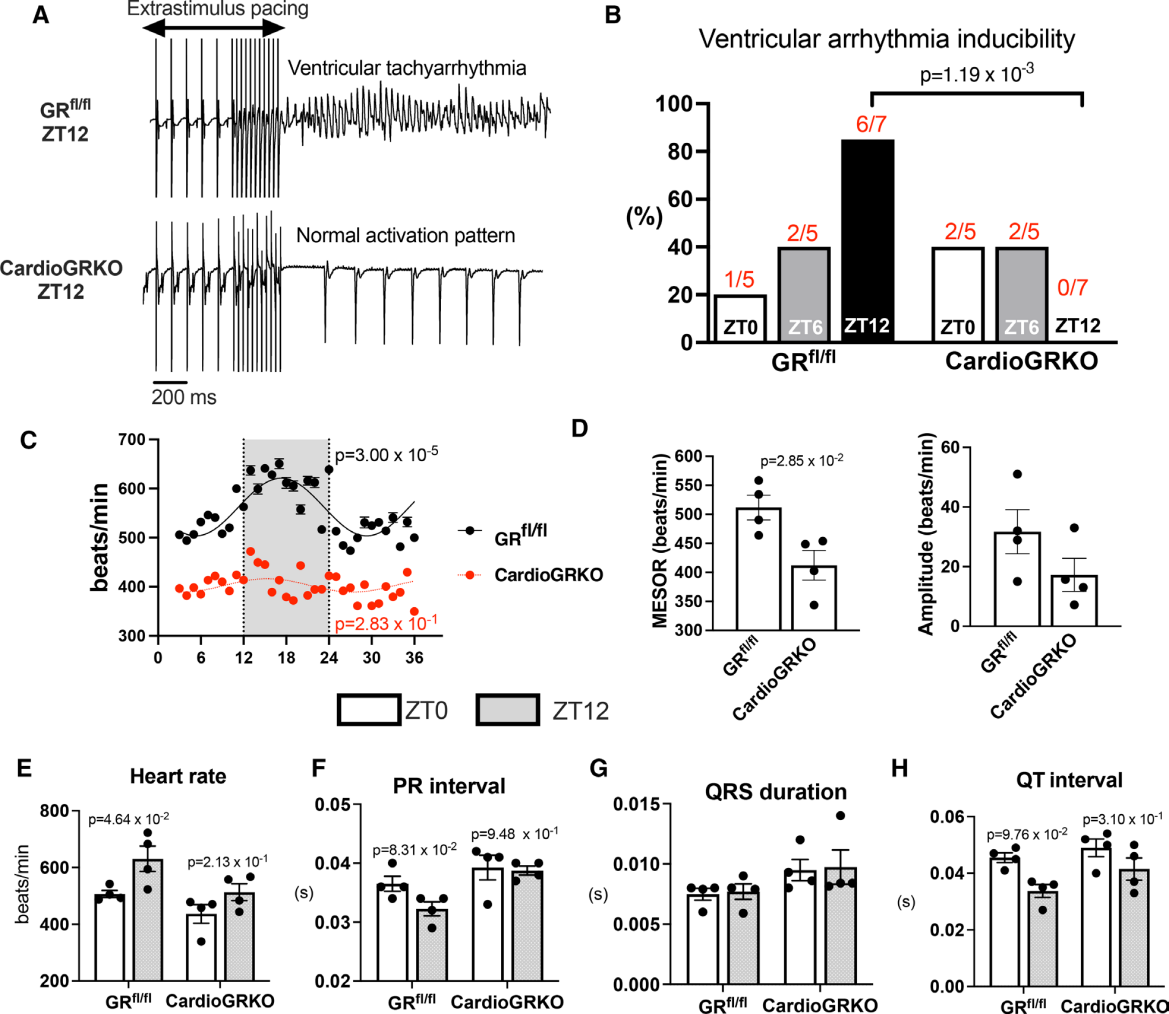
**Cardiac-specific GR knockout modifies day-night rhythms in ECG parameters and arrhythmia susceptibility. A**, Representative ECG recordings of ventricular arrhythmia (VA) in Langendorff-perfused GR^fl/fl^ mouse hearts triggered by programmed electrical stimulation at ZT12 and protection from pacing-induced VA at ZT12 in cardiomyocyte-specific GR knockout (cardioGRKO) mice. S1 cycle length=98 ms and S2 to S10 cycle length=38 ms in representative records shown. **B**, Summarized VA susceptibility given as a percentage in GR^fl/fl^ and cardioGRKO mice. VA induction in 5 to 7 hearts/group (*P* value shown, χ^2^ test). **C**, Representative heart rate data measured using biotelemetry in GR^fl/fl^ and cardioGRKO mice (n=4 per genotype) over a 36-h period. Light and dark-shaded regions represent light and dark phases. Data are fit with a standard sine wave. The adjusted *P* value denoting circadian rhythmicity (zero amplitude F test from the Cosinor analysis) is given. **D**, Midline Estimating Statistic of Rhythm (MESOR) and amplitude of the heart rate rhythm computed by the Cosinor analysis in GR^fl/fl^ and cardioGRKO mice (n=4/genotype). *P* value was determined by a Mann-Whitney *U* test. **E** through **H**, ECG intervals from telemetry data measured at ZT0 and ZT12 in GR^fl/fl^ and cardioGRKO mice (*P* values shown; 2-way ANOVA with the Tukey multiple comparisons test). GR indicates glucocorticoid receptor; ZT0, time of lights on; and ZT12, time of lights off.

We addressed the combined impact of measured and previously reported day-night rhythms (Table S1) in I_Na_, I_Ca,L_, I_to_, I_K,r_, I_NaCa_, and electrical coupling (determined by a day-night rhythm in Cx43; Table S1; Figures S2 and S5C and S5D) on the ventricular action potential and vulnerability to reentry using biophysically-detailed computer modeling. The mouse ventricular action potential model described by Morotti et al^56^ was used to simulate excitation at ZT0. At ZT12, the maximal ionic conductances were scaled (as shown in Table S2) based on the observations presented in Table S1. At ZT12, it is predicted that the maximum upstroke velocity of the action potential is slowed and the action potential amplitude reduced (Figure 4A and 4B; Table S3) as a result of the reduced density of I_Na_ at ZT12. Action potential duration at 90% repolarization (APD_90_) is predicted to be shorter at ZT12 (Figure 4A; Table S3), which can be seen more clearly in the restitution curves (Figure 4C). The decrease in APD_90_ at ZT12 is consistent with what is seen experimentally (Table S4). As a result of the reduction in maximum upstroke velocity of the action potential and also *Gja1* at ZT12, the conduction velocity is predicted to be reduced at ZT12 (Figure 4D; Table S3). Vulnerability to arrhythmia was tested in a 1-dimensional model of a string of ventricular cells (see detailed methods in the Supplemental Material). After stimulation at a basic stimulus (S1-S1) interval, a premature S2 stimulus after a variable interval was given. Figure 4E shows 2-dimensional plots of distance against time with the membrane potential color-coded. The S1 action potential (triggered at the basic stimulus interval) conducts at a constant velocity. If the S2 stimulus is given within the refractory period at the stimulus site (short S2 stimulus interval), no action potential is triggered (Figure 4E, left). At an appropriate longer S2 stimulus interval, cells at the stimulus site will no longer be refractory, and an action potential will be conducted retrogradely but not anterogradely because cells will still be refractory anterogradely (Figure 4E, center). The resultant unidirectional block is known to be arrhythmogenic and able to initiate a reentrant arrhythmia in a 2- or 3-dimensional network. At an appropriate even longer S2 stimulus interval, the S2 action potential will conduct both anterogradely and retrogradely because all cells will no longer be refractory (Figure 4E, right); this is no longer arrhythmogenic. The vulnerable window is the range of S2 stimulus intervals giving a unidirectional block. Figure 4F (left) shows the width of the vulnerability window; the width of the window is greater at ZT12 than ZT0, and this means that the ventricles will be more arrhythmogenic at ZT12. The volume of tissue able to sustain a reentrant wave of excitation depends on the wavelength: the length of active tissue (=APD_90_×conduction velocity). Our predictions indicate that the wavelength is shorter at ZT12 (Figure 4F, right), and, therefore, the mouse heart will be able to sustain a reentrant arrhythmia more effectively. It is concluded that the increased vulnerability to VA at ZT12^19^ (c.f. Figures 5F and 6B) can be explained by the observed day-night rhythm in ion channels.

### Pharmacological GR Block Dampens Ion Channel Rhythms and the Day-Night Variation in VA Susceptibility

Having identified a potential mechanism by which a day-night rhythm in *Scn5a* and *Kcnh2* expression can contribute to increased intrinsic ZT12 VA susceptibility, we directly assessed GR involvement in this process. We conducted experiments using the well-characterized competitive GR antagonist RU486, which binds with high affinity (dissociation constant ≤10^−9^ M) to the GR (and the progesterone receptor).^57^ Studies in humans and animal models have shown that RU486 impedes GR-mediated transcriptional responses by disrupting GR-DNA binding, inhibiting the recruitment of coregulatory proteins, and promoting the recruitment of transcriptional repressors.^57–59^ The half-life of RU486 has been estimated at 20 to 30 hours,^60,61^ and guided by previous efforts to chronically block the GR in mice, RU486 (20-mg/kg body weight)^62^ was injected intraperitoneally 6 hours before the well-recognized circadian peak of corticosterone secretion in male mice at ZT12 for 4 days (Figure 5A). Measurement of circulating corticosterone levels showed the expected diurnal rhythm in vehicle control mice and a trend (*P*=7.11×10^−2^) for this pattern in the RU486 group (Figure 5B) and that the dosing strategy did not significantly modify the amplitude of the day-night (ie, ZT0 versus ZT12) rhythm in plasma corticosterone (Figure S7). From immunolabeling experiments, it was confirmed that with chronic GR blockade, the 24-hour rhythm in GR translocation to the nucleus (Figure S3) was no longer discernible (Figure 5C). The impact of GR block on day-night rhythmicity of GR target transcripts of interest identified above (eg, *Per2*, *Klf15*, *Scn5a*, and *Kcnh2*) was assessed in qPCR experiments on ventricular samples isolated at 4-hour intervals over a 24-hour period. Figure 5D shows that in vehicle control mice, rhythmic variation in the expression the ion channel transcripts, *Kcnh2* and *Scn5a*, and the known GR circadian clock gene target, *Per2*, were observed (JTK_CYCLE–adjusted *P* value for the known GR gene target gene *Klf15* was 6.12×10^−2^); expression levels of these transcripts were higher at ZT12 relative to all other time points assessed. Strikingly, GR block blunted or abolished rhythmic expression in these transcripts (Figure 5D), consolidating the functionality of conserved GR motifs identified by our ATACseq studies (Figure 2E; Figure S4) and demonstrating that *Scn5a* and *Kcnh2* are bona fide targets of GR. Intriguingly, the rhythmic transcript expression of *Bmal1*, the currently accepted driver of transcriptional rhythms in *Scn5a* and *Kcnh2*^12,16^ and determinant of day-night VA susceptibility,^19^ was unaffected by RU486 treatment (Figure 5D). These data, combined with in silico predictions (Figure 4F), prompted the hypothesis that suppression of ion channel rhythmicity with GR blockade will dampen the day-night rhythm in VA susceptibility. To assess whether the GR block abrogates the day-night variation in the electrophysiological substrate that portends to increased ZT12 VA susceptibility, extra stimulus pacing^63^ was applied to trigger reentrant VA in Langendorff-perfused hearts from vehicle- and RU486-treated mice. A drive train of 20 S1 stimuli at a 98-ms cycle length was followed by an S2 to S10 train of extra stimuli starting with a cycle length of 58 ms and decreasing to 8 ms in 10-ms intervals with a 3 s gap between repeat periods of stimulation. Representative recordings (Figure 5E) show that in vehicle-treated mice, extra stimulus pacing–induced VA susceptibility peaked at ZT12. At this time point, 4 out of 5 hearts developed VA, characterized by rapid and chaotic activation patterns exceeding 3 s in length, whereas the same protocol applied at ZT0 evoked VA in only 1 out of 5 hearts. Remarkably, chronic GR blockade with RU486 abolished the ZT12 susceptibility to VA, wherein 5 out of 5 hearts tested were resistant to pacing-induced VA. Summary data demonstrating a trend (*P*=5.78×10^−2^) toward higher occurrence of VA at ZT12 versus ZT0 and significant protection from VA inducibility at ZT12 by pretreatment with RU486 are given in Figure 5F. VAs could not be evoked at ZT4, ZT8, ZT16, and ZT20 in either group. Experiments were also performed to rule out the possibility that GR activation triggers instantaneous arrhythmia susceptibility (ie, through nongenomic mechanisms^64^) by treating mice with 1-mg/kg dexamethasone at ZT0 and testing VA inducibility after 15 minutes. VAs were not inducible in either group under these conditions. Taken together, these findings demonstrate that the GR regulates intrinsic cardiomyocyte excitability rhythms in part at least by orchestrating rhythms in ion channel gene transcription.

### Cardiomyocyte-Specific Deletion of GR Also Abolishes the Day-Night Rhythm in VA Susceptibility

The finding that pharmacological GR blockade abrogated the ZT12 VA susceptibility of the heart encouraged a more detailed analysis of the role of the cardiomyocyte GR in the heart’s day-night rhythm. To this end, we generated cardiomyocyte-specific GR knockout (cardioGRKO) mice by crossing GR^fl/fl^ mice with αMHC^Cre/+^ mice.^65^ Cardiomyocyte GR knockout in the left ventricle was confirmed by RNAseq and qPCR (Figure S8), following which the day-night rhythm in VA susceptibility was investigated. We applied programmed electrical stimulation to Langendorff-perfused hearts isolated at 3 time points over the circadian cycle, ZT0, ZT6, and ZT12. Figure 6A and 6B shows that GR^fl/fl^ mice, when challenged with extra stimulus pacing, demonstrated day-night variation in susceptibility to VA comparable to that in wild-type mice (Figure 5F). In striking contrast, cardioGRKO mice—akin to mice in which the GR was blocked pharmacologically—were resistant to triggered VAs at ZT12. Programmed electrical stimulation provoked VAs in 6 of 7 GR^fl/fl^ hearts at ZT12, whereas the same pacing protocols failed to elicit VAs in any of the 7 cardioGRKO mice tested at this time point (Figure 6B). In sum, these data consolidate a new and specific role for the GR in the day-night rhythm in VA susceptibility.

As an aside, the ECG recorded in conscious and unrestrained mice by implantation of radiotelemetry devices revealed significant changes to the day-night variation in various ECG parameters in cardioGRKO mice compared with GR^fl/fl^ mice (Figure 6C–6H). Figure 6C shows the mean hourly heart rate recorded over a ≈36-hour cycle from GR^fl/fl^ mice and cardioGRKO mice. As expected, there was a distinct day-night rhythm in heart rate in GR^fl/f^ mice, peaking at ZT12 at the onset of the dark/active period, in line with previous observations made in wild-type mice by our laboratory^17,66^ and others^19^ (Figure 6C). However, this pattern was no longer discernible in cardioGRKO mice (Figure 6C). To assess day-night rhythmicity, cosine wave characteristics, including Midline Estimating Statistic of Rhythm (MESOR), acrophase (zeitgeber time at the peak), and amplitude (difference between the peak/trough and the mean), were evaluated using Cosinor analysis.^67^ A significant day-night rhythm in the heart rate (fitted cosine function *P*=3×10^−5^) was evident in 3 out of 4 GR^fl/fl^ mice but only 1 out of 4 cardioGRKO mice. The MESOR of heart rate was significantly reduced in the knockout group (Figure 6D), whereas the acrophase did not differ between groups. The amplitude of the cosine function trended toward a reduction in cardioGRKO versus GR^fl/f^ mice (Figure 6D, right panel), and, accordingly, the ZT12-ZT0 difference in heart rate was reduced by knockout of the GR (Figure 6E). Interestingly, a trend for a day-night rhythm in the PR and QT intervals was evident in GR^fl/fl^ mice but not in cardioGRKO mice (Figure 6F and 6H).

### Linking GR-Mediated Day-Night Transcriptomic Rhythmicity With Arrhythmia Susceptibility

**Figure 7.**
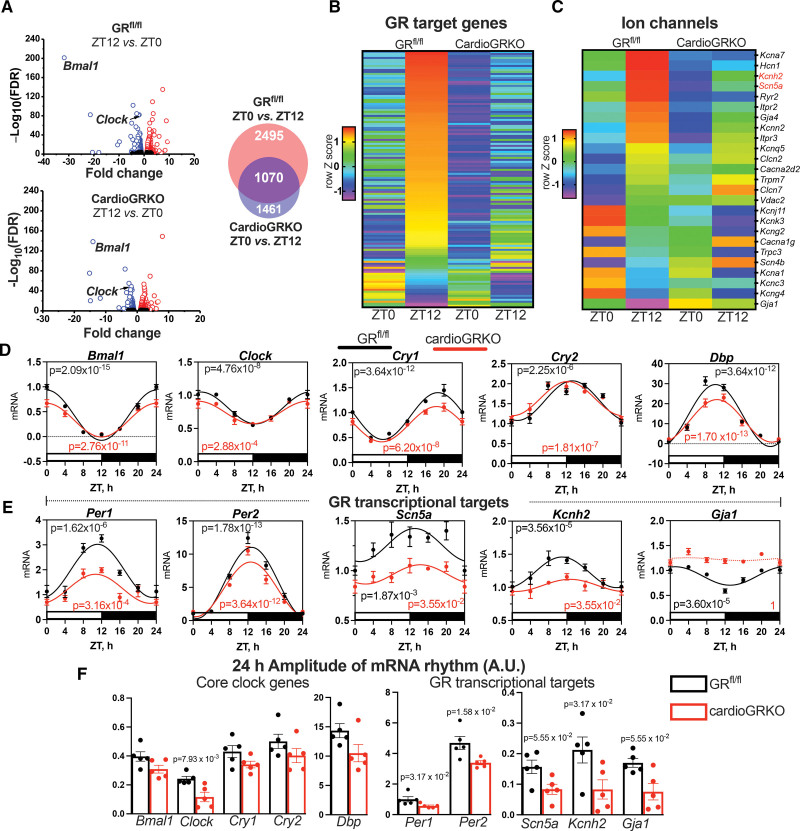
**Cardiomyocyte GR knockout modifies the day-night rhythm in the cardiac transcriptome despite persistent rhythms in core clock genes. A** (**Left**), Volcano plots representing ZT12 versus ZT0 differentially expressed genes (DEGs) in left ventricular biopsies from GR^fl/fl^ and cardiomyocyte-specific GR knockout (cardioGRKO) mice (n=5 per time point and per group) determined by RNAseq. Blue circles, downregulated DEGs; red circles, upregulated DEGs. Core clock transcription factors (TFs), *Bmal1* and *Clock*, shown. **A** (**Right**), Venn diagram denoting the overlap between DEGs in the 2 genotypes. **B**, Heatmap displaying mean value per time point for 421 genes measured by RNAseq at ZT0 and ZT12 in GR^fl/fl^ and cardioGRKO mice in which putative GR binding sites were identified by ATACseq and motif analysis. Data were *z*-scored by gene. Genes are ordered by time of maximal expression in the GR^fl/fl^ group. **C**, Heatmap displaying mean value per time point for all transcripts relating to ion channel/Ca^2+^ handling proteins that showed differential day-night expression in GR^fl/fl^ and cardioGRKO from RNAseq analysis. GR targets *Kcnh2* and *Scn5a* are highlighted in red. Data were *z*-scored by gene. **D** and **E**, mRNA expression measured in left ventricular free wall biopsies harvested at ZT0, ZT4, ZT8, ZT12, ZT16, and ZT20 in GR^fl/fl^ mice and CardioGRKO mice for core clock genes and GR targets of interest in which an altered ZT0/ZT12 amplitude was observed in GR^fl/fl^ mice vs CardioGRKO mice using RNAseq. Expression was normalized to *Ipo8* and *Tbp* (n=5 hearts per time point). ZT0 is replotted as ZT24 as a visual aid only. JTK_CYCLE–adjusted *P* values given for GR^fl/fl^ mice in black and CardioGRKO mice in red. A significant day-night rhythm (as determined by JTK_CYCLE) is further denoted by the fitted sine wave (solid line). For visual aid, a sine wave with a dotted line has been fitted for transcripts that were not rhythmic. **F**, JTK_CYCLE computed amplitude of 24-h rhythm of mRNA expression for genes given in (**D** and **E**). *P* values given determined by the Mann-Whitney *U* test. GR indicates glucocorticoid receptor; ZT0, time of lights on; ZT4, four hours after lights on; ZT8, eight hours after lights on; ZT12, time of lights off; ZT16, four hours after lights off; and ZT20, eight hours after lights off.

To probe GR-dependent mechanisms involved in ZT12 VA susceptibility, we performed RNAseq on left ventricular biopsies isolated at ZT0 and ZT12 from GR^fl/fl^ and cardioGRKO mice (5 mice per time point and genotype). We first examined the overall variability of the transcriptome (14 817 transcripts) using principal component analysis and observed clear clustering of samples according to genotype and time of day (Figure S9). Analysis of differentially expressed genes (DEGs) between ZT0 and ZT12 in GR^fl/fl^ and cardioGRKO mice (false discovery rate–corrected *P*<0.05) revealed that GR knockout decreased the number of DEGs by 41%, from 2495 DEGs in GR^fl/fl^ mice to 1461 DEGs in cardioGRKO mice (Figure 7A, right). Interestingly, *Bmal1* and *Clock* were among the most significant DEGs in both groups, suggesting that canonical core clock rhythmicity persists in cardioGRKO hearts (Figure 7A, left). This finding was confirmed by analyzing the expression of core clock genes at 6 time points over the circadian cycle using qPCR. Figure 7D and 7E shows that core clock genes demonstrated 24-hour rhythmicity in both GR^fl/fl^ and cardioGRKO groups. As expected, the amplitude of GR transcriptional targets *Per1* and *Per2* displayed a significant reduction in amplitude, along with *Clock* that was not identified as a direct GR target in this study (Figure 7F). Statistical analysis of ZT0 with ZT12 values within groups revealed a significant difference in all cases (*P*=0.01219, Mann-Whitney *U* test) with the exception of *Cry1* in the cardioGRKO group (*P*=0.8, Mann-Whitney *U* test).

We investigated 2 paradigms. First, we assessed whether the day-night expression pattern in genes with GR binding sites (predicted by our ATACseq data; Figure 2A) was altered by cardiomyocyte GR knockout. As expected, Figure 7B shows that expression of GR target DEGs was predominantly higher at ZT12 versus ZT0 in GR^fl/fl^ mice and this pattern was largely abrogated in cardioGRKO mice. As such, the number of GR target genes showing differential day-night expression was reduced by 63% in cardioGRKO versus GR^fl/fl^ mice, consolidating both the utility of our ATACseq approach and the involvement of the GR in the day-night expression pattern of these genes. Second, we investigated whether the day-night variation in key ion channel/Ca^2+^-handling transcripts/gap junction channels that underlie cardiac action potential generation and conduction was similarly affected. The heatmap in Figure 7C demonstrates that the ZT12 versus ZT0 increase in *Kcnh2* and *Scn5a* transcripts was diminished by GR knockout, consistent with direct GR target sites identified in Figure 2E and the impact of the sustained pharmacological block of the GR on the expression of these channels identified in Figure 5D. Similarly, the day-night rhythm in the gap junction transcript, *Gja1*, in GR^fl/fl^ mice was also abolished in the cardioGRKO mice (this is an interesting case because we did not observe GR binding sites associated with the *Gja1* gene, and, therefore, the regulation of *Gja1* may be an indirect effect of GR). The dampened rhythmicity of GR targets of interest *Scn5a*, *Kcnh2*, and *Gja1* in cardioGRKO mice was confirmed by qPCR studies conducted in samples harvested at 6 time point resolution (Figure 7E) and is borne out by comparing the amplitude of the 24-hour rhythmic patterns (Figure 7F). There was no longer a significant ZT0 versus ZT12 difference in the expression levels of these transcripts in cardioGRKO mice (Mann-Whitney *U* test; *P*>0.05).

Biophysically-detailed in silico modeling as used in Figure 4 was performed to test whether the loss of the day-night rhythms in *Scn5a*, *Kcnh2*, and *Gja1* in cardioGRKO mice (see Table S2 for scaling of maximal conductances) is sufficient to explain the loss of the increased vulnerability to VA at ZT12. Figure S10 demonstrates that the loss of the changes in cardioGRKO mice hearts at ZT12 is sufficient to restore the action potential upstroke velocity, action potential amplitude, APD_90_, and conduction velocity to ZT0 values. Correspondingly, both the width of the vulnerability window and the wavelength at ZT12 (important determinants of VA susceptibility) were restored to ZT0 levels in the cardioGRKO case (Figure S10D and S10E). These simulations provide insight into a mechanism by which cardioGRKO hearts are less likely to sustain triggered reentrant arrhythmia at ZT12.

GR binding sites on the *Scn5a* and *Kcnh2* genes are conserved between mice and humans (Figure 2E), and, therefore, it is possible that there is a day-night rhythm in *Scn5a* and *Kcnh2* transcription in the human as there is in the mouse. We simulated the human ventricular action potential using the biophysically detailed model described by ten Tusscher et al^68^ and incorporated the same day-night changes in ionic currents presumed to occur normally in the mouse and the modified changes in the knockout of GR (Table S2). The day-night changes in ionic currents presumed to occur normally produced a substantial day-night variation in APD_90_ consistent with the day-night rhythm in the QT_c_ interval in humans (Figure S11).^69^ The predicted decreases in maximum upstroke velocity of the action potential, action potential amplitude, and APD_90_ are all expected to contribute to an enhanced vulnerability to reentrant arrhythmias at ZT12 (start of the awake period). Suppressing the day-night rhythms in I_Na_ and I_Kr_ (expected effects of GR knockout) abolished these proarrhythmic changes (Figure S11). It is concluded that in humans as in mice, GR-mediated transcriptional control of ion channels may be responsible for the increased vulnerability to VA on waking in the morning.

### Additional Mechanisms by Which the Cardiac GR Modulates Day-Night Rhythms

**Figure 8.**
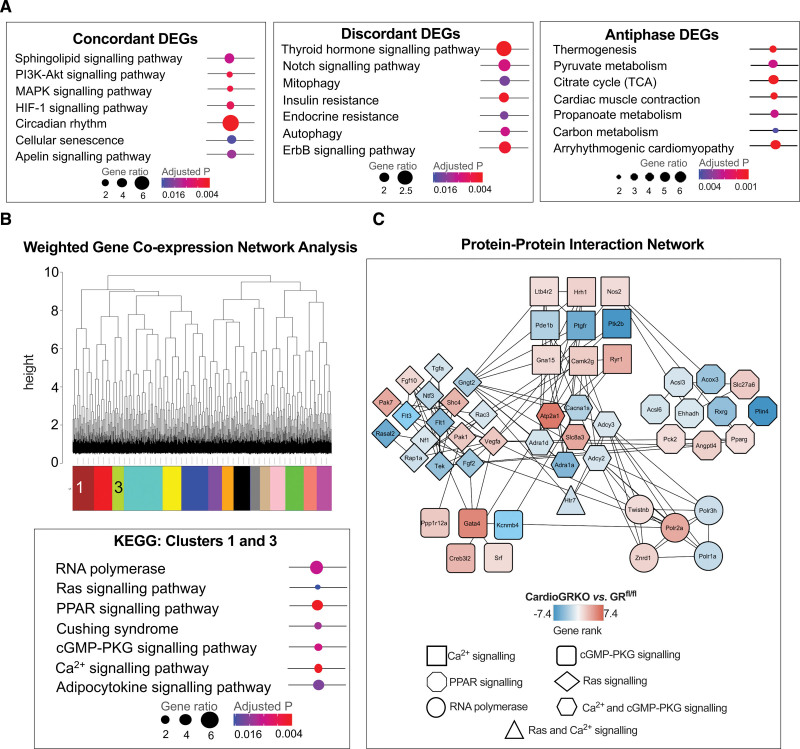
**Cardiomyocyte GR knockout modifies gene coexpression patterns. A**, Enrichment dot plots for KEGG pathway enrichment analysis for (**left**) concordant, (**middle**) discordant, and (**right**) antiphase differentially expressed genes (DEGs) in GR^fl/fl^ and cardiomyocyte-specific GR knockout (cardioGRKO) mice. The enrichment (gene) ratio is denoted by the dot diameter, and the dot color shows the pathway enrichment significance. All presented pathways were significantly enriched (false discovery rate [FDR] <0.05; Fisher exact test with Benjamini-Hochberg adjustment). **B** (**Top**), Cluster dendrogram determining coexpression clusters obtained by weighted gene coexpression network analyses performed on 7466 genes in GR^fl/fl^ and cardioGRKO mice detected by RNAseq. Each discrete cluster was assigned a unique color on the horizontal bar. Coexpressing clusters in cardioGRKO hearts were assigned numbers for visualization. **B** (**Bottom**), KEGG enrichment dot plot for modules 1 and 3 that were found to be differentially coexpressed in cardioGRKO hearts; 1157 genes from both modules were used as input data. The enrichment ratio is given by dot diameter, and the dot color shows the pathway enrichment significance. **C**, Protein-protein interaction (PPI) network plotted in Cytoscape using all component genes from selected KEGG pathways given in (**B**). Genes were uploaded to the STRING database to plot PPI; medium confidence for edges was chosen. The network was significantly enriched (*P*=8.05×10^−17^). Each node represents a gene, and the color gradient (from −7.4 represented in blue to 7.4 represented in red) represents the relative abundance of a gene in cardioGRKO versus GR^fl/fl^ samples at ZT12. Gene annotation to respective enriched KEGG pathway given by node shape. cGMP indicates cyclic guanosine monophosphate; ErbB, epidermal growth factor receptor tyrosine kinase; GR, glucocorticoid receptor; HIF-1, hypoxia-inducible factor-1; KEGG, Kyoto encyclopedia of genes and genomes; MAPK, mitogen-activated protein kinase; PKG, protein kinase G; PPAR, peroxisome proliferator activated receptor; TCA, tricarboxylic acid cycle; and ZT12, time of lights off.

Whereas this study has to date focused on direct transcriptional control of selected ion channels, it is well known that the GR controls numerous signaling cascades in the heart,^70^ mediating processes including but not limited to cardiomyocyte proliferation,^71^ metabolism,^72^ inflammation, and the stress response.^65^ Therefore, we utilized pathway and network analysis for system-level insight into regulatory networks that may contribute to responses of GR^fl/fl^ and cardioGRKO mice at ZT12. Kyoto Encyclopedia of Genes and Genomes (KEGG) pathway analysis^73^ was performed on DEGs at ZT12 and ZT0. Three types of DEGs were identified, as shown in Figure 8A: (1) concordant genes—DEGs showing a ZT12 versus ZT0 variation in both genotypes; (2) discordant genes—DEGs that showed ZT12 versus ZT0 variation in the GR^fl/fl^ group only; and (3) antiphase genes—DEGs in which expression patterns were reversed, that is, from being higher at ZT12 versus ZT0 in GR^fl/fl^ to higher at ZT0 versus ZT12 in the CardioGRKO group. Among the concordant genes, we observed prominent KEGG enrichment for terms relating to the circadian clock (including key clock TFs: *Bmal1*, *Per2*, *Per3*, *Clock*, *Cry2*, *Bhlhe41*, *Rora*, and *Npas2*) suggesting once again that the core circadian clock machinery remains functional in the GR knockout. Among discordant and antiphase genes, there was an overrepresentation of KEGG terms relevant to metabolism (component genes include *Bcat2*, the mitochondrial branched chained aminotransferase, and *Pparg*, the peroxisome proliferator-activated receptor γ, PPARγ, which has been shown to play a role in arrhythmogenesis^74,75^). Finally, because functionally related genes are often coregulated and coexpressed, weighted gene coexpression network analysis (see the Supplemental Material for full details)^76^ followed by KEGG pathway analysis and protein-protein interaction mapping^77^ of gene products was conducted to identify specific cellular processes/pathways that were impacted by GR knockout at ZT12. Weighted gene coexpression network analysis determined 14 gene clusters (Figure 8B, top), and differential coexpression analysis followed by permutation testing (Figure S12) determined that GR loss resulted in significant changes to coexpression patterns in the heart. For example, modules 1 and 3 in Figure 8B (top) clustered only in cardioGRKO but not GR^fl/fl^ mice suggesting that the constituent pathway components are differentially regulated in the 2 genotypes. We examined the associated biological functions of these modules using KEGG mapping and identified the enrichment of pathways relevant to the regulation of electrical homeostasis in cardiomyocytes including the Ca^2+^/cGMP-PKG pathway^78^ (component genes include those encoding the α-adrenoceptor^79^ and nitric oxide synthase 2^80^), the PPARγ (peroxisome proliferator-activated receptor γ) signaling pathway,^64^ and the Ras GTPase-P21 activated kinase 1^81^ signaling pathway. Protein-protein interaction mapping of cluster components annotating to selected KEGG terms is given in Figure 8C and resulted in a significantly enriched network (*P*=8.05×10^−17^; enrichment *P* values provided by the STRING database) with strongly intersecting nodes, consistent with our weighted gene coexpression network analysis clustering. Differential expression of the network components in cardioGRKO versus GR^fl/fl^ mice at ZT12 was appreciable (denoted by the color gradient in Figure 8C) indicating that the GR determines expression patterns of genes within weighted gene coexpression network analysis–identified and enriched pathways during the active phase of the animal at which time arrhythmia propensity is increased. Cumulatively, our approaches provide the first registration of GR involvement in day-night transcriptional responses of the heart and gene coexpression patterns and offer insight into attendant GR-dependent signaling pathways (eg, Ca^2+^-cGMP-PKG pathway, peroxisome proliferator-activated receptor γ signaling pathway, and Ras GTPase signaling pathway) that may also influence the day-night excitability rhythm in cardiomyocytes, beyond the direct targeting of selected ion channels and TFs that we have demonstrated in Figures 2, 5, and 7.

## DISCUSSION

This study provides the only evidence to date linking the GR to intrinsic day-night rhythms in cardiac ion channels and, consequently, the propensity to tachyarrhythmias that predispose to sudden cardiac death. We show that by binding within chromatin profiles that display time-of-day dependent accessibility, the GR modulates specific transcriptional cascades underpinning functionally relevant day-night rhythms in I_Na_ and I_Kr_. We also show that by an indirect route, GR is responsible for a day-night rhythm in *Gja1* expected to result in a functionally relevant day-night rhythm in electrical coupling. For the first time, we demonstrate the relevance of these changes for the heart-autonomous intrinsic active-phase susceptibility to reentrant tachyarrhythmia and establish that GR knockout in cardiomyocytes blunts day-night transcriptional remodeling and proarrhythmic changes to the cardiac substrate as a consequence. These data position the GR as a critical player in the timing mechanism that orchestrates both electrical homeostasis and intrinsic VA susceptibility of the heart.

The cardiomyocyte circadian rhythm is commonly assumed to enable anticipatory physiological adaptation via the expression of clock-related TFs in a 24-hour cycle. As such, all prior studies investigating the chronobiology of cardiac ion channels have focused primarily on the core clock components, BMAL1 and CLOCK, which bind DNA maximally at ≈ZT6.^21^ However, characterization of the BMAL1 cistrome indicates that only a small proportion of CLOCK:BMAL1 target genes are rhythmically expressed and many rhythmically expressed target genes are transcribed at night, in antiphase to maximal DNA binding.^21,82^ Therefore, additional mechanisms, involving cooperativity between the core circadian clock and noncore clock TFs,^21,23,24,82^ are critical to maintaining rhythms of functional importance. As a first foray into exploring this layer of regulation in cardiomyocytes, we performed ATAC-seq for unbiased genome-wide insight into TFs operant at ZT12, a time point at which the heart is particularly vulnerable to reentrant tachyarrhythmias. Our serendipitous identification of GR binding site enrichment in ZT12 open chromatin profiles for ion channels is significant for 2 reasons: not only does it highlight an unappreciated noncanonical clock TF mechanism that exerts significant regulatory control over day-night variation in the electrical activity of the heart but also presents the cardiac GR as a potential nodal regulator capable of transducing exogenous systemic signals to changes in the cardiac electrophysiological substrate in preparation for transition to activity in the awake period. Glucocorticoid release is clock-controlled,^83^ and GR is regarded as a conduit between the suprachiasmatic master clock and the circadian clocks in peripheral tissues, supported by evidence that synthetic steroids can transiently modify mRNA expression of core circadian clock genes (*Per1*, *Per2*, *Dbp*, *Clock*, and *Bmal1*),^44–46^ as well as GR function being modulated by the clock TFs CRY1/2^84^ and CLOCK.^85^ However, in this study, the GR block with RU486 did not impact rhythmic *Bmal1* expression patterns (Figure 5D), and, in cardioGRKO hearts, key clock genes continued to show expected day-night rhythms, albeit with dampened amplitude in the case of *Per* and *Clock* genes (Figure 7D–7F). Although these data suggest that the protection from ZT12 VA under conditions of GR blockade (Figure 5F) or cardiomyocyte-specific knockout (Figure 6B) cannot be fully attributed to disruption of the core circadian clock mechanism, additional evidence is now required to elucidate the bidirectional interaction and connectivity between the GR, its associated regulatory network of noncore clock TFs (eg, *Klf15*), and the cardiomyocyte clock. For example, it is possible that CLOCK:BMAL1 binding generates the permissive chromatin landscape^23^ enabling increased GR binding at ZT12, or GR expression is dependent on a functional clock. Indeed, the circadian peak of cortisol acts as an entrainment signal for peripheral clocks and, therefore, may indirectly modulate cellular responses to cortisol.^86^ Such cooperativity between the GR and the cardiomyocyte clock remains unaddressed and is an important area for further investigation.

While the present study is the first to define the physiological significance of GR activation in the day-night rhythm in cardiac excitability, prior work has linked glucocorticoid signaling with electrophysiological remodeling and arrhythmogenesis. Synthetic glucocorticoids are one of the most commonly used pharmaceuticals, and administration of these steroids has been linked to the presentation of cardiac arrhythmias, including sinus bradycardia,^87^ a ≈2-fold risk of atrial fibrillation or flutter,^88^ premature ventricular contractions, and nonsustained VT.^89^ Similarly, mice with cardiac overexpression of the human GR were presented with bradycardia, high-degree AV block, prolongation of the QRS interval, and increased QTc dispersion.^90^ Linking the GR with electrical remodeling, previous studies showed that GR activation with dexamethasone increased cardiac I_Kur_, I_K1_, and I_Ca,T_ through direct transcriptional activation of the underlying ion channel subunits.^41,42^ While these studies importantly demonstrated the regulation of ionic currents by the GR pathway, the impact of the day-night rhythm in the GR pathway had not previously been studied. In this study, we have linked the day-night rhythm in GR activation to the day-night rhythm in the ion channel subunits responsible for I_Na_ and I_Kr_. We have shown a lower I_Na_ density at ZT12 when GR activity is high. This result is in line with previous evidence, showing that the cardiac overexpression of GR diminishes I_Na_.^90^ The precise GR binding sites identified though motif analysis of ATACseq data and verified by ChIP explained our subsequent finding that sustained pharmacological block of the GR abolished rhythmic expression of *Scn5a* and *Kcnh2*, whereas genetic GR knockout dampened the amplitude of their day-night rhythm. Because a wealth of data from genetic studies has demonstrated that transcriptional control of *Scn5a* and *Kcnh2* is critical for conduction, repolarization, and arrhythmia susceptibility in humans,^91,92^ the finding that the GR exerts temporal regulation over these genes through binding sites conserved in humans and mice (evolutionary distance=90 million years) is of particular translational relevance. Building on these findings may also help explain the increased arrhythmia burden reported in human conditions of cortisol excess^93^ or deficiency.^94^

## CONCLUSIONS

This study provides new insight into the day-night rhythms in the electrical properties of the heart, identifying resonance between an oscillating systemic glucocorticoid signal and temporal control of cardiac electrical excitability linked by the cardiomyocyte GR. We have shown how the day-night rhythms in the electrical properties of the heart influence the early morning susceptibility to VA and sudden cardiac death, but it is likely that the heightened early morning susceptibility to VA and sudden cardiac death is only important in patients with heart disease, requiring autonomic trigger mechanisms acting on a structurally remodeled myocardium—this remains to be studied in a yet-to-be-defined model system encapsulating such complex interactions. As physicians increasingly consider the timing of medication to improve treatment outcomes, our findings introduce the prospect that cardiac GR-targeting chronotherapeutic strategies may help prevent the early morning propensity to VA.

## ARTICLE INFORMATION

### Sources of Funding

This work was supported by a British Heart Foundation Intermediate Fellowship (FS/19/1/34035) and a British Heart Foundation Project (PG/22/10919) to A. D’Souza, and a British Heart Foundation Programme Grant to M.R. Boyett and A. D'Souza (RG/18/2/33392). C. Anderson, S. Al Othman, and M. Smith were supported by the British Heart Foundation PhD Studentships (grants FS/17/67/33483, FS/18/62/34183, and FS/CRTF/23/24469). The studies were also supported by the Intramural Research Program of the National Institutes of Health, NIEHS (grant ZIAES090057) to J.A. Cidlowski and a Fondation Leducq TNE FANTASY (19CV03) grant to M.R. Boyett, M.E. Mangoni, and A. D’Souza.

### Disclosures

None.

### Supplemental Material

Expanded Materials and Methods

Figures S1–S12

Tables S1–S4

References 95–123

## Supplementary Material


