# Supplementary material for: Cardiac GR Mediates the Diurnal Rhythm in Ventricular Arrhythmia Susceptibility
Source: Circ Res. 2024 Mar 27;134(10):1306–26. doi: 10.1161/CIRCRESAHA.123.323464 (PMC11081863; doi:10.1161/CIRCRESAHA.123.323464)
Supplement: Supplementary file 1 [file res-134-1306-s001.pdf]

NOTE: Please save this file locally before filling in the table, DO NOT work on the file within your internet browser as changes will not be saved. dobe crobat eader (available free here) is recommended for completion.

The ARRIVE guidelines 2.0: author checklist

The ARRIVE Essential 10

These items are the basic minimum to include in a manuscript. Without this information, readers and reviewers cannot assess the reliability of the findings.

Item		Recommendation	Section/line number, or reason for not reporting
**Study design**	1	For each experiment, provide brief details of study design including:<br>a. The groups being compared, including control groups. If no control group has been used, the rationale should be stated.<br>b. The experimental unit (e.g. a single animal, litter, or cage of animals).	a. Online data supplement, page 1, lines 11-12, line 25.<br>b. Online data supplement, page 1, lines 11-13, line 30.
**Sample size**	2	a. Specify the exact number of experimental units allocated to each group, and the total number in each experiment. Also indicate the total number of animals used.<br>b. Explain how the sample size was decided. Provide details of any *a priori* sample size calculation, if done.	a. Online data supplement, page 1, lines 4-40; Major resources table and Statistical table.<br>b. Online data supplement, page 9, lines 449-451.
**Inclusion and exclusion criteria**	3	a. Describe any criteria used for including and excluding animals (or experimental units) during the experiment, and data points during the analysis. Specify if these criteria were established *a priori*. If no criteria were set, state this explicitly.<br>b. For each experimental group, report any animals, experimental units or data points not included in the analysis and explain why. If there were no exclusions, state so.<br>c. For each analysis, report the exact value of *n* in each experimental group.	a. Online data supplement, page 1, lines 16-21; Major resources table.<br>b. Online data supplement, page 1, lines 18-19; Major resources table.<br>c. Online data supplement page 1, line 30-31; Figure legends 1-8, Supplemental figure legends 1-13; Statistical table.
**Randomisation**	4	a. State whether randomisation was used to allocate experimental units to control and treatment groups. If done, provide the method used to generate the randomisation sequence.	a. Online data supplement, page 1, lines 24-26; Major resources stable.

		b. Describe the strategy used to minimise potential confounders such as the order of treatments and measurements, or animal/cage location. If confounders were not controlled, state this explicitly.	b. Online data supplement, page 1, lines 24-28; Major resources table.
**Blinding**	5	Describe who was aware of the group allocation at the different stages of the experiment (during the allocation, the conduct of the experiment, the outcome assessment, and the data analysis).	Online data supplement page 1, lines 26-28.
**Outcome measures**	6	a. Clearly define all outcome measures assessed (e.g. cell death, molecular markers, or behavioural changes).<br>b. For hypothesis-testing studies, specify the primary outcome measure, i.e. the outcome measure that was used to determine the sample size.	a. Online data supplement pages 1-10, lines 4-495.<br>b. Online data supplement pages 1-10, lines 4-495.
**Statistical methods**	7	a. Provide details of the statistical methods used for each analysis, including software used.<br>b. Describe any methods used to assess whether the data met the assumptions of the statistical approach, and what was done if the assumptions were not met.	a. Online data supplement pages 9-10, lines 449-495; Figure legends 1-8; Supplemental figure legends 1-13; Statistical table.<br>b. Online data supplement pages 9-10, lines 449-495.
**Experimental animals**	8	a. Provide species-appropriate details of the animals used, including species, strain and substrain, sex, age or developmental stage, and, if relevant, weight.<br>b. Provide further relevant information on the provenance of animals, health/immune status, genetic modification status, genotype, and any previous procedures.	a. Online data supplement page 1, lines 4-39; Major resources table.<br>b. Online data supplement page 1, lines 4-39; Major resources table.
**Experimental procedures**	9	For each experimental group, including controls, describe the procedures in enough detail to allow others to replicate them, including:<br>a. What was done, how it was done and what was used.<br>b. When and how often.<br>c. Where (including detail of any acclimatisation periods).<br>d. Why (provide rationale for procedures).	a-d. Online data supplement page 1, lines 4-39; Major resources table.
**Results**	10	For each experiment conducted, including independent replications, report:<br>a. Summary/descriptive statistics for each experimental group, with a measure of variability where applicable (e.g. mean and SD, or median and range).<br>b. If applicable, the effect size with a confidence interval.	a. Online data supplement page 9, lines 449-495; Figure legends 1-8; Supplemental figures 1-13; Statistical table.<br>b. Online data supplement page 9, lines 449-495; Figure

			legends 1-8; Supplemental figures 1-13.

The Recommended Set

These items complement the Essential 10 and add important context to the study. Reporting the items in both sets represents best practice.

Item		Recommendation	Section/line number, or reason for not reporting
**Abstract**	11	Provide an accurate summary of the research objectives, animal species, strain and sex, key methods, principal findings, and study conclusions.	Animal species, strain and sex not stated due to space constraints.
**Background**	12	a. Include sufficient scientific background to understand the rationale and context for the study, and explain the experimental approach.<br>b. Explain how the animal species and model used address the scientific objectives and, where appropriate, the relevance to human biology.	a. Introduction, pages 4-5, lines 27-75.<br>b. Introduction, pages 4-5, lines 27-75; Results, pages 5-15, lines 83-470.
**Objectives**	13	Clearly describe the research question, research objectives and, where appropriate, specific hypotheses being tested.	Research questions are clearly stated before each experiment in method descriptions and results description: Online data supplement pages 4-10, lines 4-495; Results, pages 5-15, lines 83-470.
**Ethical statement**	14	Provide the name of the ethical review committee or equivalent that has approved the use of animals in this study, and any relevant licence or protocol numbers (if applicable). If ethical approval was not sought or granted, provide a justification.	Online data supplement page 1, lines 4-8.
**Housing and husbandry**	15	Provide details of housing and husbandry conditions, including any environmental enrichment.	Online data supplement page 1, lines 12-15.
**Animal care and monitoring**	16	a. Describe any interventions or steps taken in the experimental protocols to reduce pain, suffering and distress.<br>b. Report any expected or unexpected adverse events.<br>c. Describe the humane endpoints established for the study, the signs that were monitored and the frequency of monitoring. If the study did not have humane endpoints, state this.	a. Online data supplement page 1, line 16.<br>b. Online data supplement page 1, line 18.<br>c. Online data supplement

			page 1, line 18.
**Interpretation/ scientific implications**	17	a. Interpret the results, taking into account the study objectives and hypotheses, current theory and other relevant studies in the literature.<br>b. Comment on the study limitations including potential sources of bias, limitations of the animal model, and imprecision associated with the results.	a. Results and discussion pages 5-17, lines 83-551.<br>b. Online data supplement page 1, lines 32-40.
**Generalisability/ translation**	18	Comment on whether, and how, the findings of this study are likely to generalise to other species or experimental conditions, including any relevance to human biology (where appropriate).	Results and discussion pages 5-17, lines 83-551.
**Protocol registration**	19	Provide a statement indicating whether a protocol (including the research question, key design features, and analysis plan) was prepared before the study, and if and where this protocol was registered.	Online data supplement page 1, line 22.
**Data access**	20	Provide a statement describing if and where study data are available.	Methods, page 5, line 77.
**Declaration of interests**	21	a. Declare any potential conflicts of interest, including financial and non-financial. If none exist, this should be stated.<br>b. List all funding sources (including grant identifier) and the role of the funder(s) in the design, analysis and reporting of the study.	a. Online form<br>b. Manuscript, page 17, lines 554-559.
