# Supplementary material for: Cardiac GR Mediates the Diurnal Rhythm in Ventricular Arrhythmia Susceptibility
Source: Circ Res. 2024 Mar 27;134(10):1306–26. doi: 10.1161/CIRCRESAHA.123.323464 (PMC11081863; doi:10.1161/CIRCRESAHA.123.323464)
Supplement: Supplementary file 2 [file res-134-1306-s002.pdf]

SUPPLEMENTAL MATERIAL

Expanded Materials and Methods

Animals

All animal procedures were approved by the University of Manchester in accordance with the UK Animals (Scientific Procedures) Act 1986 or where appropriate by the Animal Care and Use Committee at the National Institute of Environmental Health Sciences (NIEHS, USA) and by the ethical panel of University of Montpellier and by The French Ministry of Agriculture (protocol no: 2017010310594939). Experiments were performed on male C57BL/6J mice aged 8-12 weeks. Experiments were also performed on male mice aged 9-16 weeks with conditional knockout of GR in cardiomyocytes (cardioGRKO mice) that were on a C57BL/6NJ background and have been previously described.[95] Cre negative littermate mice with a floxed GR locus ($GR^{fl/fl}$) served as controls for the knockout mice. Mice were housed in a random order on shelves under a strict 12 h:12 h lightdark cycle in a temperature-controlled environment (22°C) with access to food and water *ad libitum*. Animals were housed under these conditions for at least 7 days prior to a procedure or tissue collection. All *in vivo* studies were performed by experienced staff and animals monitored carefully throughout interventions to minimise pain, suffering and distress. No adverse effects were noted and the humane endpoint of sustained changes in behaviour, surgical complications, or adverse effects resulting from administration of substances was not reached. No animals were excluded from the analysis. A protocol (including the research question, key design features and analysis plan) was prepared before the study and approved by the relevant animal facility at each participating institution.

Tissue was collected at specified time points (typically at 4 h intervals) across the 24 h cycle, with zeitgeber time (ZT) 0 denoting the start of the light period and ZT12 the start of the dark period. At the end of experiments, mice were sacrificed by cervical dislocation. Animals were randomised into ZT groups and vehicle and RU486-treated groups using a random number generator. Measurements were made in a random order and wherever possible the investigator was blinded to the time or treatment group. Blinding was performed during ATACseq analysis and in all arrhythmia inducibility studies. Comparisons were made between parameters at either (i) ZT0 *vs*. ZT12 and/or (ii) vehicle *vs*. drug or (iii) between timepoints (analysis of 24 h rhythms) in wild-type animals or in GR^{fl} *vs*. cardioGRKO animals. Unless stated otherwise the experimental unit compared is a cage of 5 biological replicates. A limitation of this study is that experiments were only performed in male mice, and it remains to be determined whether our results extend to females. Male mice were used because there are established sex differences in hypothalamic-pituitary-adrenal axis activity[96] that orchestrates glucocorticoid release, as well as in the cardiomyocyte circadian clock mechanism in the hearts of male *vs*. female mice.[97] Male mice are regarded as having a more simplified hormonal landscape due to the absence of an oestrous cycle, reducing variability in studies where the effects of hormones are considered. Furthermore, as our studies utilise RU486 that has high affinity for the progesterone receptor, there are potential welfare and ethical considerations associated with oestrous cycles and reproductive health which were circumvented using male mice.

In vivo experiments

Assessment of plasma corticosterone: To assess 24 h rhythms in plasma corticosterone, blood samples were collected at the specified timepoints within 30 s of handling the animal by tail lance, or from the chest cavity following humane culling. Plasma corticosterone was measured using a commercially available ELISA kit (K014-H1, Arbor assays).

Pharmacological GR blockade and acute GR activation*:* The GR was blocked pharmacologically by intraperitoneal (i.p) injection of (20 mg/kg) RU486 for 4 days at ZT6. Animals in the control group (Vehicle) received an equivalent volume of the diluent (70% PEG-400 in saline). In a separate study 1 mg/kg dexamethasone or an equivalent volume of saline was administered (i.p) at ZT0 to activate the GR, and hearts harvested after 15 minutes.

Biotelemetry*:* To assess the effect of GR knockout on heart rate and rhythm, biotelemetry devices were implanted as described previously.[17, 66] ECG data was continuously recorded at 2 kHz

over a 48h period using Ponemah™V6 acquisition software (Data Sciences International). Heart rate over the recording period was assessed using automatic R-wave detection in Ponemah. Detection capability was verified prior to analysis. For all other ECG parameters, automatic wave detection by Ponemah proved inadequate. In this case hour-long recordings, at ZT0-1 and ZT12-13 were imported into LabChart (v8) for determination of PR, QRS and QT intervals. T was defined as the point where the trace returned 95% of the way from the T-wave minima to the isoelectric level.[16]

Nuclei isolation and sorting

ATAC-seq was performed to investigate differential chromatin accessibility and ZT0 vs ZT12 differences in transcription factor binding motifs in left ventricular cardiomyocytes. Frozen left ventricle samples (3 independent pooled samples per replicate) were added directly to 1 ml cold lysis buffer (containing 0.32 M sucrose, 5 mM $CaCl_2$, 3 mM MgAc, 2 mM EDTA pH 8, 0.5 mM EGTA, 10 mM Tris-HCl pH 8, 1 mM DTT, 1x protease inhibitor cocktail, 80 U/ml Rnasin Plus and 0.2% Triton X-100) and quickly minced into small pieces using microdissection scissors in a petri dish on ice. In a 1 ml dounce homogeniser, 18 x loose strokes were immediately followed by 3-4 x tight strokes. To eliminate large debris, the suspension was passed over 2 layers of sterile cotton gauze prior to filtration with a 100 µM pluriStrainer (pluriSelect) and a 40 µM Flowmi filter (Sigma). Filtered suspensions were centrifuged at 1000 g for 8 min at 4°C to pellet the nuclei. The nuclei pellet was suspended in 200 µl ATAC-RSB+Tween (10 mM Tris-HCl pH 7.4, 10 mM NaCl, 3 mM $MgCl_2$, 0.1% Tween-20) and a 5 µl aliquot added in a 1:1 ratio with trypan blue for counting using a manual haemocytometer and light microscope. In the heart, antibody labelling for $PCM1^+$ (centrosome protein on the nuclei present on the nuclear surface of terminally differentiated myocytes) has been used to select cardiomyocyte nuclei using downstream fluorescence-activated nuclei sorting or magnetic bead immunoprecipitation.[29, 98] Therefore to isolate cardiomyocyte nuclei, suspensions were pelleted (1000 g for 8 min at 4°C) in ATAC-RSB-Tween and then resuspended in 500 µl staining buffer (PBS + 2.5% bovine serum albumin) containing rabbit anti-PCM1 primary antibody (1:400, Atlas Antibodies #HPA023374). Tubes were agitated on a rocker at 4°C for 1 h. Nuclei were pelleted (1000 g for 8 min at 4°C) again prior to resuspension in staining buffer containing goat anti-rabbit Cy5 secondary antibody (1:1000, Abcam #ab97077) and DAPI (1:10,000). Tubes were agitated on a rocker at 4°C for 1 h. Subsequently, nuclei were pelleted again and resuspended in ATAC-RSBTween for imaging flow cytometry or fluorescence-activated nuclei sorting. Sorting of single nuclei was performed on a BD Influx Cell Sorter (BD Biosciences) using a 100 µm diameter nozzle and 20 PSI. System alignment and drop delay settings were established using Cytometry Set Up and Tracking (CS&T) beads (BD Biosciences) and AccuDrop beads (BD Biosciences) respectively prior to sorting. Nuclear fraction samples were stained with DAPI and Cy5 before running on the cell sorter. Nuclei concentration was adjusted to run samples at 5,000 events per second and sorted nuclei were collected into individual 1.5 ml polypropylene tubes containing ATAC-RSB-Tween.

ATAC library preparation and sequencing: ATAC-seq libraries were prepared using a previously described Omni-ATAC-Seq protocol.[99] 50,000 nuclei were pelleted by centrifugation at 1000 g for 8 min at 4°C in ATAC-RSB-Tween. The supernatant was discarded ensuring not to disturb the pellet. Tn5 transposition mixtures were prepared using the Illumina Tagment DNA TDE1 Enzyme and Buffer kit (#20034197) to a final volume of 50 µl containing 25 µl 2´ TD buffer, 2.5 µl TDE1 transposase, 16.5 µl PBS, 0.5 µl 0.5% digitonin, 0.5 µl 10% Tween-20 and 5 µl nuclease-free H_2O. The transposition mixture was added to the nuclei pellet, pipetting up and down 10 ´ to mix. Reactions were incubated at 37°C for 30 min at 1000 rpm in an Eppendorf Thermomixer. Following transposition, DNA was cleaned using the Zymo DNA Clean and Concentrator-5 kit (#D4014), eluting the purified DNA fragments in a final volume of 20 µl. Following the transposase reaction, the DNA fragments were PCR amplified with combined dual indexing primers (Integrated DNA Technologies) to produce libraries for sequencing. Library pre-amplification was performed in reactions containing 20 µl DNA, 25 µl NEBNext High-Fidelity 2X PCR master mix (New England BioLabs, #M0541S) and 6.25 µl of primer mix. Thermal cycling was performed at 72°C for 5 min, 98°C for 30 sec, then 5 cycles of 98°C for 10 sec, 63°C for 30 sec and 72°C for 1 min. qPCR was used to determine the number of additional amplification cycles required for each sample in

15 µl reactions: 5 µl pre-amplified mixture, 5 µl NEBNext High-Fidelity 2XPCR master mix, 1.25 µl primer mix, 0.24 µl 25´ SYBR Green I (Invitrogen, #S7563) and 3.51 µl nuclease-free H_2O. Thermal cycling was performed on a QuantStudio 7 Flex Real-Time PCR System (Applied Biosystems): 98°C for 30 sec. then 20 cycles of 98°C for 10 sec, 63°C for 30 sec and 72°C for 1 min. Data were collected using QuantStudio Software V1.3 (Applied Biosystems). Relative fluorescence (Rn) values were exported and analysed in Microsoft Excel to determine additional PCR cycles, by determining the number of cycles required for the amplification to reach the exponential phase (1/3 of the maximumminimum Rn). Additional PCR amplification cycles were performed in a thermal cycler. Following library amplification, a double-sided fragment size selection was performed using Ampure XP beads (Beckman Coulter, #A63880) using a final 1.8´ bead buffer:sample ratio to remove primer dimers and fragments >1,000 bp. Library fragment size distribution was assessed by Agilent TapeStation 2200 using Agilent D1000 ScreenTape (#5067-5582) and reagents (#5067-5583). Libraries were sequenced on an Illumina HiSeq 4000 System. ATAC-seq data has been deposited onto ArrayExpress (https://www.ebi.ac.uk/biostudies/arrayexpress) with the accession number E-MTAB13696.

Bioinformatics and statistical analysis: Unmapped paired-reads of 76 bp were checked using a quality control pipeline consisting of FastQC v0.11.3 (http://www.bioinformatics.babraham.ac.uk/projects/fastqc/) and FastQ Screen v0.14.0 (https://www.bioinformatics.babraham.ac.uk/projects/fastq_screen/). Reads were trimmed to remove adapters and low quality bases using Trimmomatic v0.39;[100] reads were truncated at a sliding 4 bp window, starting 5', with a mean quality <Q20, and removed if the final length was less than 35 bp. Additional flags included: 'SLIDINGWINDOW:4:20 MINLEN:35. Paired-end reads were mapped to the mouse genome (UCSC mm10) using Bowtie2 v2.4.1[101] using additional parameters ('-X 2000 –very-sensitive). Samtools v1.9[102] was used to create and sort BAM files from the SAM files output from Bowtie2. Picard v2.1.0 MarkDuplicates was used to remove duplicate reads on the same strand. Reads located in blacklist regions were removed using bedtools intersect v2.27.1. Subsequently, reads were filtered to retain only concordant read pairs with a minimum quality score of 30, using samtools view. Prior to peak calling, read pairs were removed that mapped to the mitochondrial genome or unassembled contigs, using the Linus bash tool 'sed'. The Linux bash tool 'awk' was used to only extract read pairs mapping to the mitochondrial genome for quality control. Accessible chromatin peaks were identified using MACS2 v2.2.7.1[103] using additional parameters (-format BAMPE –gsize hs –keep-dup all –qvalue 0.01 –nolambda –bdg ms –SPMR –call-summits). DNA fragment coverage profiles generated by MACS2 were converted into bigWig and bigBed format using the UCSC tools bedClip, bedGraphToBigWig and bedToBigBed. The web-based Galaxy platform[104] was used to assess similarity in ATAC-seq coverage between samples. BED files containing the genomic intervals of identified peaks were intersected using bedtools intersect to identify a set of 52,510 consensus regions which were accessible in at least one sample from each group. The deepTools v3.5.1[105] function multiBigwigSummary was used to compute the average coverage score at consensus peaks for each sample, and Spearman correlation coefficients (ρ) were derived and plotted (pairwise scatterplots and heatmap in Supplemental Figure S1) using the plotCorrelation function. Differential accessibility analysis was performed using DiffBind v3.4.0 in R v4.1.2. The 'peak' input consisted of 200bp coordinates, in BED format, centred upon the summits output by MACS2. The 'read' input were the final filtered BAM files used in the MACS2 peak calling. A standard workflow was used guided by the R vignette (https://bioconductor.org/packages/release/bioc/vignettes/DiffBind/inst/doc/DiffBind.pdf). Dba.count parameters were set differently from the default (minOverlap=2, summits=FALSE).

The two closest genes were associated with each accessible region using RnaChipIntegrator v2.0.0. The gene annotation for Gencode vM23 (knownCanonical) was downloaded using the UCSC table browser. Histograms for distance from the TSS and gene ontology (GO) analysis on differentially accessible regions (FDR<0.1) were derived by uploading genomic co-ordinates into GREAT and a Benjamini-Hochberg q-value cut-off of <0.05 was applied to determine significantly enriched biological processes.[32] To determine cardiac rhythmicity in genes that annotated to differentially accessible peaks,

the publicly available Circadian Expression Profiles Data Base (CircaDb, CircaDb.org) was utilised. This database contains curated DNA microarray and RNA-seq datasets from several mouse organs, searchable by JTK_Cycle parameters. Gene lists were inputted to CircaDb with a JTK_Cycle P value < 0.05 and circadian period (0 – 28) applied to identify rhythmic genes. The JTK_Cycle LAG (phase) estimate was used to plot gene expression phase histograms for rhythmic genes. Transcription factor motif enrichment analysis was performed using SeqPos on the Cistrome platform.[36] The curated Cistrome motif database (containing motifs from Transfac, JASPAR, UniPROBE, hPDI and from ChIP-seq data) was used to search for motifs within +/- 300 bp of peak summits (default settings) associated with chromatin regions more accessible at ZT0 and ZT12. SeqPos calculates the distance of motifs from peak summits to find the most enriched motifs near peak summits. SeqPos returned clusters of enriched DNA binding motifs stratified by Z-score and P values. Publicly available ChIP-seq were downloaded from the NCBI GEO for adult mouse heart active promoter and histone modification H3K27ac (GSE124008 from Akerberg *et al.*[32]) and neonatal rat ventricular myocyte GR (GSE114767 from Severinova *et al.*[37]) binding sites. To determine overlap between H3K27ac ChIP-seq peaks and accessible chromatin regions determined in this study, 'Bedtools Intersect' on the Galaxy web platform was used to intersect the genomic intervals, returning overlapping genomic intervals between datasets only. For GR ChiP-seq data, genes with GR binding sites (demonstrating >2-fold GR ChiP enrichment *vs*. control following dexamethasone treatment) were intersected with the list of genes that showed differential chromatin accessibility and significant motif enrichment for the GR as determined in this study. GO analysis of the overlapping genes was performed using DAVID with default parameters.[106, 107]

Immunolabelling and image analysis

Immunolabelling studies were performed to analyse time-of-day dependent variation in the GR nuclear labelling and to estimate 24h rhythms in the membrane protein expression levels of GR targets-of-interest. Immunofluorescence was performed on formalin-fixed paraffin embedded sections of left ventricular biopsies. 10 µm sections underwent dewaxing with histoclear, dehydration in graded ethanol, antigen retrieval in pre-heated sodium citrate buffer (pH = 6.0) at 95 ^{0}C for 20 minutes followed by permeabilization with TritonX-100 (0.05% in PBS tween 0.05%) for 10 minutes at room temperature and treatment with 5% BSA diluted in PBS tween (0.05%) for 1 hour at room temperature. The following antibodies were used: anti-GR primary antibody (#3660S, Cell Signaling Technology), anti-SCN5A (#ASC-005, Alomone Labs), anti-Cx43 antibody (rabbit polyclonal, #ACC201, 1:100, Alomone labs) and Lectin from triticum vulgaris FITC conjugate also known as wheat germ agglutinin (WGA) (#L4895, Sigma-Aldrich) for cell membrane labelling. Antibodies were selected based on their demonstrated specificity for mouse tissue verified by immunolabelling studies in previous publications.[e.g.108-110] To validate that staining is produced from detection of the antigen by primary antibody, we utilized a control group in which the primary antibody was omitted (secondary antibody-only control). In all cases, no non-specific staining was observed in this control group. Primary antibodies were incubated overnight at 4°C and secondary antibodies (alexa Fluor 594 goat anti-rabbit IgG, #A32740, Invitrogen) were applied for 3 h at room temperature. For each antibody, staining and analysis of sections from different time points was performed simultaneously. Slides were washed in PBS tween and ProLong Gold antifade reagent containing DAPI was applied. Slides were imaged at 63X with a Leica DM5000 B epifluorescent microscope. Images mainly containing cardiomyocytes in cross section per animal and time point (~64 cells per image) were analysed for GR labelling intensity and co-localisation with the DAPI signal. Images were analysed using FIJI Image J software. Thresholding and 'Analyse Particles' function was used to identify $DAPI^+$ and GR^+ nuclei within cardiomyocyte boundaries defined by WGA labelling. Threshold was estimated as a 90th percentile of a positive GR signal at ZT0. Number of GR^+DAPI^+ cells relative to total number of $DAPI^+$ cardiomyocytes was assessed. Fluorescent intensity of GR^+ labelling (that colocalised with the $DAPI^+$ signal) was calculated using the Mean Intensity function. Values were averaged per animal. For SCN5A and Cx43 labelling, threshold was estimated as a 90th percentile of a positive signal at ZT0. For Cx43, positive signal area normalized to the tissue area and fluorescent labelling intensity were calculated. For SCN5A, cardiomyocytes were identified using DAPI and WGA

staining and manually added to the 'ROI manager'. Cells with SCN5A$^+$ signal on the cell membrane were further normalised to the total number of DAPI$^+$WGA$^+$ cardiomyocytes. 1,227 ventricular cardiomyocytes in 20 sections from 4 mice (5 sections per mouse) at ZT0 were compared to 985 ventricular cardiomyocytes analysed in in 20 sections from 4 mice (5 sections per mouse) at ZT12. In all cases, images that best summarised the group effect and that were most similar to other images in the group were selected as representatives.

ChIP-qPCR

ChIP-qPCR was performed to investigate direct GR-DNA binding for targets of interest. Samples were sent to Active Motif (Carlsbad, CA) for ChIP-qPCR. Active Motif prepared the chromatin, performed the ChIP reactions and the qPCR analysis. Mouse heart tissue was submersed in PBS + 1% formaldehyde, cut into small pieces, and incubated at room temperature for 15 minutes. Fixation was stopped by the addition of 0.125 M glycine (final). The tissue pieces were then treated with a TissueTearer and finally spun down and washed 2x in PBS. Chromatin was isolated by adding lysis buffer, followed by disruption with a Dounce homogenizer. Lysates were sonicated and the DNA sheared to an average length of 300-500 bp with Active Motif's EpiShear probe sonicator (# 53051). Genomic DNA (Input) was prepared by treating aliquots of chromatin with RNase, proteinase K and heat for de-crosslinking, followed by SPRI beads clean up (Beckman Coulter) and quantitation by Clariostar (BMG Labtech). Extrapolation to the original chromatin volume allowed determination of the total chromatin yield. An aliquot of chromatin (15 µg) was precleared with protein G agarose beads (Invitrogen). Genomic DNA regions of interest were isolated using 20 µl (4 µg) of anti-GR antibody (#sc-8992, Santa Cruz Biotechnologies). Complexes were washed, eluted from the beads with SDS buffer, and subjected to RNase and proteinase K treatment. Crosslinks were reversed by incubation overnight at 65℃, and ChIP DNA was purified by phenol-chloroform extraction and ethanol precipitation. Quantitative PCR (qPCR) reactions were carried out in triplicate for specific genomic regions using SYBR Green Supermix (#170-8882, Bio-Rad) on a CFX Connect™ Real Time PCR system. The resulting signals were normalized for primer efficiency by carrying out qPCR for each primer pair with input DNA isolated and pooled from all samples. A negative control primer set (Active Motif, #71011) was also used.

Primer sequences were as follows:

Gene name	Primer sequence
m Per2_+22.8KA	GCTGCCTTTGCCTCTGTTAG
m Per2_+22.8KB	ACCTGTTGGGATGCTTGAATG
m Scn5a_ +78.6KA	GAGAGGCATCACCAGGACA
m Scn5a_ +78.6KB	GCGGGCAAGTCAAGTATTAT
m Scn5a_-12KA	GCCCAAGGAGAGTGGTGTAG
m Scn5a_12KB	CGGGACTTGAATAGCAAGAAAC
m Scn5a_ +4.8KA	CATGCCTGTGAGTAGGTAGAACAC
m Scn5a_ +4.8KB	CTGTCCCACATTAAGGCTG
m Kcnh2_+13.7KA	CCGTGTCGCTAACACCACTC
m Kcnh2_+13.7KB	CCCAGAACCCTGTATAGCTC

m Kcnh2_+1.2KA	TCTGCCCCTGCCTACCTAAC
m Kcnh2_+1.2KB	TGACATTCCTTGCCTGTGAC
m Klf15_+8.7KA	GGTCTAACCATCTGGGAAGT
m Klf15_+8.7KB	GTCCAGCTCCTCCAACAGTG

qPCR

qPCR was performed to assess 24h rhythms in transcripts of interest according to previously described methods.[111] Briefly, total RNA was extracted from frozen left ventricular samples using an RNeasy Mini kit (Qiagen, #74104) and cDNA generated using a High-Capacity RNA-to-cDNA™ kit (Applied Biosystems, #4387406). All primers were purchased from Qiagen. Tbp and Ipo8 were used for normalisation.

Western blotting

Frozen left ventricular free wall biopsies were homogenised in lysis buffer (RLT, Qiagen) with β255 mercaptoethanol. Homogenate was then added to an equal volume of ethanol and centrifuged (13,000 rpm, 5 min). The supernatant was added to RNEeasy micro columns (Qiagen) and centrifuged (1,000 rpm, 15 secs) following which flow-through was added to acetone at a ratio of 1:4 (lysate:acetone). Protein suspended in acetone was then centrifuged at 4°C (13,000 rpm, 5 min) and the supernatant was discarded. The remaining pellet was washed in 100% ethanol and centrifuged at 4°C (13,000 rpm, 5 min). Pellets were then air-dried and solubilised in 2x Laemelli buffer. Protein lysate was heated to 37°C for 10 min. Then approximately 10 µg was loaded on 4-20% MiniPROTEAN® TGX™ Precast Protein Gels (Bio-Rad, 561094), with a Precision Plus Protein™ Streptagged recombinant Unstained Protein Standard (Bio-Rad, 1610363). Gel-electrophoresis occurred in 1x Tris/Glycine/SDS buffer (Bio-Rad, 1610772) at 110V for 70 min at room temperature. Gels were then imaged using a ChemiDoc MP system (Bio-Rad) for 2.5 min. Protein from gels was transferred onto 0.2 µm PVDF membranes using a Trans-Blot Turbo RTA Transfer Kit (Bio-Rad) using a semi-dry transfer in a TransBlot Turbo (Bio-Rad) at 1A, 25V for 30 min. Membranes were then imaged for total protein transfer using a ChemicDoc MP System for 0.468s. Membranes were washed in 1x TBST (25mM tris, 0.15mM NaCl, 0.1% tween-20, pH 7.5) for 5 min and then blocked in 5% milk in TBST (1 hour). Membranes were then probed with primary antibodies specific to either SCN5A or KCNH2 at 4°C overnight (SCN5A, #493-51, Abcam; KCNH2, #OSP00150W). Following this, membranes were washed in TBST for 15 min. Membranes were probed the anti-rabbit IgG HRP-linked secondary antibody (Cell Signaling Technologies, #7074; 1:3333 dilution) in 5% milk for 1 hour. Membranes were incubated with a StrepTactin-HRP conjugate (Bio-Rad, 1610381; 1:5000) for 30 min then washed in 1x TBST for 15 min and incubated in a Clarity Western ECL Substrate (Bio-Rad, 170-5061) for 5 min. Western blot membranes were imaged using a ChemiDoc MP and ImageStudio (Bio-Rad) was used for quantification. Chemiluminescent signal intensity was normalised to total protein, calculated and volume-adjusted using Image Lab by selection of equivalent lane segments across the blot on the total protein image. Blots were run in duplicate and original and uncropped gels and blots are attached in Online Supplemental Material.

Arrhythmia inducibility

Time-of-day dependent propensity to ventricular tachyarrhythmia, and its modulation by pharmacological and genetic block of the GR was assessed in Langendorff-perfused hearts. The heart was mounted on a Langendorff column and retrogradely perfused with oxygenated Tyrode solution at 37°C. A pseudo-ECG was recorded using extracellular electrodes and MappingLab software. After a 15

min stabilisation period, hearts were subjected to programmed electrical stimulation as described previously, including an S1 train consisting of 20 pulses at 98-ms cycle length immediately followed by an S2–S10 train of extra stimuli ranging from 58 ms down to 8ms, decreasing in 10ms intervals.[63] Each segment of stimulation was separated by a 3 s gap, with a total of 6 segments. VA's were defined as rapid and chaotic activation patterns exceeding 3 s occurring at the end of the stimulation period. If arrhythmia was not induced, the protocol was repeated in the presence of 0.2 μM isoprenaline.

RNAseq

Total RNA was isolated from the left ventricle free wall using Qiagen RNeasy Mini kit to assess transcriptomic changes in $GR^{fl/fl}$ *vs.* cardioGRKO animals and modification of ZT0/ZT12 differences between groups. A TruSeq RNA kit (Illumina, San Diego, CA) was utilized to prepare the poly(A)enriched RNA-seq libraries that were sequenced on the Illumina NovaSeq 6000 in a 75-base pairedend mode according to the manufacturer's protocol. The quality of raw RNA-seq sequences for each sample was assessed with FastQC (v 0.11.5). Reads (average of ~51 million per sample) were mapped to the University of California Santa Cruz (UCSC) mm10 reference genome using STAR (V 2.5.1). The quantification results from "featureCount (subread, Version 1.5.1)" were then analyzed with the Bioconductor package DESeq2 (v 1.38.3), which fits a negative binomial distribution to estimate technical and biological variability. A factorial design was implemented to capture the GR knockout effect and time effect, and differentially expressed genes were obtained using a BenjaminiHochberg adjusted P value (q-value)<0.05. For the Principal Component Analysis (PCA), the gene expression data matrix was preprocessed to filter out genes in which less than 10 counts were recorded in 3 or more samples. Variance stabilizing transformation was then applied to the count matrix blind to the experimental design, and default number of sampling was used and a robust gamma-family general linear model was chosen for dispersion estimation. The RNA-seq data has been deposited in the Gene Expression Omnibus with the accession number GSE236548.

Weighted gene co-expression network analysis: Weighted gene co-expression network analysis (WGCNA) was performed using a WGCNA package in R.[76] The input data comprised RNAseq-determined gene expression levels for 7644 genes (maximal number dictated by available computing power) from $GR^{fl/fl}$ and cardioGRKO mice at ZT12 ($GR^{fl/fl}$ *vs.* cardioGRKO (FDR <0.44; P<0.23). Input counts were transformed into $\log_2$ and normalized based on quantiles. Spearman correlations between each gene pair were calculated to build an adjacency matrix for the $GR^{fl/fl}$ and cardioGRKO groups at ZT12 conditions respectively. A soft-threshold was chosen basing on kmeans calculations. Next, the topological overlap measure (TOM) and corresponding dissimilarity (1-TOM) was calculated using an adjacency matrix. 1-TOM was used as a distance for gene hierarchical cluster, following which DynamicTree Cut algorithm was performed for cluster identification (**Supplemental Figure S12A**). As a result, genes were grouped in 14 clusters and a comparative heatmap describing correlation patterns of gene clusters in $GR^{fl/fl}$ and cardioGRKO conditions was plotted (**Supplemental Figure S12B**). A permutation test was used to identify modules with significant differences in co-expression patterns between $GR^{fl/fl}$ and cardioGRKO groups. KEGG over-representation analysis was performed using WebGestalt. A protein-protein interaction (PPI) network modules showing differential co-expression (modules 1 and 3) was constructed in STRING[77] (n = 1157 genes) and yielded a significantly enriched network with strongly intersecting nodes (PPI enrichment P<7.77 x10^{-16}). A sub-PPI network showing components from significantly enriched KEGG terms relevant to cardiomyocyte biology was constructed, and also resulted in a significantly enriched network (P<1.0x10^{-16}). This network was visualised and topologically characterised in Cytoscape and is given in **Figure 8C.**

Patch clamp electrophysiology

ZT12 vs. ZT0 changes in ionic currents carried by GR target ion channels was assessed in isolated ventricular cardiomyocytes by the whole cell patch clamp technique.

Isolation of adult ventricular cardiomyocytes*:* Hearts were isolated from wild-type C57Bl/6J adult male mice at ZT0 and ZT12. Mice were first anaesthetized by intraperitoneal injection of a mixture

containing ketamine (100 mg/kg) and xylazine (10 mg/kg), followed by injection of sodium pentobarbital (45 mg/kg; Ceva Santé Animale, France). Finally, intraperitoneal injection of heparin (7500 IU/kg; Sanofi Aventis, France) was administered to avoid formation of intracardiac blood clots. Thoracotomy was performed and beating hearts were quickly excised into Tyrode solution containing (in mM): 140 NaCl, 5.4 KCl, 1.8 $CaCl_2$, 1 $MgCl_2$, 5 HEPES-NaOH, and 5.5 dglucose (adjusted to pH 7.4 with NaOH). For isolation of ventricular cardiomyocytes excised hearts were quickly mounted on a Langendorff apparatus. Hearts were perfused with a Ca^{2+}-free Tyrode solution for 4 minutes followed by an enzymatic solution containing Liberase TM (0.2 mg/ml) and Trypsin (0.14 mg/ml) for 3.5 min. Hearts were removed from the apparatus and harvested in "stop solution" (10 mM BDM, 5.5 mM glucose, 12.5 µM $CaCl_2$ and 5% Fetal Calf Serum) to block enzymatic digestion. Atria were then removed quickly, and the ventricles were chopped into small pieces (~1 mm^3) using dissection forceps and dissociated by gentle pipetting using a flame-forged Pasteur pipette. Cell suspension was then filtered using a medical gauze and myocytes gravity settled for 20 min. To restore Ca^{2+} concentration to physiological levels, a "Ca^{2+} reintroduction" buffer containing (mM): NaCl, 140; KCl, 5.4; $MgCl_2$, 1; $CaCl_2$, 1.8; Hepes, 5 and glucose, 5.5 (pH adjusted to 7.4 with NaOH) was added progressively.

Patch clamp recording of ventricular myocytes: For electrophysiological recordings, aliquots of ventricular cardiomyocytes were harvested in 3.5 cm Petri dishes and mounted on the stage of an inverted microscope. Myocytes were then continuously perfused with normal Tyrode's solution. The recording temperature was set to 36°C except for I_{Na} recordings performed at room temperature. The whole-cell variation of the patch-clamp technique was used to record ionic currents, by employing an Axopatch 700A or 700B (Axon Instruments Inc., Foster USA) patch clamp amplifier. Recording electrodes were fabricated from borosilicate glass, using DMZUniversalElectrode-Puller (Zeitz-Instruments Vertriebs GmbH, Martinsried, Germany). I_{Na} was recorded at room temperature (22 °C) using modified low Na^+ Tyrode's solution containing (in mM): NaCl, 10; tetraethylammonium chloride (TEA-Cl), 130; KCl, 5.4; MgCl2, 1; $CaCl_2$, 1.8; Hepes, 5 and glucose, 5.5 (pH adjusted to 7.4 with NaOH). The dihydropyridine I_{CaL} blocker nifedipine (5 µM) was added to external solution to block $Ca_v1.2$ mediated I_{CaL}. Recording of I_{Na} was performed by using intracellular pipette solution containing (in mM): CsOH 125, TEA-Cl 20, $CaCl_2$ 1.2, Mg-ATP 5, Li2-GTP 0.1, EGTA 5 and HEPES 10 (pH adjusted to 7.2 with aspartic acid). I_{Kr} and I_{to} were recorded under perfusion of normal Tyrode's solution. Patch pipettes were filled with an intracellular solution containing (in mM): K-Asp 80, KCl 50, MgCl2 1, HEPES 5, $CaCl_2$ 2, EGTA 5,Na-ATP 3, (pH adjusted to 7.2 with KOH). Recording patch-clamp electrodes had a resistance of ~1.5 MΩ. Seal resistances were in the range of 2–5 GΩ. Cell capacitance and series resistance were compensated up to 90%. All electrophysiological data were recorded using pCLAMP 8 (Molecular Devices, Sunnyvale, CA, USA). Data were analysed off-line using Clampfit 9.2 (Molecular Devices, Sunnyvale, CA, USA).

Biophysically-detailed computer modelling

The impact of 24h variation in GR-target ion channels and connexin 43 on the ventricular action potential and propensity to reentry was assessed *in silico*. The study of the mouse ventricular action potential was carried out using the mouse ventricular action potential model from Morotti *et al.*[56] The modules of the cell model concerning ion channels, Ca2+ handling and excitation- contraction coupling were implemented, while the modules considering Ca^{2+}/calmodulin-dependent protein kinase II (CaMKII) and protein kinase A signaling pathways were treated as static biological processes with equilibrium states.

Single cell simulations: In simulations, the basal Morotti *et al.*[56] model for murine ventricular cells was treated as the control model that simulates action potentials for ZT0. To simulate action potentials for ZT12 and the presence of glucocorticoid (GCs) at the ZT12, the parameters of ionic channel conductances and Ca^{2+} handling were scaled by the ratio of the measured levels at ZT12 and ZT0 (given in Supplemental Tables 1 and 2) in the single-cell model of Morotti *et al.*, in which the change in the transmembrane action potential is given by:

$$\frac{dV_m}{dt} = -\frac{I_{ion,tot} + I_{st}}{C_m}, \quad (1)$$

where V_m is the transmembrane potential, $I_{ion,tot}$ the total ionic current, (I_{st} the stimulus current, and C_m the membrane capacitance.

To solve Equation (1) of the action potential (AP) model, the forward explicit Euler method was employed, using a time step 0.0001 ms, which is sufficient small to produce stable numerical solution of Equation (1). To handle the ordinary differential equations associated with the gating variables of ion channels, we utilized the Rush-Larsen method.[112] To quantitatively assessed the functional impact of the altered ionic channel conductance due to different ZT and pharmacological effects during ZT12, we evaluated their effects on the characteristics of APs, such as the amplitude of action potentials, the maximum upstroke velocity during depolarization and the action potential duration at 90% repolarization level. In simulations, action potentials were evoked by a series of external stimuli. In order to measure the restitution properties of simulated action potentials, the S1-S2 stimulus protocol was used, with S1 being 100 external conditioning stimuli to stabilise the model, and the S2 being an additional external stimulus after a variable time delays to the last of the S1 stimuli.

One dimensional cell string simulations. The diffusion partial differential equation was used for simulating the conduction of ventricular excitation waves in an one-dimensional (1D) model of cardiac tissue strand, which takes the form:

$$\frac{\partial V_m}{\partial t} = \nabla \cdot \boldsymbol{D} \nabla V_m - \frac{I_{ion,tot+I_{st}}}{C_m}, \quad (2)$$

where **D** is the diffusion tensor and 0 is the spatial gradient operator.

In numerical simulations, the explicit finite difference method (FDM) was employed to solve Equation (2) with non-flux boundary conditions at the edges, in order to obtain numerical solutions of AP across the tissue.[113]
In simulations, the 1D strand model consisted of 100 isotropic myocardial cells, arranged in a linear configuration with a spacing of 0.15 mm between each cell. The diffusion parameter **D** is a scalar coefficient in the 1D model. For myocardial tissue at the ZT0 condition, a value of 0.046 $mm^2\ ms^{-1}$ was used for D, which produced a conduction velocity of 33.7 cm/s at a stimulation cycle length of 200 ms, which is within the range of experimental data observed in mouse ventricle.[114] For simulation the ZT12 condition, D was decreased based on the experimental data of changes in connexin, which reduced the conduction velocity to 21.1 cm/s. To quantify the temporal susceptibility of ventricular tissue in the 1D strand model, we measured vulnerability window (VW) by using the standard S1-S2 stimulus protocol. The S1 stimulus was applied to one end of the 1D strand to evoke an excitation wave propagating from the stimulus site to the other end of the tissue strand. At the middle of the tissue strand, after a time delay after the S1-evoked excitation wavefront reached, the S2 stimulus was applied. Depending on the time delay (or time interval (STI) between the S1 and S2), the S2evoked excitation wave may either fail to propagate if the STI is small, or propagate bi-directionally if the STI is large. With the STI being in a time window, the S2-evoked excitation wave would propagate unidirectionally. As such unidirectional conduction wave forms a substrate of reentrant excitation wave in 2D or 3D cardiac tissue that underlies cardiac arrhythmias, the measured time window for unidirectional conduction presents the vulnerable window (VW) of cardiac tissue,[115, 116] characterize the susceptibility of the tissue to arrhythmogenesis. In simulation to measure VW, 10 S1 stimuli were used to evoke the conditional excitation waves, which

were followed by the S2 stimulus applied to 10-nodes at the middle region of the 1D strand tissue model. Both the S1 and S2 stimuli had fixed stimulus strength of 9.5 pA/pF and duration of 5 ms.

Model of human ventricular cells: The ten Tusscher *et al.*[68] model for action potentials of human ventricular cells was employed. Action potentials were elicited in endocardial myocytes through a series of external stimuli with a strength of -20 pA/pF and a duration of 2.0 ms during simulations. The simulations were conducted with a basic cycle length set at 1000 ms. To ensure a stable numerical solution, the model was stabilized by applying 100 external S1 stimuli. Subsequently, an external S2 stimulus was applied after an S1-S2 interval of 1000 ms to evoke the action potential. Diurnal modulation and impact of GR block were simulated in the same way as described above for the mouse action potential using experimental data given in Table S2.

Statistical analysis

Animal numbers were estimated *a priori* and determined from power calculations with 80% power and a 95% confidence interval using standard deviation values from our previously published work on the diurnal rhythm in cardiac electrophysiological and transcriptional parameters in mice.[17,66,117,118] Unless stated otherwise data were analysed in GraphPad Prism 9 (GraphPad Software, Inc.). Statistical analysis of ATACseq and RNAseq datasets are detailed in the relevant sections and determination of 24 h rhythms using JTK cycle are detailed below. Differences in arrhythmia inducibility between groups was tested using a χ^2 test. For all other experiments, residual diagnostics were examined and a Shapiro-Wilk test for normal distribution applied to confirm that the assumptions for the analysis to be valid were applicable. Where data were not normally distributed and/or the number of observations was <10 a non-parametric test (Mann-Whitney test or KruskalWallis test) was used. If data were normally distributed an unpaired Students *t* test (two sided) was used. To compare multiple normally distributed groups, an ANOVA (one- or two-way) was used. Tukey's or Sidak's test was applied for multiple testing correction, automated by the software package used. P≤0.05 was regarded as significant and significant P values are given in Figures. means±SEM are given in dot plots.

Exceptions: For patch clamp experiments, we report statistical analysis based on a mixed effects linear model where the time of day (ZT0 or ZT12) and voltage were specified as fixed effects and myocytes specified as random effects. For SCN5a immunolabelling experiments we compared the percentage of cardiomyocytes with SCN5A+ membrane labelling at ZT0 vs. ZT12 using a nested t test. Examination of residual diagnostics indicated that the assumptions for a nested t test were valid. For ECG data recorded by telemetry, ECG parameters were assumed to be normally distributed as determined by our previously published observations in wild-type mice[17,66] and a mixed effects linear model was applied to assess statistical differences where the time of day (ZT0 or ZT12) and ECG parameter of interest were specified as fixed effects and animals specified as random effects.

Analysis of 24h rhythmicity: Rhythmicity in heart rate derived from biotelemetry data was assessed by cosinor analysis using Cosinor2 in R. Briefly, 24h a best-fit cosine wave was applied to heart rate data using a nonlinear regression model as previously described.[67,119] Cosine wave characteristics including MESOR - an estimation of the mean statistic, acrophase - the Zeitgeber time at the cycle's peak, and amplitude -, the difference between the peak/trough and mean statistic, were determined. A circadian (24h) rhythm was considered to be present if a cosine function within a 24-hour period could be fitted with a significance of P<0.05 by least-squares analysis. The non-parametric algorithm JTK_Cycle[35] was applied in R using DiscoRhythm to determine rhythmic cycling in all other datasets where multiple time points have been assessed. Parameters were defined as rhythmic if they which oscillated with a 24 h period against a null hypothesis of amplitude ≤ 0 (P<0.05, Bonferroni adjusted for multiple testing).

Selection of representative images: Representative images were selected on the basis that they were most similar to others in the respective group.

SUPPLEMENTAL TABLES

Table S1. Circadian rhythm in ventricular ion channels and ionic currents.

Conductance	Ionic current	Gene and common names	Source	Species	Time points	Technique	Measured	Sleep period	Active period
g_{Na}	I_{Na}	Scn5a (Nav1.5)	This study	mouse	ZT0 and ZT12	qPCR	mRNA	100%	150%
				mouse	ZT0 and ZT12	Patch clamp	Ionic current	100%	57%
$g_{Ca,L}$	$I_{Ca,L}$	Cacna1c (Cav1.2)	Anderson et al.[66]	mouse	ZT0 and ZT12	qPCR	mRNA	100%	116%
			Wang et al.[120]	mouse	ZT4 and ZT14	qPCR	mRNA	100%	107%
			Collins and Rodrigo[121]	rat	ZT3 and ZT15	qPCR	mRNA	100%	110%
			Collins and Rodrigo[121]	rat	ZT3 and ZT15	Patch clamp	Ionic current	100%	132%
			Chen et al.[13]	guineapig	ZT3 and ZT15	Patch clamp	Ionic current	100%	141%
g_{to}	I_{to}	Kcnd2 (Kv4.2)	Anderson et al.[66]	mouse	ZT0 and ZT12	qPCR	mRNA	100%	135%
			Schroder et al.[16]	mouse	ZT0 and ZT12; constant darkness; data from fitted curve	qPCR	mRNA	100%	152%
			Tong et al.[122]	mouse	ZT0 and ZT12	qPCR	mRNA	100%	70%
			Wang et al.[120]	mouse	ZT4 and ZT14	qPCR	mRNA	100%	113%
			Yamashita et al.[15]	rat	ZT6 and ZT18	RNase protection assay	mRNA	100%	41%
			This study	mouse	ZT0 and ZT12	Patch clamp	Ionic current	100%	128%
			Yamashita et al.[15]	rat	ZT6 and ZT18	Patch clamp	Ionic current	100%	70%
$g_{K,ur}$	$I_{K,ur}$	Kcna5 (Kv1.5)	Anderson et al.[66]	mouse	ZT0 and ZT12	qPCR	mRNA	100%	157%
			Tong et al.[122]	mouse	ZT0 and ZT12	qPCR	mRNA	100%	161%
			Wang et al.[120]	mouse	ZT4 and ZT14	qPCR	mRNA	100%	137%*
			Yamashita et al.[15]	rat	ZT6 and ZT18	RNase protection assay	mRNA	100%	153%
			This study	mouse	ZT0 and ZT12	Patch clamp	Ionic current	100%	100%
			Yamashita et al.[15]	rat	ZT6 and ZT18	Patch clamp	Ionic current	100%	143%
$g_{K,r}$	$I_{K,r}$	Kcnh2 (ERG)	Anderson et al.[66]	mouse	ZT0 and ZT12	qPCR	mRNA	100%	171%
			This study	mouse	ZT0 and ZT12	qPCR	mRNA	100%	156%
			Schroder et al.[16]	mouse	ZT0 and ZT12; constant darkness; data from fitted curve	qPCR	mRNA	100%	188%

			Schroder et al. [16]	rat	ZT0 and ZT12; constant darkness; data from fitted curve	qPCR	mRNA	100%	241%
			This study	mouse	ZT0 and ZT12	Patch clamp; current at end of +50 mV pulse	Ionic current	100%	252%
			This study	mouse	ZT0 and ZT12	Patch clamp; tail current	Ionic current	100%	363%
						after +20 mV pulse			
$g_{K,ss}$	Steadystate K^+ current	Not known	This study	mouse	ZT0 and ZT12	Patch clamp	Ionic current	100%	Start of pulse, 89%; end of pulse, 76%
SR Ca^{2+} uptake		Atp2a2 (Serca2a)	Anderson et al.[66]	mouse	ZT0 and ZT12	qPCR	mRNA	100%	95%
			Collins and Rodrigo[121]	rat	ZT3 and ZT15	qPCR	mRNA	100%	89%
			Wang et al.[120]	mouse	ZT4 and ZT14	Western blot	protein	100%	~100%^{ns}
SR Ca^{2+} release		Ryr2 (RyR2)	Anderson et al.[66]	mouse	ZT6 and ZT18	qPCR	mRNA	100%	72%
			Collins and Rodrigo[121]	rat	ZT3 and ZT15	qPCR	mRNA	100%	88%
			Wang et al.[120]	mouse	ZT4 and ZT14	Western blot	protein	100%	~100%^{ns}
Na^+-Ca^{2+} exchange current	I_{NaCa}	Slc8a1 (NCX1)	Anderson et al.[66]	mouse	ZT6 and ZT18	qPCR	mRNA	100%	69%
			Wang et al.[120]	mouse	ZT4 and ZT14	qPCR	mRNA	100%	96%$_{ns}$
			Collins and Rodrigo[121]	rat	ZT3 and ZT15	qPCR	mRNA	100%	78%
g_J		Gja1 (Cx43)	Anderson et al.[66]	mouse	ZT0 and ZT12	qPCR	mRNA	100%	69%
			Tong et al. [18]	mouse	ZT0 and ZT12	qPCR	mRNA	100%	54%
			This study	mouse	ZT0 and ZT12	qPCR	protein	100%	40%
		Gja1 (Cx40)	Tong et al. [18]	mouse	ZT6 and ZT18	qPCR	mRNA	100%	155%

Table S2. Ionic conductances used in the computation of ventricular electrical activity at ZT0 and ZT12 and in ZT12 following GR knockout.

Conductance	Scaling (ZT12/ZT0)	Effect of GR knockout or block (ZT12/ZT0)
g_{Na}	0.57	1 (loss of diurnal variation in *Scn5a,* Figures 5D & 7E)
$g_{Ca,L}$	1.32	1.32
g_{to}	1.28	1
$g_{K,ur}$	1	1
$g_{K,r}$	3.63	1 (loss of diurnal variation in *Kcnh2* Figures 5D & 7E)
$g_{K,ss}$	1	1
**Na^+-Ca^{2+} exchanger**	0.69	0.69
**Electrical coupling**	0.69	1 (loss of diurnal variation in *Gja1* Figure 7E)

Table S3. Computed mouse ventricular action potential properties at a basic stimulus interval of 150 ms at ZT 0, ZT 12 and ZT 12 following GR knockout.

	ZT0	ZT12	ZT12 (GR knockout)
**APD_{90} (ms)**	29.2	26.0	28.9
**dV/dt_{max} (V/s)**	269.2	170.6	268.1
**Action potential amplitude (mV)**	115.4	101.2	114.3

Table S4. Comparison of computed and experimentally estimated day-night differences in the mouse ventricular action potential duration.

Measurement	Source	Action potential duration at ZT 0 (ms)	Action potential duration at ZT 12 (ms)	Action potential duration - ZT12/ZT0 (%)
Computed action potential duration at 90% repolarization	This study (Figure 3I)	29.2	26.0	89
QT interval (surrogate of action potential duration)	This study (Figure 5H)	45	35	77
QT interval (surrogate of action potential duration)	Schroder *et al.*[12]	45	40	89
QT interval (surrogate of action potential duration)	Gottlieb *et al.* (2016)[123]	54	48	89
QJ interval (surrogate of action potential duration)	D'Souza *et al.*[17]	17	14.5	85

SUPPLEMENTAL FIGURES

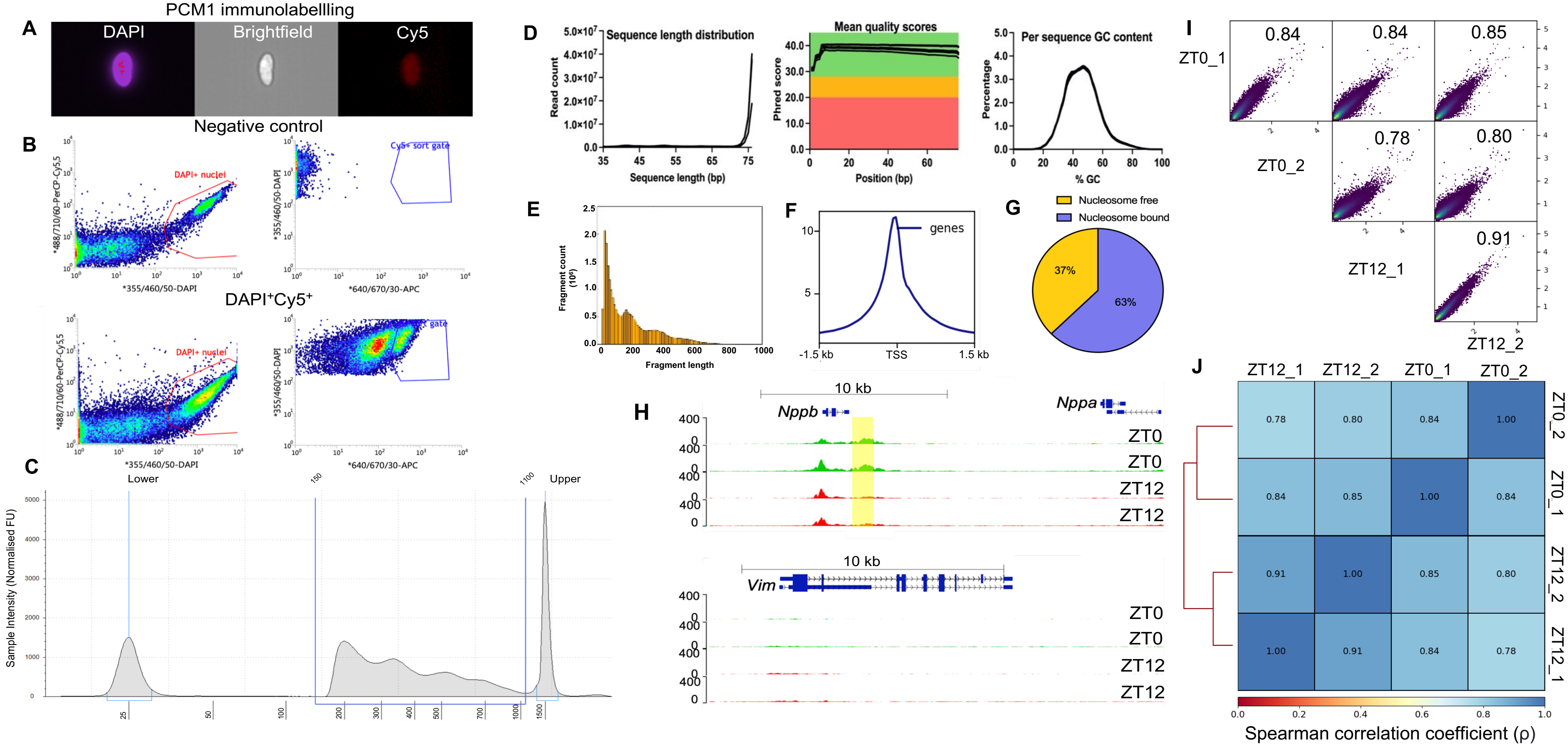

Supplemental Figure S1: Isolation of cardiomyocyte nuclei and ATACseq quality control. A, Single left ventricular cardiomyocyte nucleus in DAPI, brightfield (BF) and Cy5 channels imaged on an ImageStream Mk-II. Cy5 indicates PCM1$^+$ immunolabelling. Scale bar = 7 µm **B**, Flow cytometry dot plots demonstrating the gating strategy for sorting of PCM1$^+$ nuclei (gate demarcated in red), using DAPI stained control left ventricular nuclei to determine the Cy5$^+$ sort gate (blue). **C**, Signal intensity plots from TapeStation fragment size analysis of ATAC-seq libraries. Upper and lower markers were assigned as analysis cut-off points. **D**, FastQC metrics for sequence fragment length distribution, mean quality scores and GC content of ATAC-seq libraries. **E**, Representative fragment length distribution plots for ATAC-seq libraries. **F**, Coverage of nucleosome-free fragments around the transcription start site (TSS) of all genes in representative libraries. FPKM = fragments per kilobase million. **G,** Proportion of nucleosome-free (<100 bp) and nucleosome-bound (>100 bp) fragments in ATAC-seq libraries. **H**, Genome tracks showing ATAC-seq coverage around *Nppb, Nppa* and *Vim* gene loci, created using pyGenomeTracks. ZT0 biological replicates are shown in green and ZT12 shown in red. Yellow area indicates differentially accessible region. Reference gene transcripts are shown above the tracks with blue bars indicating exons. **I**, Density scatterplots displaying correlation of ATAC-seq data between samples. Each dot represents an individual peak in the consensus accessible sites with viridis color scale (yellow/green - high,

purple - low) indicating density. Spearman correlation coefficient (ρ) value is shown at the top of each plot. Values on X and Y axes denote normalised coverage scores. **J**, Heatmap of Spearman correlation coefficients (ρ) comparing ATAC-seq coverage at 52,510 consensus accessible sites.

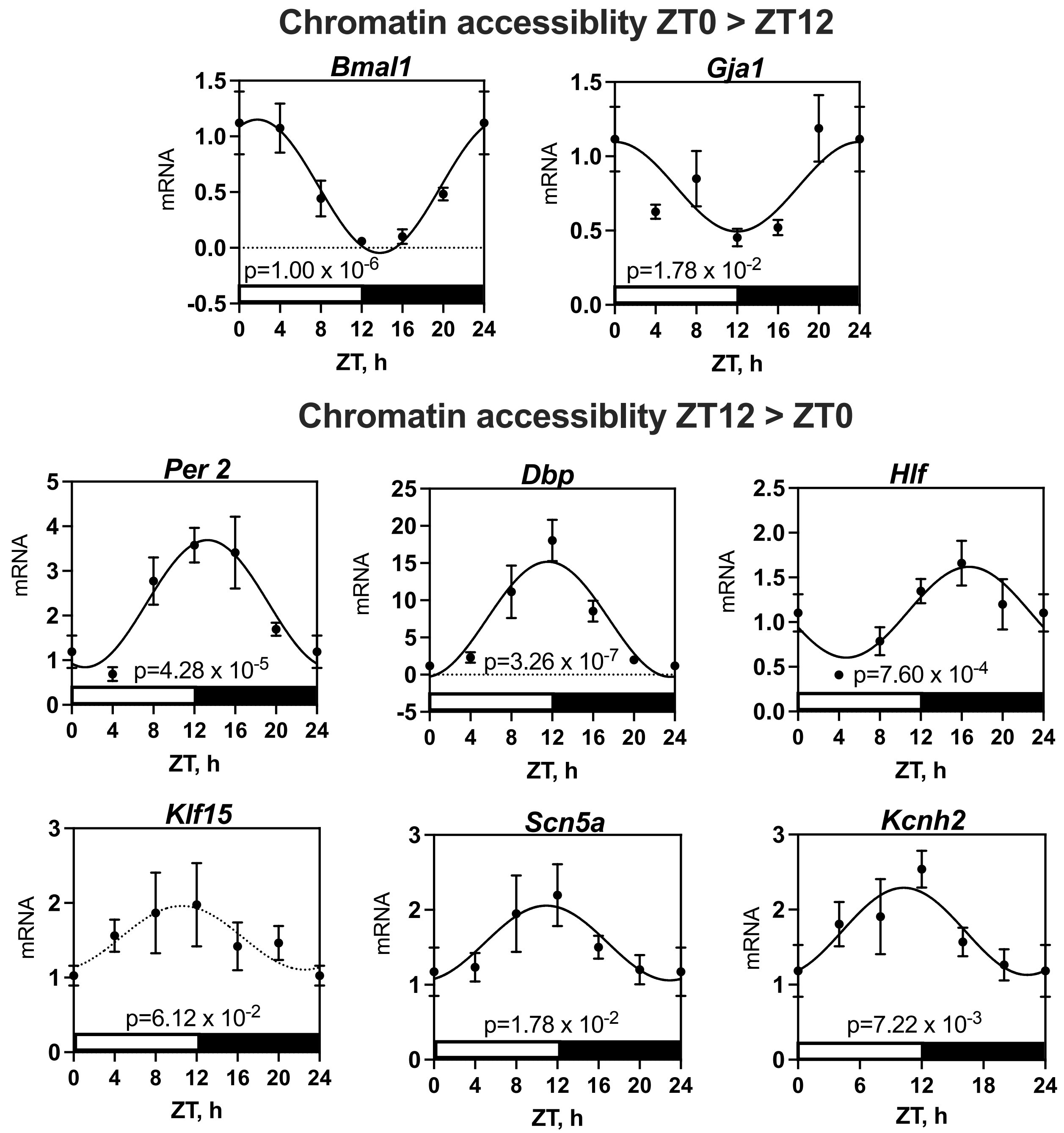

Supplemental Figure S2. Phase alignment between diurnal gene expression profiles and chromatin accessibility. mRNA expression measured in mouse left ventricle biopsies at ZT0, ZT4, ZT8, ZT12, ZT16 and ZT20 for selected genes in which a significant circadian rhythm was previously determined and in which greater chromatin accessibility at ZT0 or ZT12 was observed in this study. Expression normalised to *Ipo8* and *Tbp*. (n=5 hearts per timepoint). Data are displayed normalised to control ZT0 mean, and ZT0 data are replotted at ZT24 as a visual aid only. A significant day-night rhythm (as determined by JTK Cycle; adjusted p value given in plots) is denoted by the fitted sine wave. Sine wave with a dotted line denotes an adjusted p value >0.05. Data from same experiment as in Figure 5D.

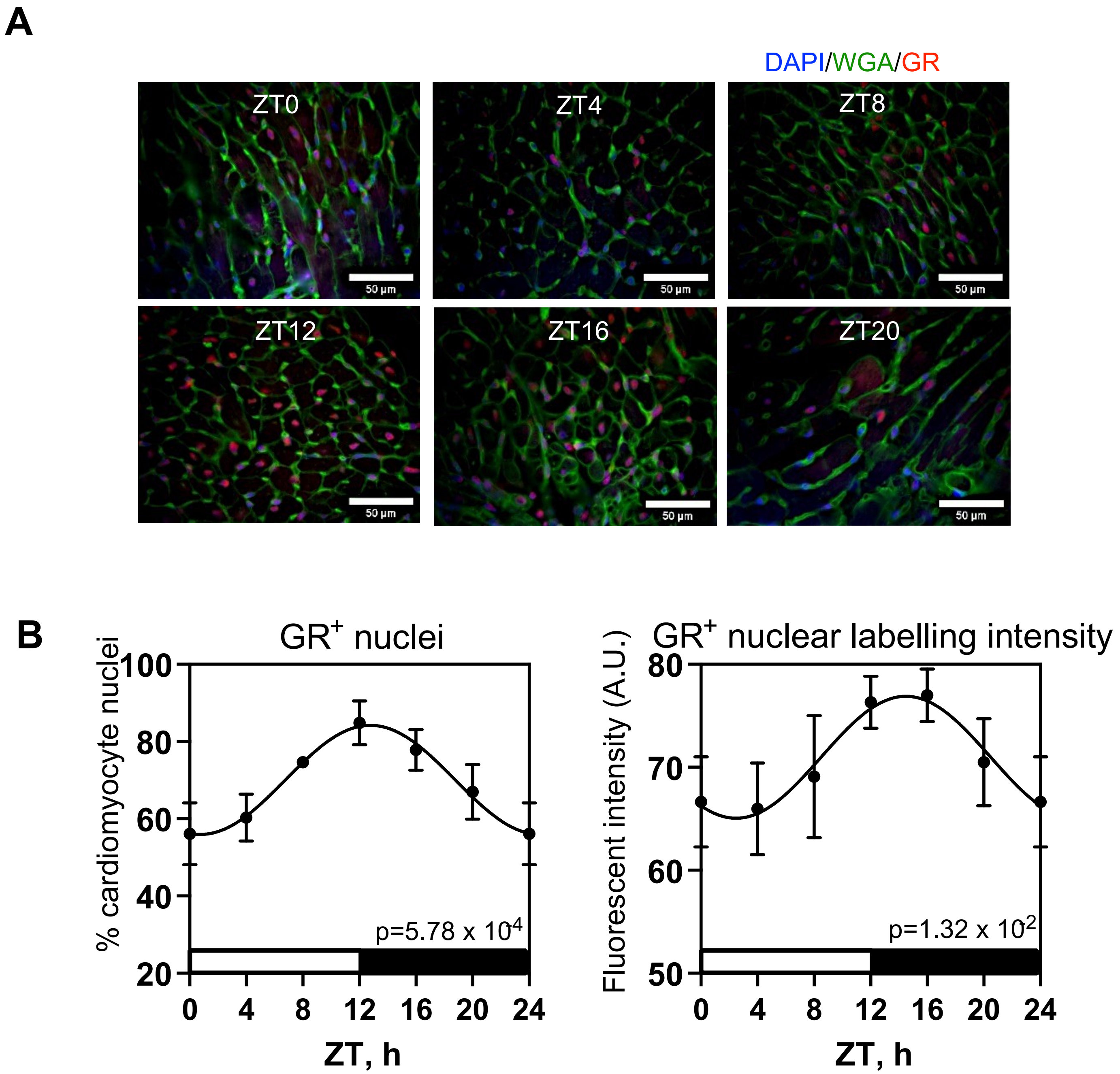

Supplemental Figure S3. A 24 h rhythm in GR nuclear translocation. A, Representative GR immunofluorescent labelling of 10 μm sections of mouse left ventricular biopsies isolated at ZT0, ZT4, ZT8 ZT12, ZT16 and ZT20. Cardiomyocytes were identified by wheat germ agglutinin (green signal) staining and analysed for GR nuclear localisation (red signal) relative to the DAPI label (blue signal). Scale bar = 50 μm. **B,** Summary data derived from images in (A) showing (left) percentage of GR^{+} nuclei relative to total number of $DAPI^{+}$ cardiomyocyte nuclei analysed and (right) signal intensity of GR labelling. 10 images were collected per heart and per timepoint. Mean and SEM from three hearts per time point and ~65 ventricular myocytes per animal are given. A significant day-night rhythm (as determined by JTK Cycle; adjusted p value shown in plot), is denoted by the fitted sine wave.

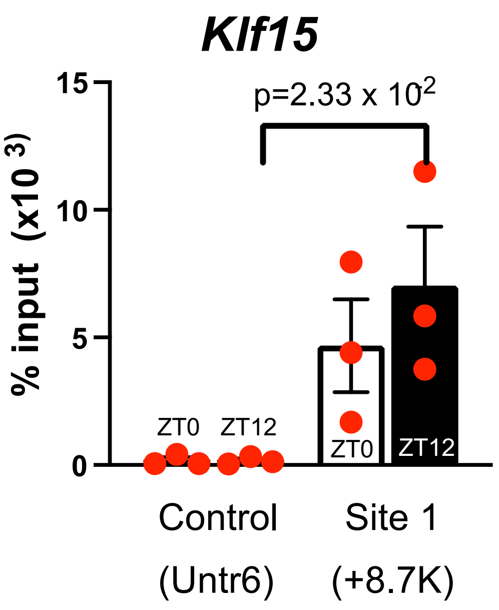

Mouse GGAACATCCTGTTCCAG

Human AGAACGTCCTGTTCCAG

Supplemental Figure S4. GR binding sites on *Klf15*. GR ChIP-qPCR assay testing GR occupancy in a predicted genomic site on *Klf15* using chromatin from mouse left ventricle biopsies harvested at ZT0 and ZT12 (n=3 per time point). ChIP enrichment for site-of-interest and negative control shown. Sequence conservation of site shown. Aligned human GR sites were obtained from the UCSC Genome Browser. Nonconserved base is highlighted in red. Each point is an independent biological replicate. Data were normalized for primer efficiency by carrying out qPCR for individual primer pairs with input DNA isolated and pooled from all samples. p value was determined by a Kruskal-Wallis test with Dunn's test for multiple comparison correction.

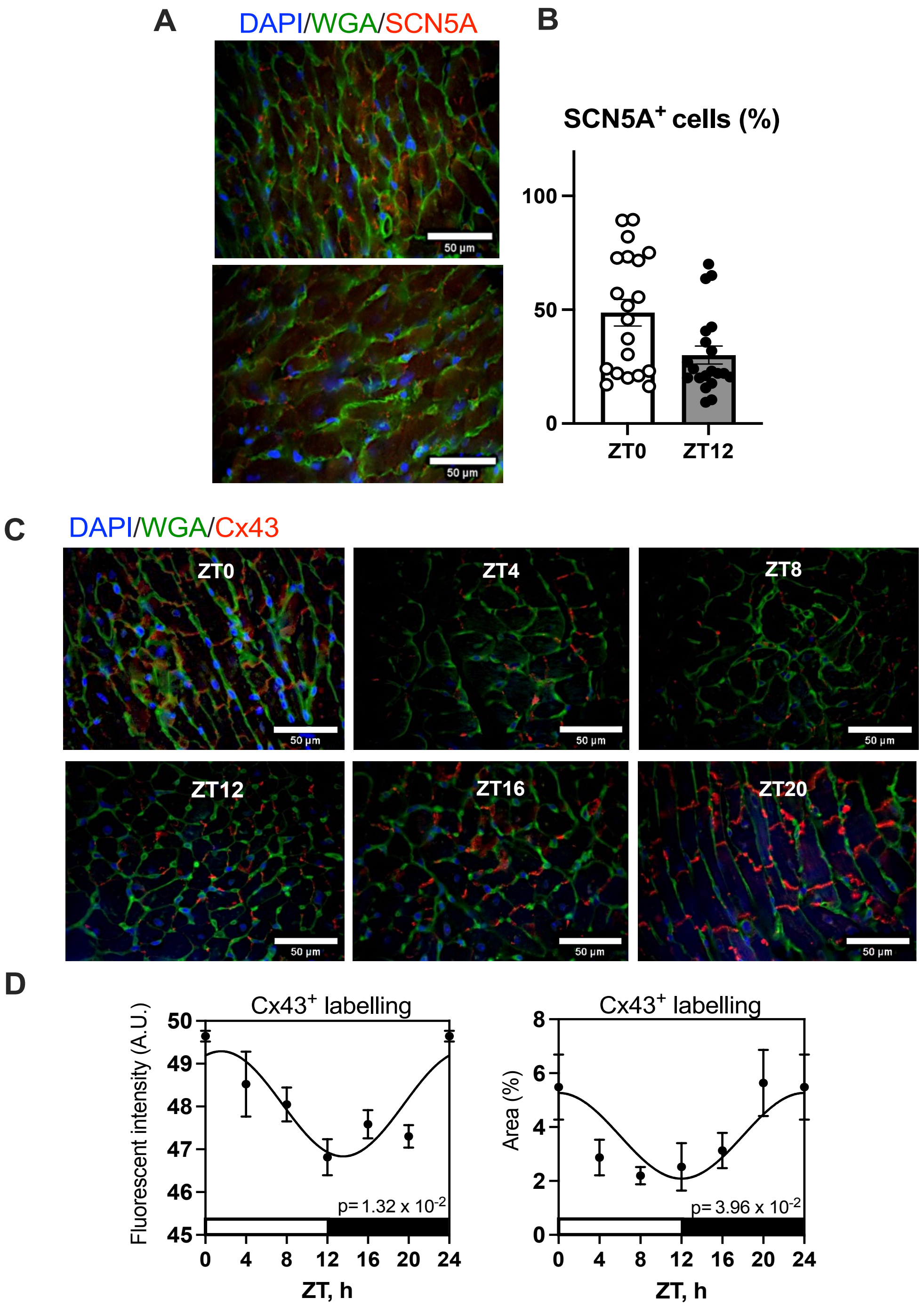

Supplemental Figure S5. Rhythms in protein expression of SCN5A and Cx43. A, Representative antiSCN5A immunofluorescent labelling of 10 µm sections of mouse left ventricular biopsies isolated at ZT0 (upper image) and at ZT12 (lower image). Cardiomyocyte cell membranes were identified by wheat germ agglutinin staining and analysed for SCN5A$^+$ labelling that colocalised with the cell membrane. **B**, Percentage of cardiomyocytes with SCN5A$^+$ labelling at ZT0 and ZT12 derived from images in (A). Each point corresponds to the averaged value per section. ~1227 cardiomyocytes from 5 sections per animal from 4 animals at ZT0 and ~985 cardiomyocytes per from 5 sections per animal from 4 animals were analysed at ZT12. **C**, Representative anti-Cx43 immunofluorescent labelling of 10 µm sections of mouse left ventricular biopsies isolated at ZT0, ZT4, ZT8, ZT12, ZT16 and ZT20. **D**, Summary data from images in (C) showing fluorescent intensity and area fraction of Cx43$^+$ labelling. Each point corresponds to the averaged value of 4 hearts per time point. 17-20 sections per time point were analysed. A significant day-night rhythm (as determined by JTK Cycle; adjusted p value shown in plot) is denoted by the fitted sine wave.

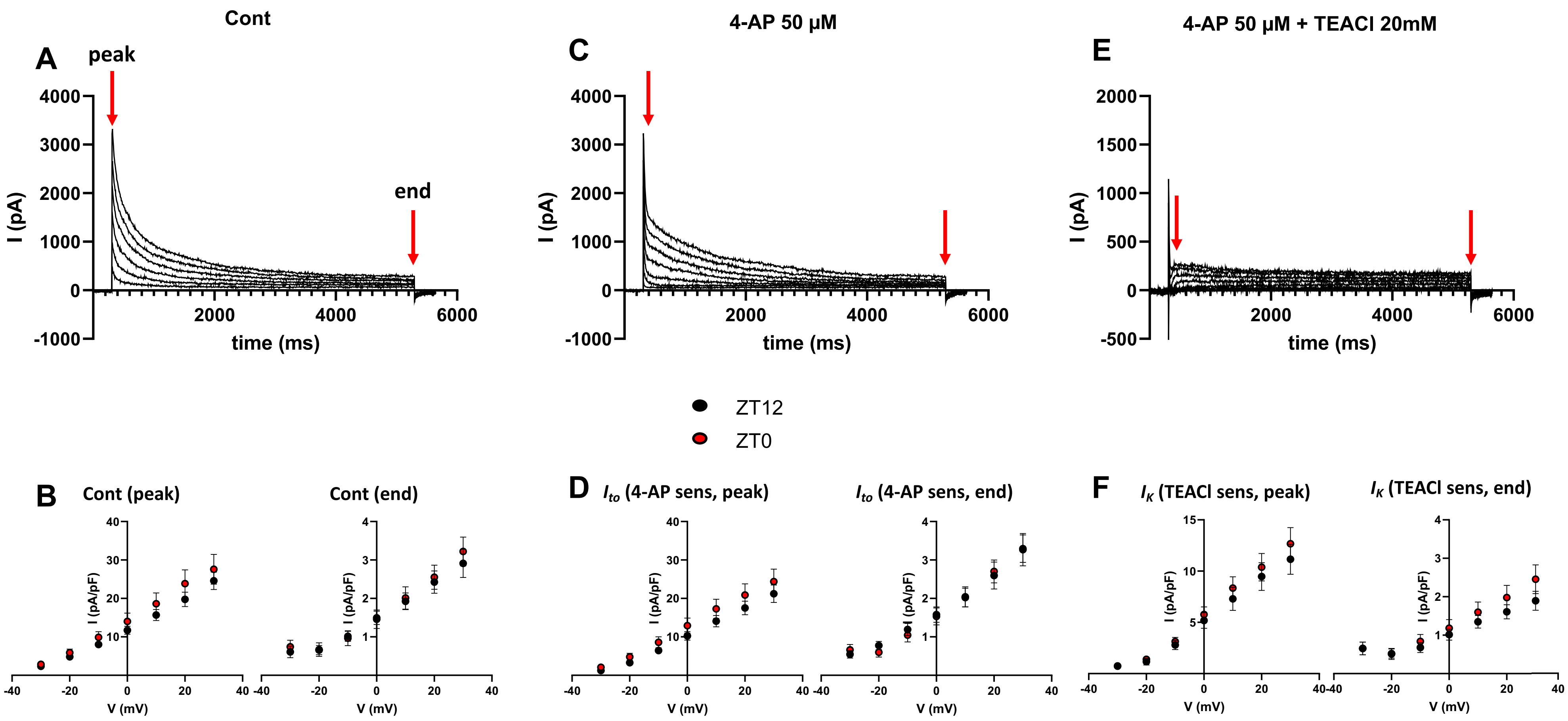

Supplemental Figure S6. Sample traces and current-to-voltage relationships of I_{to} and I_K in ventricular myocytes at ZT0 and ZT12. I_{to} is defined as the current sensitive to 4-amino-pyridine (4-AP). 4-AP-sensitive current is measured at peak and at the end of depolarizing step. The residual current recorded after concomitant application of 4-AP and TEACl is defined as TEACl-resistant I_K. **A**, Sample traces of total membrane current upon depolarization to voltages shown in current-to-voltage relationships in B. **B**, Current-to-voltage relationships of total current at ZT0 (red circle) and ZT12 (black circle). **C**, Samples traces of membrane current in A after perfusion of 4-AP (50 μM). **D**, Current-to-voltage relationships of net 4-AP-sensitive current at ZT0 and ZT12. **E**, Sample traces of total current after concomitant perfusion of 4-AP and TEACl (20 mM). **F**, Current-to-voltage relationships of net current blocked by concomitant perfusion of 4-AP and TEACl (TEACl-sensitive I_K) at ZT0 and ZT12. Arrows indicate points of current measurements at onset of depolarizing step (peak) and at the end of the steps (end). Data are from n=13 ventricular myocytes from n=3 mice at ZT0 (red circles) and from n=9 myocytes from n=2 mice at ZT12 (black circles).

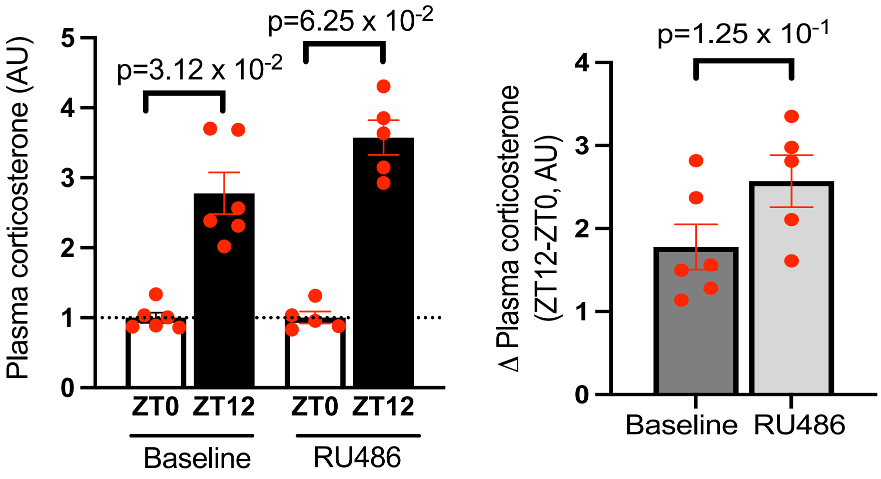

Supplemental Figure S7. Chronic RU486 treatment does not impact the day-night variation in plasma corticosterone. Left, plasma corticosterone measured using ELISA at ZT0 and at ZT12 at baseline and following RU486-adminstration (4 x daily intraperitoneal injections at 20 mg/kg/body weight). Data normalised to ZT0 values. Right, change in corticosterone levels between ZT0 and ZT12 is unaffected by RU486 treatment. Values derived from data in left panel. p values determined by a Wilcoxon matched-pairs signed rank test.

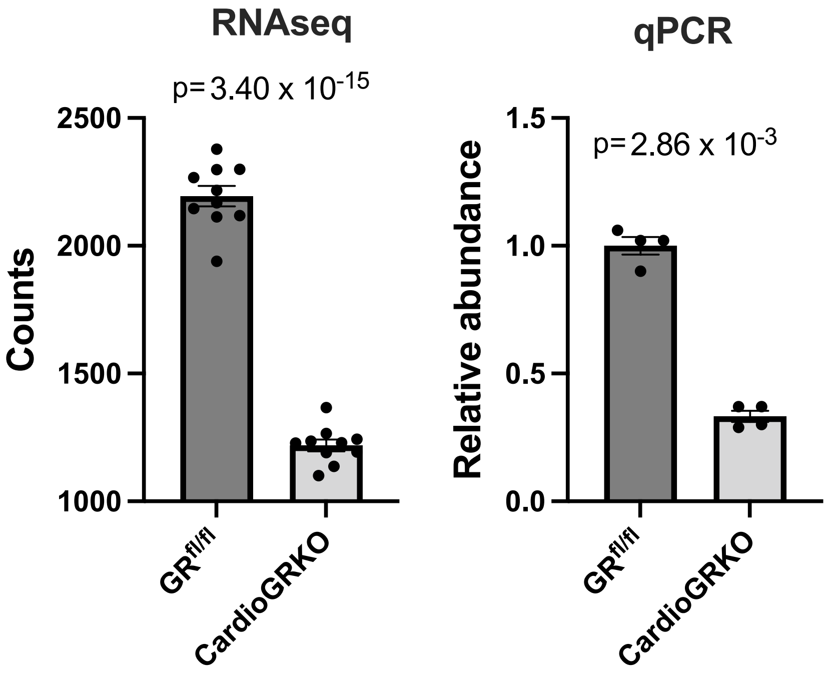

Supplemental Figure S8: GR levels in CardioGRKO mice. GR transcript expression levels in $GR^{fl/fl}$ mice and CardioGRKO mice determined using RNAseq (normalised counts given) and qPCR. qPCR expression normalised to *Ipo8* and *Tbp*. Each point is an independent biological replicate. p values determined by Students *t* test in the case of RNAseq data and a Mann Whitney test in the case of qPCR data.

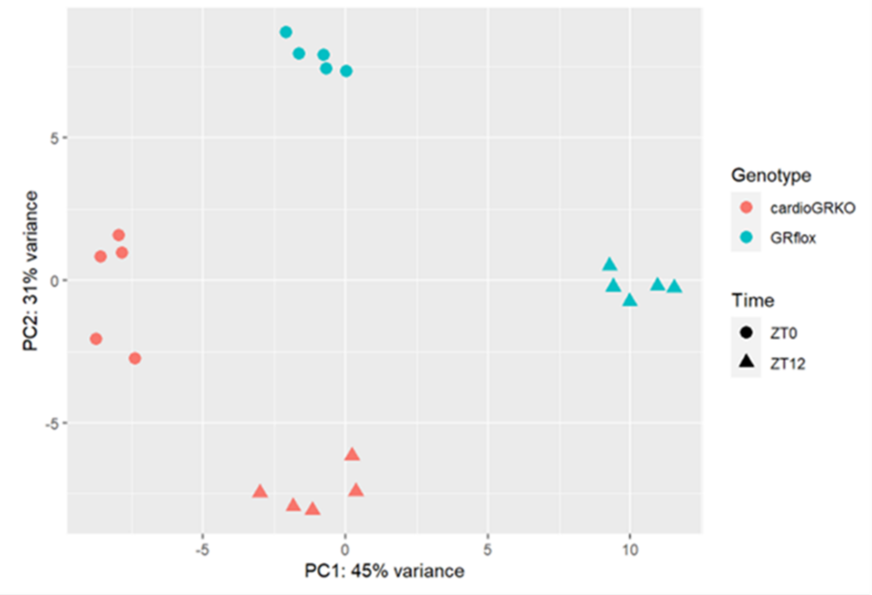

Supplemental Figure S9: Principal component analysis (PCA) on RNAseq data. PCA performed on RNAseq data collected in left ventricular free wall biopsies harvested at ZT0 and ZT12 in $GR^{fl/fl}$ mice and CardioGRKO mice indicating clustering of samples according to their genotype ($GR^{fl/fl}$, blue and cardioGRKO, red) and time of sample collection (ZT0, circle and ZT12, triangle). Proportion of variance determined by Principle Component 1 (PC1) and PC2 shown.

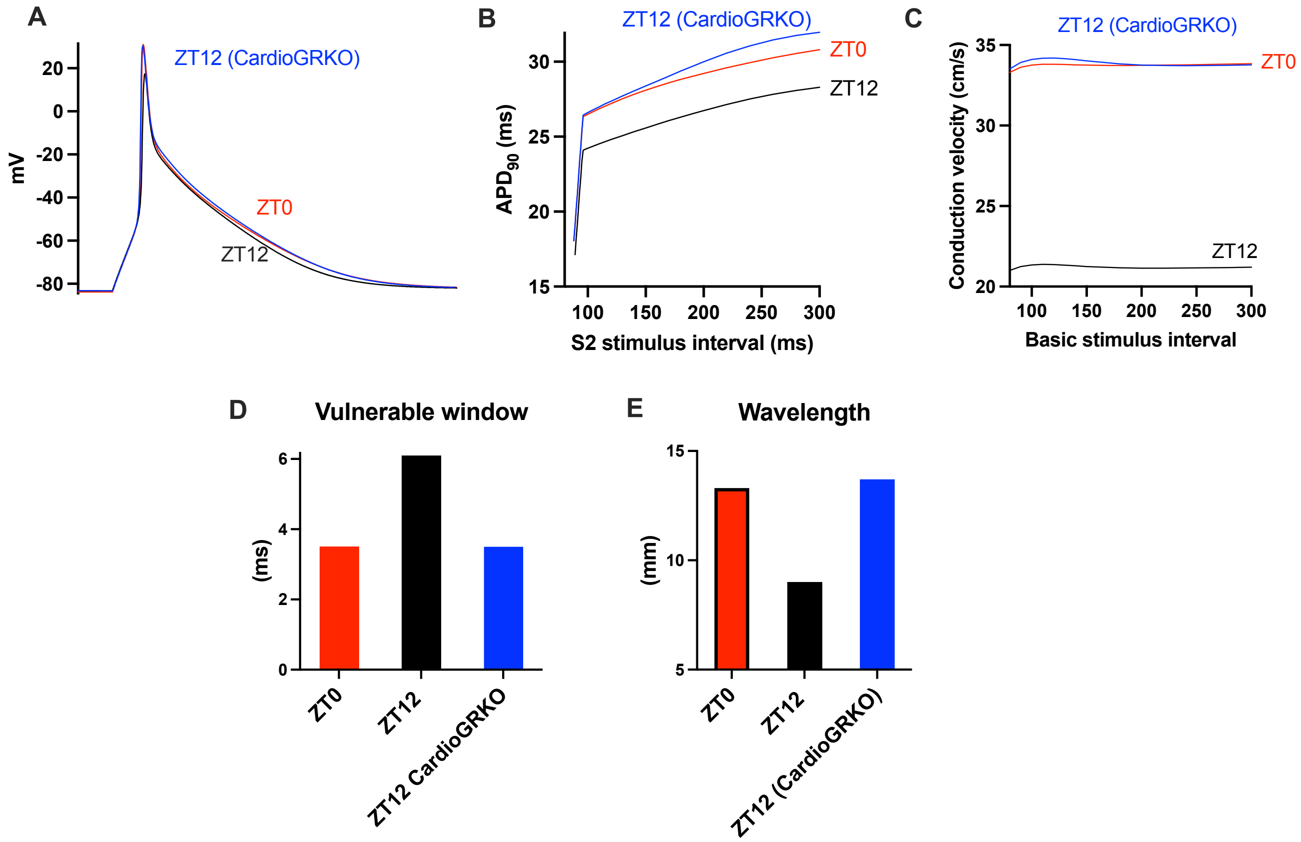

Supplemental Figure S10: Simulated impact of the effect of cardiomyocyte specific GR knockout on the mouse ventricular action potential and susceptibility to triggered ventricular arrhythmia. **A**, Simulated action potentials at a basic stimulus interval of 150 ms at ZT0 (red trace), ZT12 (blue trace) and ZT12 following GR knockout (blue trace). Summary of the properties of the simulated action potentials are given in Table S3. **B**, Simulated action potential restitution curves at ZT0, ZT12 and ZT12 following GR knockout. APD_{90} is plotted against the S2 stimulus interval (basic stimulus interval, 300 ms). **C**, Conduction velocity as a function of the basic stimulus interval at ZT0, ZT12 and ZT12 following GR knockout. **D**, Width of the vulnerability window at a basic stimulus interval of 200 ms at ZT0, ZT12 and ZT12 following GR knockout. **E**, Wavelength of excitation waves at a basic stimulus interval of 200 ms at ZT0 ZT12 and ZT12 following GR knockout. C-E measured from simulations of a one-dimensional string of myocytes.

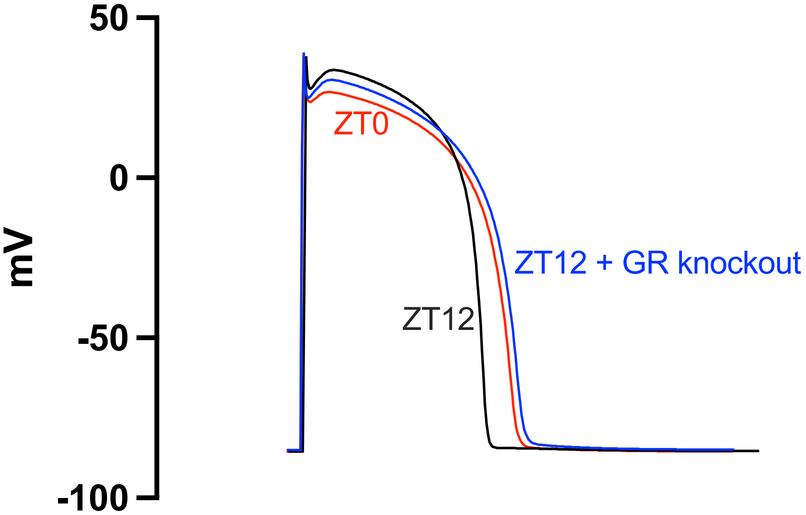

Parameter	ZT0	ZT12	ZT12 + GR knockout
Action potential amplitude (mV)	126.8	117.9	126.6
dV/dt_{max} (V/s)	371.6	229.5	370.6
APD_{90} (ms)	296.9	239.7	305.6

Supplemental Figure S11: Simulated day-night variation in the human ventricular action potential and the effect on this of GR knockout. Simulated action potentials at a basic stimulus interval of 1 s at ZT0, ZT12 and ZT12 following GR knockout shown. The table shows properties of the simulated action potentials in the three conditions.

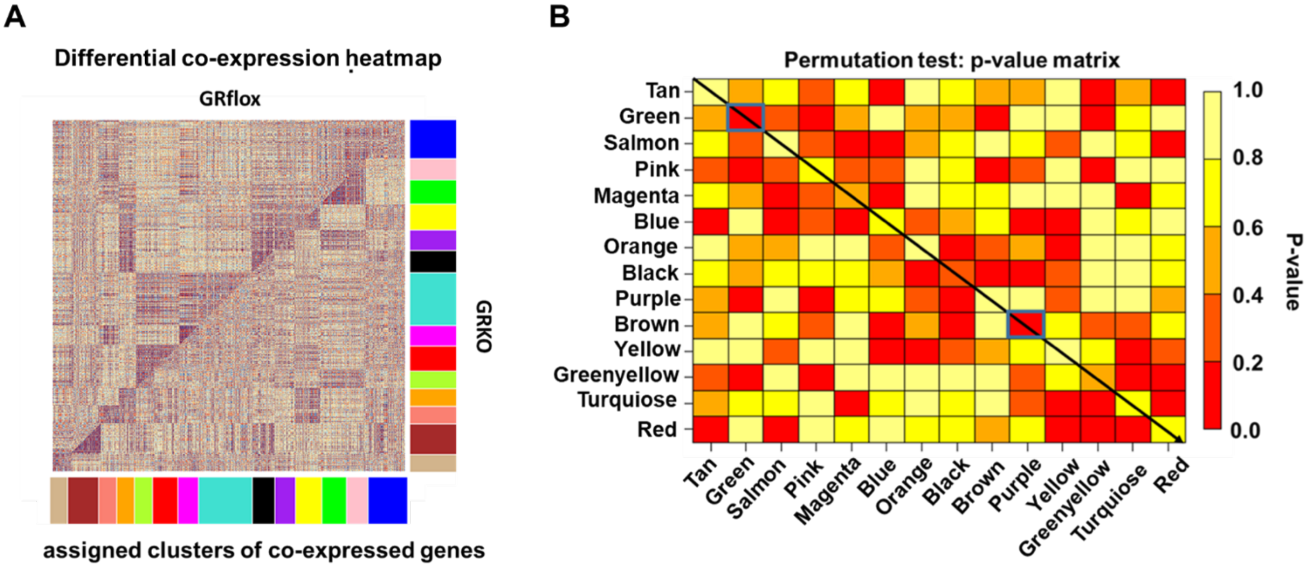

Supplemental Figure S12: Identification of gene co-expression clusters. A, Comparative heatmap demonstrating correlation patterns of gene clusters in $GR^{fl/fl}$ and cardioGRKO conditions. Discrete gene clusters were stratified by individual colors and assigned to x and y axes. Correlation adjacency matrix for counts at ZT12 in the $GR^{fl/fl}$ group are given in top-left half of the matrix, while for cardioGRKO group in the bottom right. Differential co-expression is indicated by magenta whereas beige indicates weak co-expression. **B)** Differential co-expression of gene clusters between $GR^{fl/fl}$ and cardioGRKO left ventricular samples determined by permutation statistical testing for 14 gene clusters. From 10 000 permutations genes in clusters were randomly sampled. p value = proportion of samples that have a test statistic larger than that of our observed data. Resulted matrix of p values plotted as an adjacency matrix. Color mapping on p values is described on the graph (1 –yellow, 0 – red). Diagonal of the matrix gives p values of differential coexpression gene clusters between two conditions. Clusters with p-values <0.05 (highlighted) were considered for further analysis.
