# Supplementary material for: Cardiac GR Mediates the Diurnal Rhythm in Ventricular Arrhythmia Susceptibility
Source: Circ Res. 2024 Mar 27;134(10):1306–26. doi: 10.1161/CIRCRESAHA.123.323464 (PMC11081863; doi:10.1161/CIRCRESAHA.123.323464)
Supplement: Supplementary file 3 [file res-134-1306-s003.pdf]

Major Resources Table

In order to allow validation and replication of experiments, all essential research materials listed in the Methods should be included in the Major Resources Table below. Authors are encouraged to use public repositories for protocols, data, code, and other materials and provide persistent identifiers and/or links to repositories when available. Authors may add or delete rows as needed.

Animals (in vivo studies)

Species	Vendor or Source	Background Strain	Sex	Persistent ID / URL
Mouse	Envigo, UK	C57BL/6J	male	https://www.envigo.com/model/c57bl6jolahsd

Genetically Modified Animals

	Species	Vendor or Source	Background Strain	Persistent ID / URL
**Parent - Male**	GRfl/fl mice generated by 7 generations backcrossing into C57Bl/6 mice	Signal Transduction Laboratory, NIEHS, NIH, DHHS, 111 TW Alexander Drive, RTP, NC 27709	C57BL/6NJ	https://www.ncbi.nlm.nih.gov/pmc/articles/PMC7082727/
**Parent - Female**	cardiomyocyte-specific αMHCCre/+ mice	The Jackson Laboratory, 011038	C57BL/6NJ	https://www.ncbi.nlm.nih.gov/pmc/articles/PMC7082727/

Antibodies

Target antigen	Vendor or Source	Catalog #	Working concentration	Lot # (preferred but not required)	Persistent ID / URL
GR	Cell Signaling Technology	3660S	1:1000	5	https://www.cellsignal.com/products/primary-antibodies/glucocorticoid-receptor-d8h2-xp-rabbit-mab/3660

DOI [to be added]

Scn5a	Alomone Labs	ASC-005	1:200	ASC005AN4325	https://www.alomone.com/p/anti-nav1-5/ASC-005
Cx43	Alomone Labs	ACC-201	1:100		https://www.alomone.com/p/anti-connexin-43/ACC-201
Wheat germ agglutinin (WGA)	Sigma-Aldrich	L4895	1:100		https://www.sigmaaldrich.com/KZ/en/product/sigma/l4895
Alexa Fluor 594 goat anti-rabbit IgG	Thermofisher Scientific	A32740	1:250	2506100	https://www.thermofisher.com/antibody/product/Goat-anti-Rabbit-IgG-H-L-Highly-Cross-Adsorbed-Secondary-Antibody-Polyclonal/A32740
Scn5a	Affinity Biosciences	DF13217	1:500	39290	https://www.affbiotech.com/goods-16595-DF13217-Nav1_5_Antibody.html
Kcnh2	Thermofisher Scientific	OSP00150W	1:1000	Rb1658-211110-ws	https://www.thermofisher.com/antibody/product/KCNH2-Antibody-Polyclonal/OSP00150W-100UL

DOI [to be added]

Anti-rabbit IgG, HRP-linked Antibody	Cell Signaling Technologies	7074	1:3333	0	https://www.cellsignal.com/products/secondary-antibodies/anti-rabbit-igg-hrp-linked-antibody/7074

Data & Code Availability

Description	Source / Repository	Persistent ID / URL
RNA-seq data	Gene Expression Omnibus	GSE236548
ATAC-seq data	ArrayExpress	E-MTAB-13696

DOI [to be added]

ARRIVE GUIDELINES

The ARRIVE guidelines (https://arriveguidelines.org/) are a checklist of recommendations to improve the reporting of research involving animals. Key elements of the study design should be included below to better enable readers to scrutinize the research adequately, evaluate its methodological rigor, and reproduce the methods or findings.

Study Design

Groups	Sex	Age	Number (prior to experiment)	Number (after termination)	Littermates (Yes/No)
C57BL/6J mice ZT0 *vs*. ZT12	male	10-12 weeks	60	60	Yes
Vehicle-treated mice Time series study (6 timepoints)	male	8-12 weeks	30 mice, 5 mice per timepoint	30	Yes
RU486-treated mice Time series study (6 timepoints)	male	8-12 weeks	30 mice, 5 mice per timepoint	30	Yes
cardioGRKO mice Time series study (6 timepoints)	male	9-16 weeks	30 mice, 5 mice per timepoint	30	Yes
$GR^{fl/fl}$ mice Time series study (6 timepoints)	male	9-16 weeks	30 mice, 5 mice per timepoint	30	Yes

Sample Size: Animal numbers were estimated *a priori* and determined from power calculations with 80% power and a 95% confidence interval using standard deviation values from our previously published work on the diurnal rhythm in cardiac electrophysiological and transcriptional parameters in mice (e.g. PMIDs 37122216, 33574422, 33278629). The sample size for ATAC-seq analysis in cardiomyocytes was in keeping with encode guidelines that mandate two or more biological replicates. In this study we used three pooled biological replicates per sample and two independent samples per time point.

Inclusion and Exclusion Criteria: The exclusion criteria set *a priori* was that any animal demonstrating sustained changes in behavior, surgical complications, or other adverse effects resulting from administration of the GR blocker RU486 would be humanely culled and excluded from analysis. However, this humane endpoint was not reached in any case and no animal was excluded from analysis.

Randomization
Animals were housed in a random order on shelves. Animals were randomised into ZT groups, and vehicle or RU486-treated groups using a random number generator. Measurements were made in a random order.

Blinding
Blinding was performed during ATAC-seq analysis and in all Langendorff/arrhythmia inducibility studies.

Please also see attached ARRIVE guidelines 2.0 author checklist

DOI [to be added]
