# Supplementary material for: Cardiac GR Mediates the Diurnal Rhythm in Ventricular Arrhythmia Susceptibility
Source: Circ Res. 2024 Mar 27;134(10):1306–26. doi: 10.1161/CIRCRESAHA.123.323464 (PMC11081863; doi:10.1161/CIRCRESAHA.123.323464)
Supplement: Supplementary file 4 [file res-134-1306-s004.pdf]

Figure	n (animals)	Normal distribution (Shapiro-Wilk test)	Statistical test	Parameter	p value
**Figure 1**					
**1A**	n = 2 for each timepoint	Not assessed	DESeq2 comparison of log2 fold change between normalised counts (Wald test with Benjamini Hochberg q-value correction)	Chromatin accessiblity	p=2.09E-03;FDR <0.1
**1C**	n = 2 for each timepoint	Not applicable	Binomial test, Benjamini-Hochberg q-value	Process pathways annotated from DA peaks	FDR <0.05
**1D**	n = 6 for each timepoint	Data from Zhang et al. 2014	JTK cycle adjusted p value	Gene expression phase for rhythmic genes in DA	FDR <0.05
**Figure 2**					
**2A**	n = 2 for each timepoint	Not applicable	SeqPos Z-score	Enriched TF motifs at ZT0	FDR <0.05
		Not applicable		Enriched TF motifs at ZT12	FDR <0.05
**2B (right)**	n = 4 for each timepoint	Yes	Mann-Whitney test	GR+ nuclei (%)	2.86E-02
				GR Intensity	5.71E-02
**2D**	212 GR targets	Not applicable	Binomial test, Benjamini-Hochberg q-value	GO enrichment	FDR <0.05
**2E (middle)**	n = 3 for each timepoint	Yes	Kruskal-Wallis test with Dunn's multiple comparisons test	GR occupancy of predicted genomic sites at Kcnh2 vs Control	7.40E-03
		Yes		GR occupancy of predicted genomic sites at Scn5a vs Control	4.70E-03
		Yes		GR occupancy of predicted genomic sites at Per2 vs Control	4.12E-02
**Figure 3**					

**3B (right)**	n = 4 for each timepoint	Yes	Mann-Whitney test	Scn5a protein abundance	2.86E-02
**3D**	13 cells from n = 3 for each timepoint	Yes	Linear effects mixed model followed by Sidak's multiple comparisons test	INa, -40 mV	2.34E-02
				INa, -35 mV	6.47E-05
				INa, -30 mV	1.38E-06
				INa, -25 mV	3.48E-07
				INa, -20 mV	3.97E-07
				INa, -15 mV	1.40E-06
				INa, -10mV	2.52E-05
				INa, -5mV	1.10E-03
**3E (left)**	13 cells from n = 3 for each timepoint	Yes	Nested T-test	INa peak density at -30mV	2.28E-03
**3G (left)**	n = 4 for each timepoint	Yes	JTK cycle adjusted p value	Kcnh2 rhythmic protein expression	1.61E-02
**3G (right)**	n = 4 for each timepoint	Yes	Mann-Whitney test	Kcnh2 protein abundance	5.71E-02
**3I**	n = 3 for each timepoint, 8 cells for ZT0 and 9 cells for ZT12	Yes	Linear effects mixed model followed by Sidak's multiple comparisons test	IKr, 20mV	1.73E-02
				IKr, 30mV	3.63E-02
				IKr, 40mV	2.52E-05
				IKr, 50mV	1.05E-07
**Figure 5**					
**5B (Vehicle)**	n = 5 for each timepoint and treatment	Yes	JTK cycle adjusted p value	Corticosterone (ng/ml) in Vehicle treated mice	9.91E-05
**5B (RU486)**				Corticosterone (ng/ml) in RU486 treated mice	7.11E-02

**5C**	n = 3 for each timepoint and treatment	Yes	JTK cycle adjusted p value	GR+ cardiomyocytes (%) in LV of Vehicle treated mice	5.78E-04
				GR+ cardiomyocytes (%) in LV of RU486 treated mice	4.26E-01
**5D**	n = 5 for each timepoint and treatment	Yes	JTK cycle adjusted p value	Per2 mRNA normalized expression in Vehicle treated mice	4.28E-05
				Per2 mRNA normalized expression in RU486 treated mice	6.75E-04
				Bmal1 mRNA normalized expression in Vehicle treated mice	1.00E-06
				Bmal1 mRNA normalized expression in RU486 treated mice	2.85E-08
				Scn5a mRNA normalized expression in Vehicle treated mice	1.78E-02
				Kcnh2 mRNA normalized expression in Vehicle treated mice	7.22E-03
**5F**	n = 5 for each timepoint and treatment	No	χ2 test	Percentage of inducible VA in vehicle at ZT0 and ZT12	5.78E-02
				Percentage of inducible VA in vehicle at ZT12 and RU486 ZT12	9.82E-03
**Figure 6**					
**6B**	n = 5 hearts/group at ZT0 and ZT6; n=7 at ZT12	No	χ2 test	Percentage of inducible VA in GRfl/fl and cardioGRKO mice at ZT12	1.19E-03

**6C**	n = 4 for each genotype	Yes	Zero amplitude F test from Cosinor analysis	Circadian rhythm in the heart rate of GRfl/fl mice	3.00E-05
				Circadian rhythm in the heart rate of cardioGRKO mice	2.83E-01
**6D (left)**	n = 4 for each genotype	No	Mann-Whitney test	Mesor of the heart rate rhythm in GRfl/fl and cardioGRKO mice	2.85E-02
**6E**	n = 4 for each genotype and timepoint	Yes	2-way ANOVA with Tukey's multiple comparisons test	Heart rate in GRfl/fl mice	4.64E-02
				Heart rate in cardioGRKO mice	2.13E-01
**6F**				PR interval in GRfl/fl mice	8.31E-02
				PR interval in cardioGRKO mice	9.48E-01
**6H**				QT interval in GRfl/fl mice	9.76E-02
				QT interval in cardioGRKO mice	3.10E-01
**Figure 7**					
**7A**	n = 5 for each timepoint and genotype	Not assessed	DESeq2 comparison of log2 fold change between normalised counts (Wald test with Benjamini Hochberg q-value correction)	Differentially expressed genes	FDR<0.05

**7D**	n = 5 for each timepoint and genotype	Yes	JTK cycle adjusted p value	Bmal1 mRNA normalized expression in GRfl/fl mice	2.09E-15
		Yes		Bmal1 mRNA normalized expression in cardioGRKO mice	2.76E-11
		Yes		Clock mRNA normalized expression in GRfl/fl mice	4.76E-08
		No		Clock mRNA normalized expression in cardioGRKO mice	2.88E-04
		Yes		Cry1 mRNA normalized expression in GRfl/fl mice	3.64E-12
		Yes		Cry1 mRNA normalized expression in cardioGRKO mice	6.20E-08
		No		Cry2 mRNA normalized expression in GRfl/fl mice	2.25E-06
		Yes		Cry2 mRNA normalized expression in cardioGRKO mice	1.81E-07
		Yes		Dbp mRNA normalized expression in GRfl/fl mice	3.64E-12
		Yes		Dbp mRNA normalized expression in cardioGRKO mice	1.70E-13

**7E**	n = 5 for each timepoint and genotype	No	JTK cycle adjusted p value	Per1 mRNA normalized expression in GRfl/fl mice	1.62E-06
		No		Per1 mRNA normalized expression in cardioGRKO mice	3.16E-04
		Yes		Per2 mRNA normalized expression in GRfl/fl mice	1.78E-13
		Yes		Per2 mRNA normalized expression in cardioGRKO mice	3.64E-12
		Yes		Scn5a mRNA normalized expression in GRfl/fl mice	1.87E-03
		Yes		Scn5a mRNA normalized expression in cardioGRKO mice	3.55E-02
		Yes		Kcnh2 mRNA normalized expression in GRfl/fl mice	3.56E-05
		Yes		Kcnh2 mRNA normalized expression in cardioGRKO mice	3.55E-02
		Yes		Gja1 mRNA normalized expression in GRfl/fl mice	3.60E-05
		No		Gja1 mRNA normalized expression in cardioGRKO mice	1.00E+00
**7F**	n = 5 for each timepoint and genotype	Yes	JTK cycle adjusted p value	Clock amplitude of mRNA 24 h rhythm	7.93E-03
				Per1 amplitude of mRNA 24 h rhythm	3.17E-02
				Per2 amplitude of mRNA 24 h rhythm	1.58E-02
				Scn5a amplitude of mRNA 24 h rhythm	5.50E-02
				Kcnh2 amplitude of mRNA 24 h rhythm	3.17E-02
				Gja1 amplitude of mRNA 24 h rhythm	5.50E-02

**Figure 8**					
**8A**	n = 5 for each timepoint and genotype	Not applicable	Fisher's exact test, Benjamini-Hochberg q-value	KEGG enrichment	FDR < 0.05
**8B**	n = 5 for each timepoint and genotype	Not applicable	Fisher's exact test, Benjamini-Hochberg q-value	KEGG enrichment	FDR < 0.05
**8C**	n = 5 for each timepoint and genotype	Not applicable	Hypergeometric test	PPI enrichment	8.05E-17
**SUPPLEMENTAL FIGURE I**					
**S1I**	n =2 for each timepoint; 52,510 consensus regions	No (Anderson Darling and D'Agostino Pearson test)	Spearman correlation	Reproduciblity between samples for ATAC coverage	all *p* values given
**S1J**					
**SUPPLEMENTAL FIGURE 2**					

S2	n = 5 for each timepoint	Yes	JTK cycle adjusted p value	Bmal1 mRNA normalized expression	1.00E-06
		Yes		Gja1 mRNA normalized expression	1.78E-02
		Yes		Per2 mRNA normalized expression	4.28E-05
		Yes		Dbp mRNA normalized expression	3.26E-07
		Yes		Hlf mRNA normalized expression	7.60E-04
		No		Klf15 mRNA normalized expression	6.12E-02
		Yes		Scn5a mRNA normalized expression	1.78E-02
		Yes		Kcnh2 mRNA normalized expression	7.22E-03
**SUPPLEMENTAL FIGURE 3**					
**S3B (left)**	n = 4 for each timepoint	Yes	JTK cycle adjusted p value	Percentage of GR+ nuclei	5.78E-04
**S3B (right)**				Intensity of GR staining	1.32E-02
**SUPPLEMENTAL FIGURE 4**					
**S4**	n = 3 for each timepoint	Yes	Kruskal-Wallis test with Dunn's multiple comparisons test	GR occupancy of predicted genomic sites at Klf15 at ZT12	2.33E-02
**SUPPLEMENTAL FIGURE 5**					
**S5B**	n = 4 for each timepoint; 20 sections for each timepoint; 1227 CMs at ZT0 and 985 CMs at ZT12	No	Nested t-test	Percentage of Scn5a+ cardiomyocytes	N.S.
**S5D (left)**	n = 4 for each	Yes	JTK cycle adjusted p value	Cx43 labelling	1.32E-02

**S5D (right)**	timepoint; 20 sections for each timepoint	No		Area fraction of Cx43+ labeling	3.96E-02
**SUPPLEMENTAL FIGURE 7**					
**S7**	n = 5 for each timepoint and treatment	Yes	Wilcoxon matched-pairs signed rank test	Plasma corticosterone normalized to ZT0 in vehicle treated mice	3.12E-02
				Plasma corticosterone normalized to ZT0 in RU486 treated mice	6.25E-02
**SUPPLEMENTAL FIGURE 8**					
**S8 (left)**	n = 5 for each timepoint and genotype	Yes	Student's t-test	GR transcript expression, normilzed CPM	3.40E-15
**S8 (right)**	n = 4 each timepoint and genotype	Yes	Mann-Whitney test	GR mRNA abundance	2.86E-03
**SUPPLEMENTAL FIGURE 12**					
**S12**	n = 5 for each timepoint and genotype	Not applicable	Permutation test	Differential co-expression gene clusters	<0.05
