# Supplementary material for: Cardiac GR Mediates the Diurnal Rhythm in Ventricular Arrhythmia Susceptibility
Source: Circ Res. 2024 Mar 27;134(10):1306–26. doi: 10.1161/CIRCRESAHA.123.323464 (PMC11081863; doi:10.1161/CIRCRESAHA.123.323464)

UNEDITED WESTERN BLOTS
Figure 3A-B
(SCN5A, ~200 kDa, SAMPLES 1 AND 2)

Stain-free gel

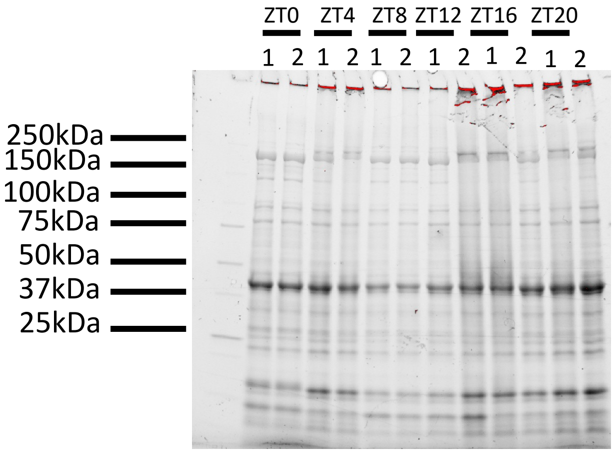

Stain-free blot

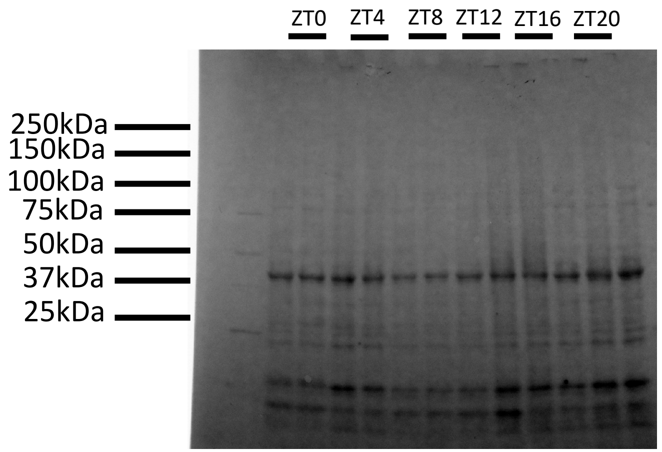

Anti-SCN5a

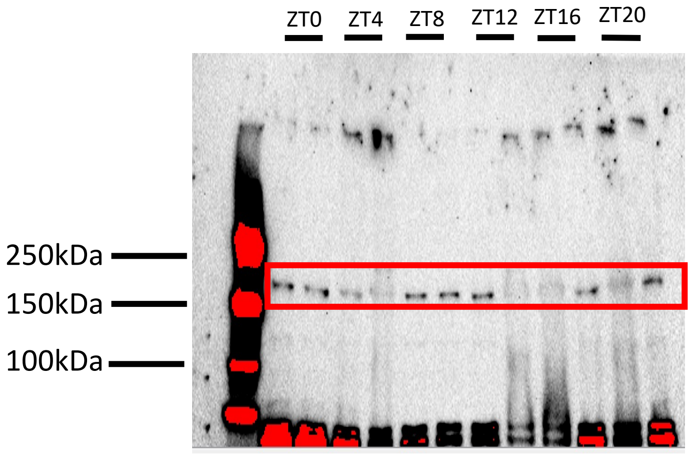

Technical replicate Samples 1 and 2

Stain-free gel

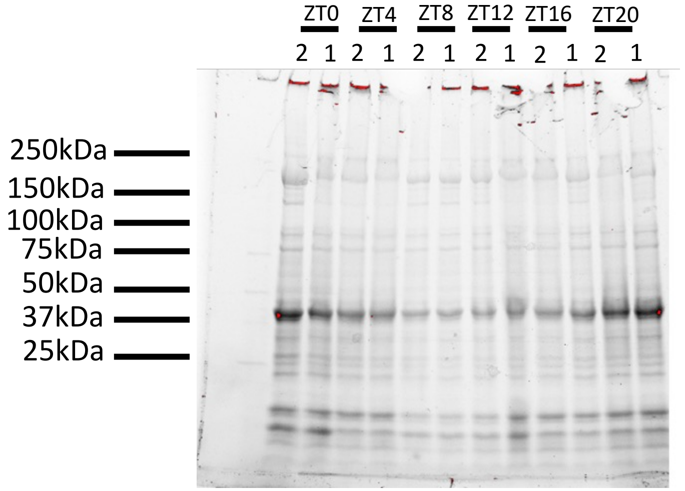

Stain-free blot

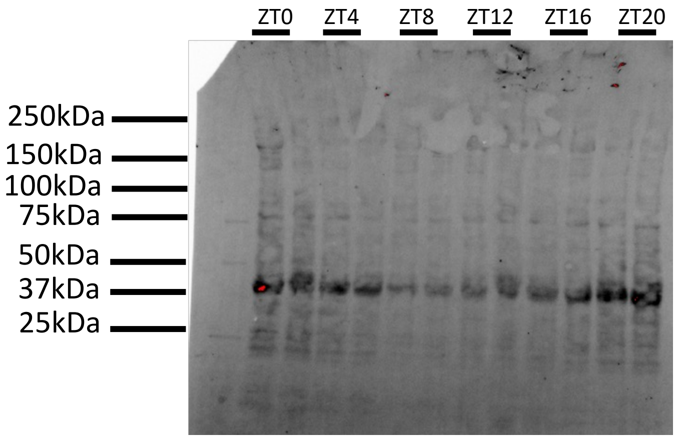

Anti-SCN5a

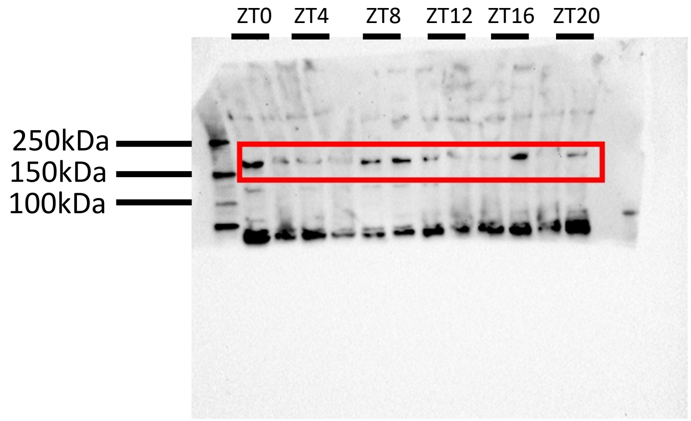

Figure 3A-B (SCN5A – SAMPLES 3 AND 4)

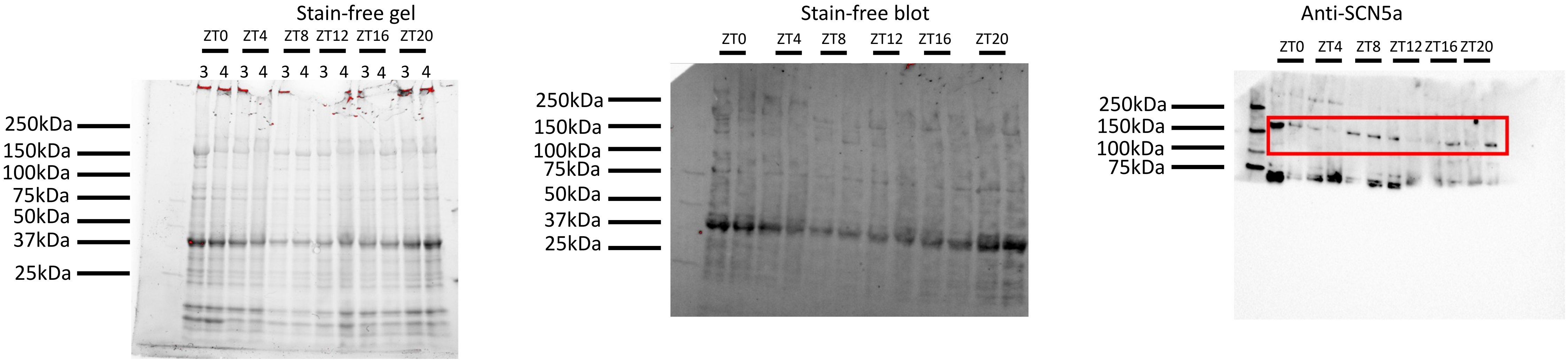

Technical replicate Samples 3 and 4

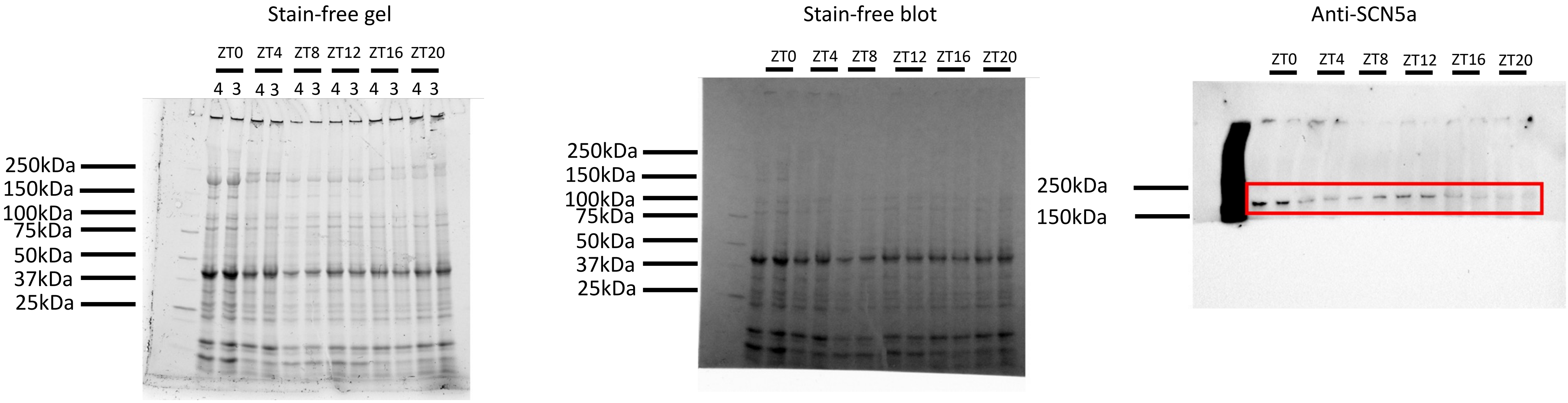

Figure 3F-G (KCNH2, ~75 kDa – SAMPLES 1 AND 2)

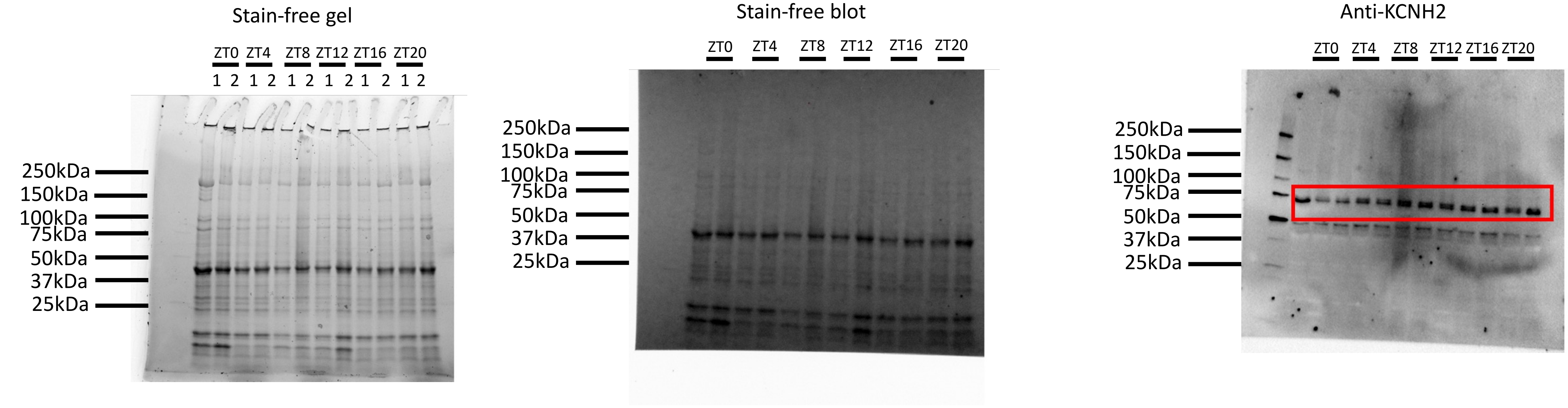

Technical replicate Samples 1 and 2

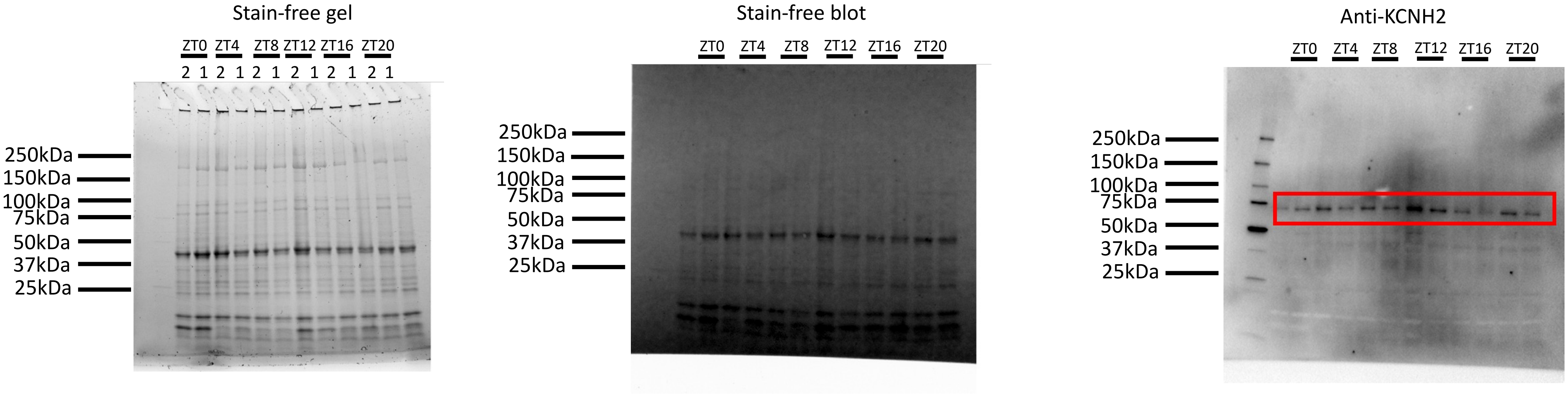

Figure 3F-G (KCNH2 – SAMPLES 3 AND 4)

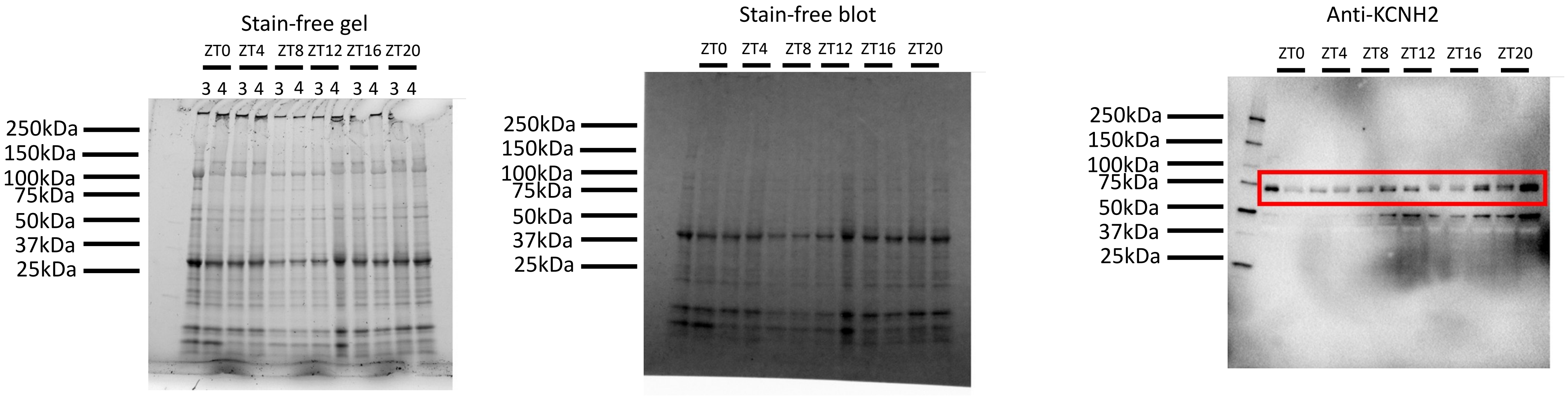

Technical replicate Samples 3 and 4

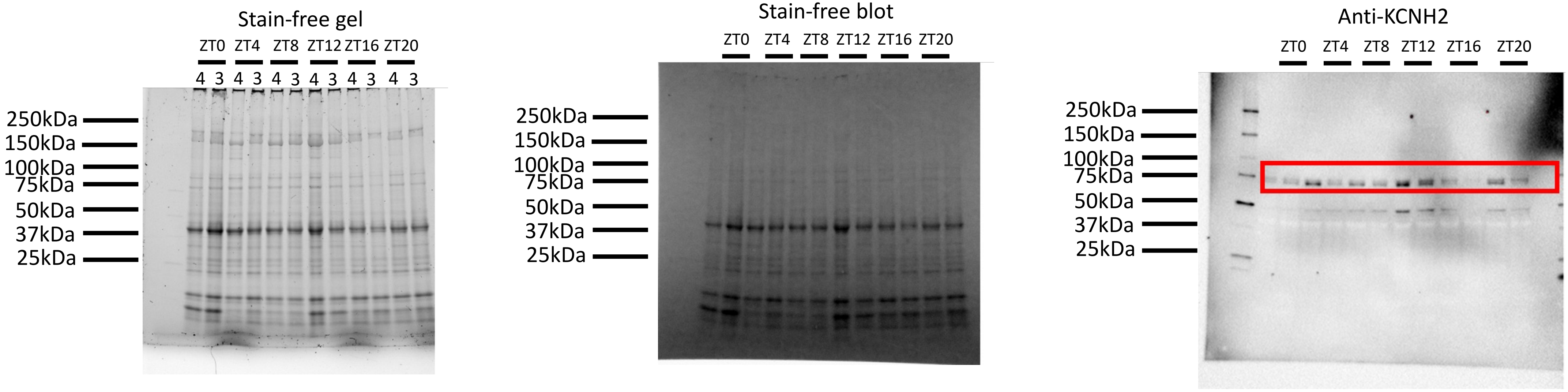

Supplement: Supplementary file 5 [file res-134-1306-s005.pdf]
